# Neural and behavioural correlates of theory of mind reasoning in five-year-old children born preterm

**DOI:** 10.1162/IMAG.a.1347

**Published:** 2026-08-26

**Authors:** Selina Abel, Melissa Thye, Katie Mckinnon, Rebekah Smikle, Jean Skelton, Lorena Jiménez-Sánchez, Ray Amir, Gayle Barclay, Charlotte Jardine, Donna Mcintyre, Iona Hamilton, Yu Wei Chua, Aditi Hosangadi, Alan Quigley, G. David Batty, Michael J. Thrippleton, Heather C. Whalley, James P. Boardman, Hilary Richardson

**Affiliations:** Centre for Reproductive Health, Institute for Regeneration and Repair, University of Edinburgh, Edinburgh, United Kingdom; Institute for Neuroscience and Cardiovascular Research, University of Edinburgh, Edinburgh, United Kingdom; School of Philosophy, Psychology, and Language Sciences, University of Edinburgh, Edinburgh, United Kingdom; Edinburgh Imaging Facility, Royal Infirmary of Edinburgh, Edinburgh, United Kingdom; Department of Public Health, Policy and Systems, Institute of Population Health, University of Liverpool, Liverpool, United Kingdom; Department of Psychology, University of Wisconsin-Madison, Madison, WI, United States; Department of Radiology, Royal Hospital for Children and Young People, Edinburgh, United Kingdom; Department of Epidemiology and Public Health, University College London, London, United Kingdom

**Keywords:** theory of mind, functional MRI (fMRI), social cognition, cognitive development, preterm birth

## Abstract

Behavioural studies suggest atypical or delayed development of “theory of mind” (ToM; our ability to reason about others’ mental states) following preterm birth. Using pre-registered analyses of behavioural and movie-viewing functional magnetic resonance imaging (fMRI) metrics of ToM, we tested for a domain-specific impact of preterm birth (24–32 weeks’ gestational age) on theory of mind development at age 5 years. Preterm-born children (*n* = 52) scored lower than term-born comparators (*n* = 58) on a linguistic behavioural ToM task, but this difference was primarily driven by differences in receptive language. Neurally, preterm-born children (*n* = 30) had qualitatively similar responses in brain regions that support ToM reasoning to a short movie to term-born comparators (*n* = 46), as characterised by four neural metrics; responses to one scene differed as a function of gestational age. Using intersubject correlation analyses, we found that preterm-born children’s ToM network responses were more heterogenous than term-born children’s responses; however, individual preterm-born children’s responses most resembled those observed in same-age term-born children, relative to younger children or other preterm-born children. Taken together, preterm birth does not appear to preclude broadly similar functional development in ToM brain regions by age 5 years.

## 1 Introduction

Preterm birth—birth before 37 weeks’ gestation—affects 10% of pregnancies globally (Ohuma et al., 2023) and confers increased likelihood for social and learning difficulties (Dean et al., 2021; Fenoglio et al., 2017; Johnson & Marlow, 2017; van Noort-van der Spek et al., 2012). Theory of Mind (ToM) reasoning—our ability to consider others’ mental states (e.g., beliefs, desires, emotions; Wellman, 2018)—is one aspect of social cognition that appears to be impacted by preterm birth (Jones et al., 2013; Roldán-Tapia et al., 2017; Wolfe et al., 2015). For example, young preterm-born children score lower on ToM behavioural batteries relative to term-born children (Marleau et al., 2021; Witt et al., 2018). There is also evidence for differences in social behaviours outside of the laboratory setting: children born preterm can have a harder time developing friendships (Ritchie et al., 2018) and adults born preterm are less likely to have romantic partnerships (Mendonça et al., 2019). However, the neurocognitive mechanisms by which preterm birth impacts social behaviours remain unclear.

Preterm birth may directly impact domain-specific ToM development (i.e., development of ToM-specific representations/processes) by altering babies’ earliest social experiences. For example, preterm-born babies are often cared for in Neonatal Intensive Care Units (NICUs) until they reach term-equivalent age (i.e., anywhere from 2 months to up to 4 months’ time, for infants born at 22–32 weeks). While NICUs are designed to support the health and wellbeing of newborns, the social environment of the NICU is different from the home environment. For example, skin-to-skin contact is often limited immediately after preterm birth and the number of carers preterm babies interact with is larger and more diverse than that of babies born at term, with medical and nursing professionals assuming key roles in neonatal care (Patel et al., 2017; Waddington et al., 2021). It is unclear if and how these early differences in social experience impact subsequent ToM development (Firestein et al., 2022) and worth noting that any social input experienced during this time exceeds that of age-matched babies, who are still in the womb. However, even after babies born preterm leave the hospital, their social experiences may continue to differ from those of term babies. Parents of preterm-born babies are at an increased risk of depression (Pace et al., 2016; Treyvaud et al., 2014), which could shape early social experiences by impacting parenting behaviour (Slomian et al., 2019) and, in turn, shape ToM development (Pavarini et al., 2013). For example, parental sensitivity to their infants’ internal states correlates with subsequent ToM reasoning development (Ereky-Stevens, 2008).

Alternatively, lower scores on behavioural ToM tasks among preterm-born children may reflect differences in other cognitive capacities like attention, language, and executive functions (Johnson & Marlow, 2017; Sandoval et al., 2022; van Noort-van der Spek et al., 2012). Most ToM tasks involve attending to brief stories, forming and producing (often linguistic) responses, and inhibiting prepotent responses (Hughes & Ensor, 2007; Milligan et al., 2007). As young children born preterm can experience atypical attention (Brogan et al., 2014; van de Weijer-Bergsma et al., 2008), language (Guarini et al., 2016; Vandormael et al., 2019), and executive function development (O’Meagher et al., 2017; Sandoval et al., 2022), these children, in particular, may struggle to meet the demands on these capacities inherent to most ToM behavioural tasks (Dean et al., 2021).

Neuroimaging data can provide a useful lens for understanding neurocognitive mechanisms underlying behaviour because different brain regions support different cognitive functions. A substantial body of research finds that a network of brain regions including bilateral temporoparietal junction, precuneus, and dorso-, middle-, and ventromedial prefrontal cortex is reliably recruited when adults (Carrington & Bailey, 2009; Saxe & Kanwisher, 2003) and young children (Gweon et al., 2012; Richardson et al., 2018) engage in ToM reasoning. In individual participants, this network is functionally distinct from brain regions that support language (Fedorenko et al., 2011; Paunov et al., 2022), attention (Mars et al., 2012; Scholz et al., 2009), and executive functions (Saxe, Schulz, et al., 2006). If preterm-born children experience domain-specific differences in ToM development, they should show different/delayed development and/or recruitment of these “Theory of Mind” brain regions.

There are broadly distributed differences in brain structure following preterm birth, including white matter injury or dysmaturation, cortical and deep grey matter alteration, and cerebellar abnormalities. Collectively, these are termed the encephalopathy of prematurity (Inder et al., 2023; Volpe, 2009), which is characterised by MRI as differences in structural connectivity across brain networks, including tracts in the ToM network. Two neuroimaging studies have characterised functional responses during ToM reasoning in school-aged preterm-born children. Mossad et al. (2017) reported reduced activation during belief-reasoning in 7–13-year-old preterm-born relative to term-born children who underwent magnetoencephalography (MEG); however, the regions with significantly reduced activity were not canonical ToM regions. Mossad et al. (2021) did not find differences between 8-year-old very preterm and term-born children in whole-brain random effects analyses of functional magnetic resonance imaging (fMRI) data; via MEG, they found reduced functional connectivity in children born very preterm across several regions, including ToM regions. In contrast to these neuroimaging studies, which focus on children 7 years of age and older, a vast majority of behavioural research on ToM development focuses on early childhood—that is, from infancy to 6 years of age (Knudsen & Liszkowski, 2012; Rakoczy, 2022; Wellman et al., 2001). Early childhood is a period of dramatic ToM development, as evidenced by both behavioural (e.g., Wellman & Liu, 2004) and neuroimaging (Richardson et al., 2018; Sabbagh et al., 2009) research. Given the older age-range of the children, these prior studies cannot directly speak to whether differences in ToM brain region development underlie differences in ToM behaviour in early childhood.

Here, we aimed to build on this prior research by using behavioural and neural measures to test for a direct, domain-specific effect of preterm birth on ToM development in young children. Participants were 5-year-old children in the Theirworld Edinburgh Birth Cohort—a multimodal prospective longitudinal cohort study aimed to characterise neural substrates (structural and functional) of cognitive function following preterm birth and to identify factors shaping neurocognitive development (Boardman et al., 2020, 2024). Children completed a comprehensive behavioural ToM task that measured reasoning about a range of ToM concepts (beliefs, desires, referents, moral blame assignment) via two-alternative forced choice and free response questions. Relative to prior research with preterm-born children, this task has a large number of items and is novel in its inclusion of two types of response formats. Children also underwent fMRI while viewing a short, silent movie with scenes that evoke ToM reasoning (Jacoby et al., 2016; Richardson et al., 2018). Movie-viewing fMRI experiments minimise participant motion and increase compliance, enabling children as young as 3 years of age to complete fMRI scans (Richardson et al., 2018)—and so are particularly well-suited for studying young children born preterm. Importantly, such experiments have been used to characterise development in ToM brain regions in young children (Richardson, 2019; Richardson et al., 2018). In contrast to prior neuroimaging research on ToM in children born preterm, we take a region of interest (ROI) approach. Given prior work establishing that a consistent network of brain regions is recruited during ToM reasoning (Carrington & Bailey, 2009; Gweon et al., 2012; Richardson et al., 2018; Saxe & Kanwisher, 2003), we ask: does the functional response profile in these specific brain regions in young children born preterm resemble that observed in children born at term? We extracted three measures of neural ToM development based on prior research (Richardson, 2019; Richardson et al., 2018): functional maturation (i.e., the similarity of each child’s functional response to the movie to an adult average response), response magnitude to particular movie scenes that evoke ToM reasoning, and inter-region correlations (i.e., functional connectivity) within the ToM network and across the ToM network and the Pain Matrix—a network involved in reasoning about bodily sensations that becomes increasingly functionally distinct from the ToM network with age (Bruneau et al., 2012; Richardson, 2019; Richardson et al., 2018). We also conducted inter-subject correlation analyses to test whether children born preterm (1) show “distinct” ToM neural responses to the movie (i.e., are more similar to one another than to term-born children, in terms of their response to the movie), (2) show more variable ToM neural responses to the movie, relative to children born at term, and (3) show neural ToM responses that resemble those in younger term-born children (3- to 4-year-old children) rather than age-matched term-born children, suggesting delayed ToM development.

## 2 Methods

### 2.1 Participants

Participants were recruited into the Theirworld Edinburgh Birth Cohort (TEBC), a prospective longitudinal cohort study designed to investigate the causes and consequences of preterm birth on brain development and childhood outcomes (Boardman et al., 2020, 2024). Infants born at <32 weeks’ gestation and term comparators were recruited from the Royal Infirmary of Edinburgh, NHS Lothian, UK between November 2016 and September 2021. Infants with major congenital malformation, chromosomal abnormality, congenital infection, major parenchymal brain injury apparent on routine ultrasound and those with contraindications to MRI were excluded from the cohort, such that the TEBC represents the majority of survivors of modern neonatal intensive care. The conventional neonatal MRI imaging is reported in Sullivan et al. (2024); no infants had major injurious parenchymal lesions that might be expected to impact functional networks.

Data collection for the 5-year follow-up began in February 2022 and is ongoing. For the current study, 52 preterm-born children and 58 term-born children contributed behavioural ToM data. Thirty preterm-born children (including two children who did not complete the behavioural assessment and 46 term-born children) contributed usable fMRI data (i.e., fewer than 1/3 of the acquired volumes were flagged as motion artifact timepoints, see fMRI data analyses for further details and Table 1 for a summary of demographic information). This sample size is in line with prior research on neural ToM development in term-born young children (e.g., 65 3–5-year-old children in Richardson et al., 2018) and prior neuroimaging research with older children born preterm (e.g., 25 very preterm and 35 full-term children in Mossad et al., 2021). One additional child born preterm completed the fMRI scan but was excluded from analyses due to excessive motion (see fMRI data analyses). Anatomical MRI images were reviewed by a paediatric radiologist; incidental findings were not used as an exclusion criterion.

**Table 1.** Sample characteristics at birth and testing.

Sample	Preterm/Term	N	Mean (range) GA in weeks	Mean (range) test age in years	Mean (SE) receptive language	Mean (SE) total behavioural ToM booklet performance
TEBC all	Preterm	52	29.0 (24.0–31.9)	5.20 (5.06–5.86)	34.0 (3.35)	0.490 (0.022)
	Term	58	39.7 (36.4–42.1)	5.19 (5.02–5.65)	48.0 (3.53)	0.615 (0.025)
TEBC fMRI	Preterm	30	29.1 (24.7–31.9)	5.20 (5.05–5.86)	35.3 (4.85)	0.506 (0.032)
	Term	46	39.7 (36.4–42.1)	5.18 (5.02–5.65)	51.1 (3.84)	0.638 (0.027)
Open Data 3–4 and 5-year olds	Term	27	-	4.07 (3.53–4.86)	-	0.574 (0.031)
	Term	30	-	5.51 (5.01–5.99)	-	0.719 (0.030)

Characteristics for the samples included across analyses. “Sample” indicates which sample participants belonged to, where “TEBC all” comprises all children with behavioural ToM data, “TEBC fMRI” comprises all children who contributed usable fMRI data, and “Open Data” comprises the 3- to 5-year-old children from the open dataset used for inter-subject correlation analyses. “Preterm/ Term” indicates birth status for the number of children (“N”) within each sample. For gestational age (GA) and test age means and ranges are shown. Means and standard error (SE) are shown for Receptive Language percentile rank and ToM proportion correct.

Parents provided written informed consent prior to participation. The study protocol was approved by the National Research Ethics Service, South East Scotland Research Ethics Committee (16/SS/0154) and the NHS Research and Development (2016/0255) on the 5th October 2016.

To estimate functional maturity in ToM brain regions and test the hypothesis that preterm-born children show delayed ToM development (see intersubject correlation analysis within Regions of Interest analyses section), we leveraged an open dataset that included 33 adults and 57 3.5–5-year-old children (see Table 1) who completed the same movie-viewing fMRI experiment. Data were acquired at the Athinoula A. Martinos Imaging Center at MIT (https://openneuro.org/datasets/ds000228, Richardson et al., 2018). Eight additional children were excluded from analyses (*n* = 1 due to preprocessing failure (issues with the a priori defacing procedure); *n* = 3 for excessive motion (using the same thresholds as the TEBC cohort; see fMRI data analyses), *n* = 4 for being born preterm).

### 2.2 Measures

#### 2.2.1 Behavioural theory of mind booklet task

Children completed a publicly available ToM booklet task (Sotomayor-Enriquez et al., 2024), which involved listening to an experimenter tell a story and answering 25 two-alternative forced choice (2AFC) and 21 free response questions about the characters’ mental states. Children completed two control items to demonstrate task understanding. The task assesses reasoning about ToM concepts of varying difficulty (e.g., similar/diverse desires and knowledge access (“easy”), false beliefs, moral blameworthiness and reference understanding (“hard”)) and captures variability in ToM in childhood (Gweon et al., 2012; Richardson et al., 2018). Administration was video recorded, and responses were transcribed and coded offline. Our primary measure was the proportion of correct answers to all test questions (*n* = 46). To facilitate comparisons between our study and prior research, which mostly focuses on children’s performance during false belief tasks, we repeated analyses with false belief items only (n = 14). We also conducted planned exploratory analyses examining differences by response type (i.e., 2AFC vs. free response items).

#### 2.2.2 Behavioural “Partly Cloudy” task

During the fMRI scan, children viewed Disney Pixar’s “Partly Cloudy” (Reher & Sohn, 2009) in full. After the scan, children were asked questions assessing attention to and comprehension of the movie as well as movie-specific ToM reasoning. Children answered four attention/comprehension questions (three 2AFC and one free response), which required children to remember and report details of the movie (e.g., “Why is Peck missing feathers? What happened to him?”) and 12 ToM questions (four 2AFC and eight free response), which prompted reasoning about the characters’ mental states (e.g., “How does Gus feel when Peck leaves?”). Children were shown 4 brief clips from “Partly Cloudy” during this task to contextualise the questions. Task materials are publicly available (https://osf.io/w9et8/). Responses were video recorded and transcribed/coded offline. We calculated the proportion of items answered correctly for attention/comprehension and ToM reasoning items, separately. The ToM-specific measure correlated with performance on the ToM behavioural battery when controlling for age, but not when additionally controlling for language (see Supplementary Material Supplement S1 and Fig. S1).

#### 2.2.3 Other cognitive measures and covariates

**Fig. 1.**
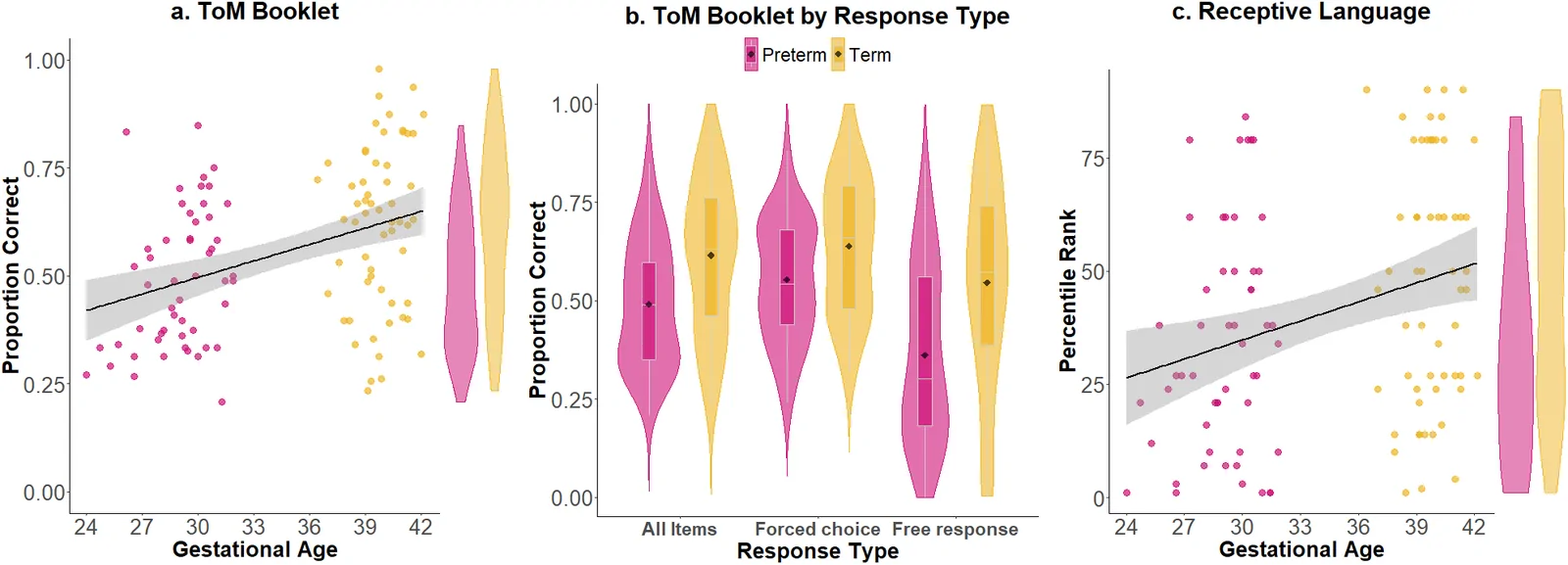
ToM booklet task and receptive language. (a) Proportion correct on all 46 ToM booklet test questions. (b) Proportion correct on ToM test questions by response type (left: all items, middle: two-alternative forced choice items, right: free response items). (c) Receptive language percentile rank score (y-axis) by GA (in weeks, x-axis). In all plots, preterm-born children (*n* = 52) are shown in magenta and term-born children (*n* = 58) are shown in yellow. In violin plots (b), black diamonds indicate group means. The horizontal line in the boxplot shows the median, the lower and upper hinges of the boxes correspond to the first and third quartiles, with the upper whisker extending to the largest value no further than 1.5 * inter-quartile range (IQR) from the hinge and the lower whisker extending from the lower hinge to the smallest value at most 1.5 * IQR of the hinge.

To account for other factors that impact ToM development and performance on ToM tasks, we tested for effects of gestational age (GA) on measures of language, executive functions, and attention, and examined differences in children’s number of siblings by GA. For further details on the selection of covariates in this study, see Supplement S2. Briefly, only language, measured as the standardised (percentile rank) “receptive language” subscale score from the Mullen Scales of Early Learning (Mullen, 1995), correlated with GA (see Fig. 1c and Supplement S2)—therefore, language was included as a covariate in analyses testing for an effect of GA on ToM behavioural performance.

### 2.3 MRI data acquisition

Data were acquired at the Edinburgh Imaging Facility (Royal Infirmary of Edinburgh, University of Edinburgh; CRF number E161723). Whole-brain MRI data were acquired on a Siemens Magnetom Prisma 3-Tesla (3T) scanner (Siemens Healthcare, Erlangen, Germany) using a 32-channel phased-array head coil. 3D sagittal T1 MPRAGE structural images were acquired first (TR = 2500 ms, TE = 4.69 ms, TI = 1180 ms, FA = 7°, isotropic voxel size = 1 mm, FOV = 256 mm x 256 mm x 192 mm), followed by a 3D T2-weighted (T2w) sampling perfection with application-optimized contrasts by using a flip angle evolution (SPACE) structural scan (isotropic voxel size = 0.9 mm, TE = 407 ms, TR = 3200 ms). Trained radiographers viewed structural scans upon acquisition to determine need for repeat scans. Functional MRI data were acquired with a gradient-echo EPI sequence in 45 interleaved slices and 324 volumes per run (voxel size = 2.3 mm isotropic, resampled to 2 mm during preprocessing, TR = 1 seconds, TE = 30 ms, flip angle = 60°, slice gap = 20%, parallel imaging acceleration factor 2/3 in phase-encoding/slice directions). A *B*_0_ fieldmap was acquired to facilitate geometric distortion correction of functional MR images. The scanning protocol includes additional structural, diffusion, and functional sequences not analysed here (total scan time = 50 minutes; for details, see Boardman et al., 2024). For details on how children were prepared to take part in the scan, see Supplement S3.

#### 2.3.1 Functional MRI paradigm

Participants viewed a silent version of the 5.6-minute animated movie “Partly Cloudy” (Reher & Sohn, 2009; see https://www.pixar.com/partly-cloudy#partly-cloudy-1 for a plot description), preceded by 10 seconds of rest, while undergoing fMRI. This movie has proven useful for characterising development in multiple cortical networks, including the ToM network and “Pain matrix” (a network recruited to reason about bodily sensations, including pain; Bruneau et al., 2012, 2015; Richardson et al., 2018). Briefly, the movie is about two characters: a stork (Peck) and a cloud (Gus). Peck and Gus are friends; they also work together: Gus makes animal babies for Peck to take down to Earth. Unfortunately, Gus exclusively makes babies that hurt Peck (e.g., a crocodile that bites him, a hedgehog that pricks him). This causes some challenges for their friendship. Throughout the movie, Peck gets injured by these animals while nearby clouds make cute animal babies (e.g., kittens and puppies) for other storks to deliver. All but 2 preterm- and 5 term-born children had not previously seen the movie.

Children viewed “Partly Cloudy” twice, across two runs. We analysed the first run for all but one term-born child, whose first run was excluded for excessive motion (see fMRI data analyses), to match the number of analysed runs across participants and because there are differences in ToM brain region responses across repeated movie-viewings (Richardson & Saxe, 2020).

### 2.4 fMRI data analyses

The fMRI analysis pipeline (developed by M. Thye) is publicly available (https://github.com/hrichardsonlab/fmri-analysis). Raw TEBC DICOM images were converted to NIfTI format and organised according to BIDS specification (Gorgolewski et al., 2017; https://bids.neuroimaging.io/). All data (TEBC and open data) were preprocessed using fMRIPrep 24.0.0 (Esteban et al., 2019, 2020), which is based on Nipype (Gorgolewski et al., 2018). Briefly, the anatomical (T1w) image was skull stripped, segmented into cerebral spinal fluid, white matter, and gray matter, and normalised to Montreal Neurological Institute (MNI) standard space (MNI152NLin2009cAsym). For each functional run, a reference image and its skull-stripped version was created. Translational and rotational motion parameters were estimated per volume against the functional reference image. Functional reference images were co-registered to the T1w reference image and resampled to standard MNI space. FMRIprep computes several confound regressors, including framewise displacement (FD), DVARS and aCompCor regressors (Behzadi et al., 2007). Visual inspection of fMRIprep outputs (segmentation, coregistration, normalisation, and distortion correction) ensured that there were no preprocessing errors (i.e., there were no sharp cut-offs in masks; anatomical data and functional data were aligned; normalisation did not introduce errors). See Supplement S4 for the full description of preprocessing steps generated by fMRIprep. Data were subsequently smoothed with a 5 mm FWHM Gaussian kernel.

Motion artifact timepoints were defined as those with (1) framewise displacement (FD) >1 mm, (2) standardised DVARS > 1.5, or (3) using the following thresholds within rapidart, the nipype implementation of the ART toolbox: more than 1 mm composite motion relative to the previous volume or a fluctuation in global signal exceeding three standard deviations from the mean global signal. If one-third or more of the volumes within the functional run were identified as artifact timepoints, the run was excluded from further analyses (*n* = 1 child born preterm, *n* = 3 children from MIT dataset). Mean FD was calculated across the full run, including motion artifact timepoints. Overall, mean FD was small (*M*(*SD*) for preterm group: 0.33(0.27), for term group: 0.29(0.19)), and few timepoints were identified as motion artifacts (*M*(*SD*) for preterm group: 17.8(24.1), for term group: 11(12.1); see Supplementary Fig. S2). However, there was a small effect such that across the full sample, children born at a higher GAs moved less (Pearson correlation between mean FD and GA across the full sample: *r*(74) = -0.08, Cohen’s *d *= -0.16) and a medium effect such that children born at a higher GAs had fewer motion artifact timepoints (Pearson correlation between number of artifact timepoints and GA across the full sample: *r*(74) = -0.18, Cohen’s *d *= -0.37). Given this, we repeated analyses in motion-matched subsamples of preterm (*n* = 27) and term-born (*n* = 27) children (mean FD: *r*(52) = 0.05, Cohen’s *d* = 0.1, negligible effect; *M*(*SD*) for preterm group: 0.26(0.13), for term group: 0.27(0.15); artifact timepoints: *r*(52) = -0.008, Cohen’s *d *= -0.02, negligible effect; *M*(*SD*) for preterm group: 10.7(11), for term group: 11.4(10.6)). Mean FD (“motion”) was included as a covariate in all linear regression analyses testing for between-subject effects in neural responses.

All analysis details are described below; see also see Supplementary Table S1 for an overview of analyses.

#### 2.4.1 First-level modelling

We used a general linear model to analyse voxel-wise BOLD data from each participant as a function of event type. “Mind” (*n* = 7; total duration: 68 seconds, range: 4–16 seconds) and “Body” (*n* = 9; total duration: 52 seconds, range: 2–12 seconds) scenes (“events”) during “Partly Cloudy” were defined in a prior study with the MIT adults (*n* = 33) using reverse correlation analyses applied to network-average response timecourses extracted from the ToM network and Pain Matrix (a functional network recruited to reason about bodily sensations, including pain), respectively (Richardson et al., 2018). Reverse correlation analyses are a data-driven analysis that can reveal scenes during movie-viewing that evoke reliable responses across participants (Hasson et al., 2004). We took the onsets and durations of mind and body events identified by the prior study (Richardson et al., 2018) and used them to create “Mind” and “Body” boxcar regressors, which were convolved with a canonical hemodynamic response function (HRF). Nuisance regressors for artifact timepoints and five aCompCor regressors were included in the model. Data were high-pass filtered (0.01 Hz).

#### 2.4.2 Whole-brain random effects analyses

Second-level random effects analyses were conducted to investigate activation to the Mind>Body contrast at the group-level (i.e., in preterm-born and, separately, term-born children) and to test for an effect of GA on the Mind>Body contrast. For all random effects analyses, we used FSL’s RANDOMISE to conduct nonparametric tests (5000 permutations, threshold α = 0.05) and accounted for multiple comparisons using threshold-free cluster enhancement (TFCE).

#### 2.4.3 Regions of interest (ROI) analyses

Given prior work establishing that a consistent network of brain regions is recruited during ToM reasoning (Carrington & Bailey, 2009; Gweon et al., 2012; Richardson et al., 2018; Saxe & Kanwisher, 2003), we asked: does the functional response profile in these specific brain regions in young preterm-born children resemble that observed in term-born children? Following previous research (Richardson, 2019; Richardson et al., 2018), we extracted timecourses from six group-defined ToM brain regions: right and left temporoparietal junction (R/LTPJ), precuneus (PC), and dorso-/middle-/ventromedial prefrontal cortex (D/M/VMPFC). In line with prior research (Richardson, 2019; Richardson et al., 2018), for inter-region correlation analyses (described below), we also extracted timecourses from seven “Pain Matrix” brain regions: anterior cingulate cortex (ACC) and bilateral insula, secondary sensory motor cortex, and medial frontal gyrus. ROIs were previously defined in an independent sample of adults (*n* = 20; see Richardson et al., 2018) and are publicly available (https://openneuro.org/datasets/ds000228).

We extracted preprocessed timecourses from each voxel per group-defined ROI. Motion artifact timepoint volumes were interpolated with cubic spline interpolation and timecourses were high-pass filtered (0.01 Hz). Motion artifact timepoints were censored and the top five aCompCor components were regressed out. Timecourses were averaged across voxels within each ROI and z-normalised, resulting in one timecourse per ROI and participant. To account for differences in repetition time across datasets (open data: TR = 2 seconds; TEBC: TR = 1 second), we interpolated individual timecourses for open data participants, that is, the average value was inserted between every two original data timepoints (see Supplementary Fig. S3 for a visualisation).

Following prior research (Richardson et al., 2018), we extracted six measures of ToM brain region development: functional maturity, response magnitude to three “Mind” events, and within-ToM and across-ToM-Pain network inter-region correlations.

Functional maturity is the extent to which each child’s timecourse correlates with an adult average timecourse (Cantlon & Li, 2013; Richardson et al., 2018). For each ROI, we averaged response timecourses across adults to obtain an adult average timecourse and calculated the z-scored Pearson correlation value between that average timecourse and each TEBC child’s timecourse. Correlation values are z-scored because r-values are not normally distributed (see Cantlon & Li (2013) and Wilson et al. (2008) for similar approach). Like any inter-subject correlation measure (Nastase et al., 2019), these correlation values will capture commonalities in response timecourses due to processing within the ROIs, which can reflect the representations/computations in those brain regions and/or downstream consequences of common responses elsewhere in the brain. The logic of intersubject correlations is to determine the extent to which neural responses are stimulus driven (Nastase et al., 2019); the logic of functional maturation indices is to measure change with age in stimulus-driven responses (i.e., similarity to a “mature” response; Cantlon & Li, 2013; Richardson et al., 2018). Here, we test for effects of GA (in addition to age) on this metric.

The response magnitude to three “Mind” events was extracted per TEBC child and ROI. Events were originally defined using reverse correlation analyses in adult data (defined as events longer than 4 seconds that evoked positive responses; Richardson et al., 2018). We selected these three events for analyses because prior research found that response magnitude to these events correlated with age (event 1 (originally labelled T02 in Richardson et al., 2018), event 2 (T01)) or ToM behavioural score, controlling for age (event 3 (T04)) in 3-to-12-year-old children (Richardson et al., 2018; see Supplementary Table S2 for timepoints and descriptions of the content of the events). Events were originally labelled according to the average peak response magnitude in adults (with T01 reflecting the largest response magnitude across events). To align events identified by reverse correlation analyses with scenes in the movie, the previous study assumed a 4 seconds hemodynamic lag between the onset of a particular scene and the neural ToM response in adults (Richardson et al., 2018). Briefly, in event 1 (T02), Gus catches Peck gazing at the other, happy clouds; in event 2 (T01), Peck (the stork) flies away from Gus (the cloud) to a different cloud; in event 3 (T04), Peck dons protective gear, showing Gus that he had not abandoned him but rather only left to acquire gear to enable him to continue to work with Gus. All three Mind events contain facial expressions of emotions and encourage the viewer to make inferences about the characters’ mental states. To account for the difference in TR (1 TR = 2 seconds in Richardson et al., 2018), we extracted the average response magnitude from the two timepoints (1 second each) corresponding to the peak timepoint identified by Richardson et al. (2018) for each event.

In inter-region correlation analyses, we calculated, for each child, the z-scored Pearson correlation between each ROI’s timecourse and every other ROI’s timecourse. Within-ToM network correlations were the average correlation for each ToM ROI to every other ToM ROI; within-Pain network correlations were the average correlation for each Pain Matrix ROI to every other Pain Matrix ROI; across-ToM-Pain network correlations were the average correlation from each ToM ROI to each Pain Matrix ROI. Inter-region correlations measure functional coherence of brain regions within a network and functional distinctions across networks (Blank et al., 2014; Hasson et al., 2004). The Pain Matrix has been used as a comparator network in prior research (Richardson et al., 2018), given its role in processing internal *bodily* states (e.g., hunger, fatigue, pain; (Bruneau et al., 2012, 2015; Spunt et al., 2016), rather than internal *mental* states (beliefs, desires, emotions) and relevance to the content of “Partly Cloudy”. This prior research found increasing within-ToM correlations and decreasing across-ToM-Pain network correlations with age (Richardson et al., 2018).

We used generalised linear fixed and mixed-effects regression models to test for effects of GA on these neural measures, controlling for test age and motion. All statistical analyses used GA as a continuous variable. For our main analyses data were modelled across the full GA range (i.e., across both term- and preterm-born children), but we additionally completed analyses within the preterm sample only, to assess dose-dependency of any effects of GA among these children. For ROI-specific measures (functional maturity, response magnitude), we used mixed-effects linear regressions with ROI as a fixed effect and subject as random effect. We tested for GA-by-ROI interactions, removing non-significant interactions from final regressions (see Supplement S5). We used linear regressions to test for effects in RTPJ, separately, given evidence that responses in this region correlate with ToM development (Gweon et al., 2012; Richardson, Gweon, et al., 2020; Richardson, Koster-Hale, et al., 2020) and used it as the reference ROI (i.e., the ROI against which all other ROIs in the model are referenced) in regressions that included all ToM ROIs. We applied Bonferroni corrections for multiple comparisons (six neural ToM measures, α = 0.008).

Finally, we conducted intersubject correlation analyses to quantify similarity in ToM brain region responses to the movie across children (Hasson et al., 2004). For each child we calculated an average ToM network timecourse. For this average ToM timecourse and for RTPJ specifically, we correlated each child’s timecourse to every other child in the TEBC sample and in the 3-to-5-year-old (term-born) open dataset. We used paired t-tests comparing z-scored Pearson correlations to test three hypotheses: (1) To test if preterm-born children showed distinct ToM neural responses to the movie, we tested if within-preterm-group correlation values were higher than across-preterm-term-group correlation values (one-tailed); we also conducted this analysis for term-born TEBC children (i.e., compared within-term-group to across-preterm-term-group correlation values). (2) To test if neural ToM responses in preterm-born children appeared developmentally delayed, we tested if their responses were more correlated with 3.5–4-year-old term-born children or with 5-year-old term-born children (two-tailed test; all term-born children were from the open MIT dataset; see Supplementary Fig. S4 for analyses of site effects). For this analysis, we accounted for differences in repetition time—and therefore response timecourse length—across datasets (open data: TR = 2 seconds; TEBC: TR = 1 second), by interpolating individual timecourses for open data participants only, that is, the average value was inserted between every two original data timepoints, prior to calculating inter-subject correlations. We applied this approach because it introduced minimal changes to the response timecourses from the open dataset (and no changes to response timecourses from the TEBC children) and is in line with commonly used preprocessing strategies (Carp, 2013; Hallquist et al., 2013; Power et al., 2012; see Supplementary Fig. S3). Importantly, any differences in response timecourses across sites would be matched across preterm- and term-born TEBC children (i.e., differences in ISC values by GA cannot be explained by site effects/this interpolation approach). (3) To test if preterm-born children showed more variable ToM neural responses, we tested if within-group correlations were lower among preterm-born children, relative to (TEBC) term-born children (one-tailed).

All statistical analyses were carried out in R (v4.5.1).

### 2.5 Open science practices

Analyses were pre-registered to the Open Science Framework (behavioural: https://osf.io/93x7e/overview; fMRI: https://osf.io/rv76a/overview). Non-preregistered analyses are indicated. Deviations from the pre-registered analyses were minimal and described in Supplement S5. The fMRI analysis pipeline (https://github.com/hrichardsonlab/fmri-analysis) and code (https://osf.io/xtyu8/overview) are publicly available, data for reproducing analyses will be available through a data usage agreement process.

## 3 Results

### 3.1 Behavioural results

#### 3.1.1 Theory of mind booklet (outside of the scanner)

Across the full sample of preterm- (*n* = 52) and term-born (*n* = 58) children, GA had a positive effect on behavioural ToM booklet task performance, controlling for test age (effect of GA: β = 0.07, *t* = 4.28, *p* = 4.08e-05; test age: β = 0.002, *t* = 0.1, *p =* 0.924; *M*(*SE*) proportion correct in preterm-born children: 0.49(0.02), term-born children: 0.62(0.03); Fig. 1a). The effect of GA on ToM performance remained significant when controlling for receptive language (effect of GA: β *=* 0.04, *t =* 2.86, *p =* 0.005; receptive language: β *=* 0.09, *t =* 5.53, *p =* 2.32e-07; test age: β *=* 0.02, *t =* 1.36, *p =* 0.176). The same pattern of results was observed for the 14-item false belief composite score (see Supplement S6); GA impacted performance on ToM items regardless of conceptual difficulty (Supplementary Fig. S5).

Additional analysis of the positive effect of GA on ToM performance among only the preterm-born children showed that this effect was “dose-dependent”—that is, within the preterm-born group (*n* = 52), those born at higher GA also performed better on the ToM task, controlling for test age and receptive language (β = 0.05, *t* = 2.29, *p* = 0.027).

In a (non-preregistered) mixed-effects logistic regression across the full sample (term- and preterm-born children) simultaneously testing for effects of GA, response type (2AFC vs. free response), and ToM concept difficulty (easy vs. false belief vs. hard) there was a significant GA-by-response type interaction such that the positive effect of GA was stronger for free response items (β = 0.27, *z* = 3.97, *p =* 7.25e-05; Fig. 1b). There were also main effects of receptive language, response format (indicating free response items were more difficult than 2AFC items), and ToM concept difficulty, as well as a response format-by-difficulty interaction effect; main effects of GA and test age were not statistically significant (see Supplementary Table S3 for full statistics and Fig. 1b and Supplementary Fig. S5).

Full statistical results for the same analyses conducted in the subset of children who contributed fMRI data (preterm: *n* = 28, term: *n* = 46 are reported in Supplement S7 and Supplementary Table S4. Briefly, the patterns of results observed in this subset of children were the same, with the exception that in the linear regression on the proportion correct dependent variable, the effect of gestational age was not “dose-dependent”—that is, significant among preterm-born children alone. The pattern of results of the mixed-effects regression analyses was the same.

#### 3.1.2 “Partly Cloudy” movie understanding (outside of the scanner)

We did not observe a statistically significant effect of GA on movie-specific ToM reasoning performance in the full sample (effect of GA: β = 0.04, *t* = 1.86, *p* = 0.067; test age: β = 0.02, *t* = 1.06, *p* = 0.294; M(*SE*) in preterm group: 0.57(0.02); in term group: 0.63(0.03)). We also did not observe a statistically significant effect of GA on attention/comprehension item performance, controlling for test age (effect of GA: β = 0.07, *t* = 1.96, *p* = 0.055; test age: β = 0.003, *t* = 0.09, *p* = 0.933; *M*(*SE*) preterm group: 0.39(0.05); term group: 0.52(0.04)).

### 3.2 Movie-viewing fMRI results

#### 3.2.1 Whole-brain random effects analysis

**Fig. 2.**
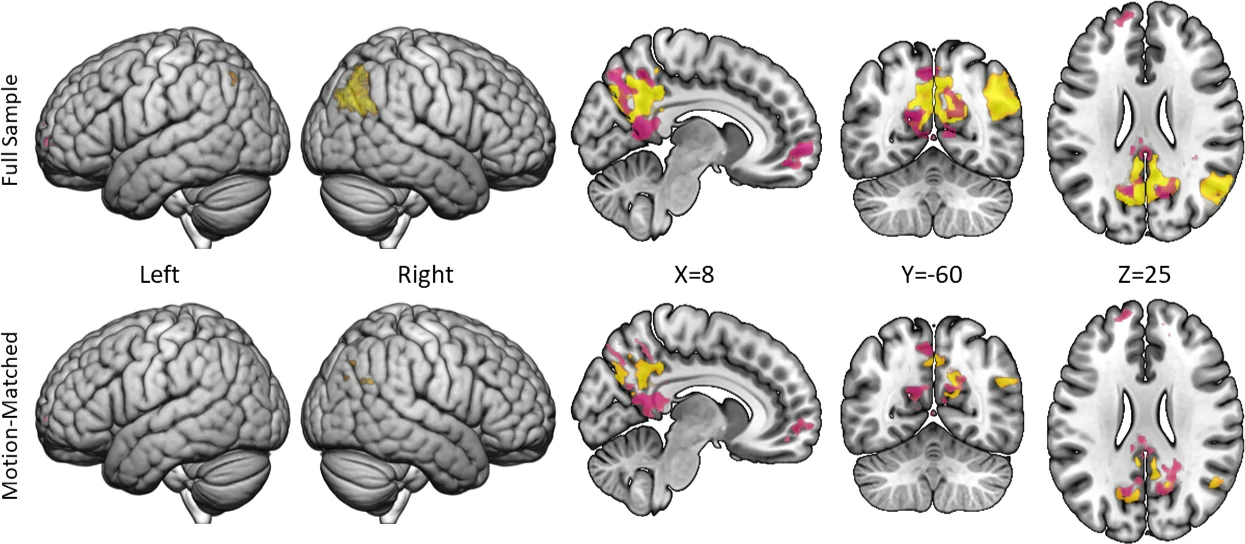
Whole-brain random effects analysis of the Mind>Body events contrast. The top panel shows results in the full sample (preterm *n* = 30 (magenta), term *n* = 46 (yellow)) with a TFCE corrected threshold of *p* = 0.05, the bottom panel shows results in the motion-matched subsample (*n* = 27 for both groups) with a more lenient threshold of *p* = 0.1 (TFCE corrected).

Both groups (preterm *n* = 30; term *n* = 46) showed approximately the same network of regions previously used in adults to define Mind and Body events (TPJ, PC, and MPFC; Richardson et al. (2018); *p* = 0.05, Fig. 2, top panel). Similar results were observed in children tightly matched for motion and sample size (visualised at a more lenient threshold of *p* = 0.1, Fig. 2, bottom panel). In the full sample, there were no clusters in which activation to the Mind>Body contrast varied significantly as a function of GA.

#### 3.2.2 Regions of interest analyses

**Fig. 3.**
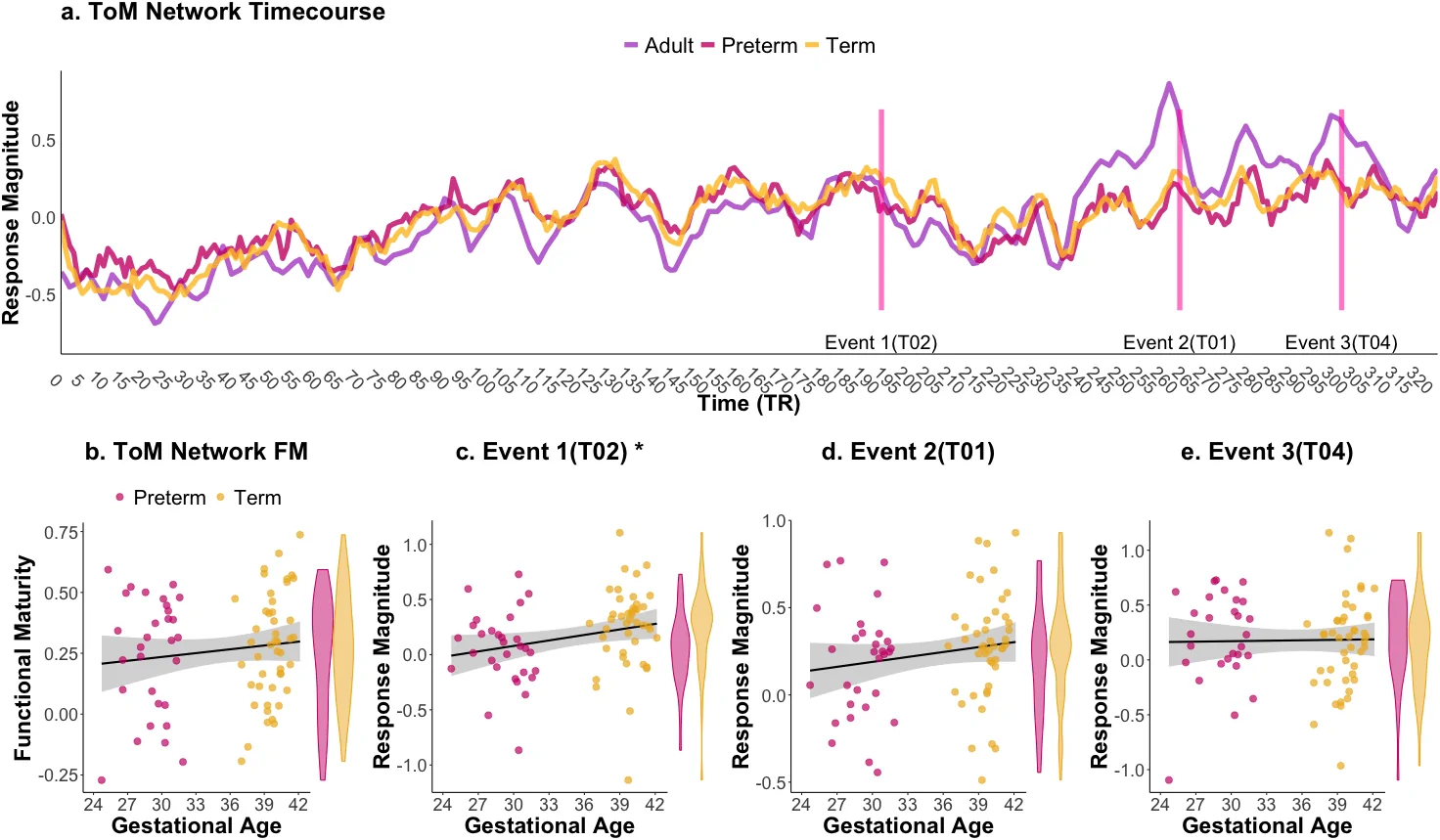
ToM regions of interest measures by gestational age. Top panel plot (a) shows the average response magnitude (y-axis) across time (in seconds, x-axis) in the ToM network, per group (preterm in magenta, *n* = 30; term in yellow, *n* = 46; adults in purple, *n* = 33). Vertical pink lines indicate “Mind” movie events (interrogated in response magnitude analyses). The T0# naming convention of the “Mind” events follows prior research (Richardson et al., 2018), reflecting order of response magnitude in adults. Bottom panel scatter plots show functional maturity (b) and response magnitude to mind events (plots c-e) by gestational age (x-axes) for the ToM network. Note that plots show the average measure across ToM brain regions for illustrative purposes, while statistical analyses used mixed-effects regressions to include data from all ToM ROIs. Violin plots to the right of each scatterplot illustrate the distribution of values by group. Preterm-born children (*n* = 30) are shown in magenta, and term-born children (*n* = 46) are shown in yellow.

On average, response timecourses to “Partly Cloudy” in ToM regions looked qualitatively similar across preterm- and term-born children (as shown in Fig. 3a). For context, response timecourses from both groups positively correlated with the adult average timecourse (*M*(SE) z-scored correlation in ToM network—preterm: 0.24(0.03), term: 0.28(0.03); RTPJ—preterm: 0.28(0.06), term: 0.39(0.06); Fig. 3b). In the full sample (preterm: *n* = 30, term: *n* = 46), the effect of GA on functional maturity was not statistically significant; that is, the magnitude of these correlations was not statistically associated with GA. Similarly, we did not observe a statistical association between the response magnitude to two of three “Mind” events (Table 2) and GA.

**Table 2.** Statistical results for ROI analyses.

Neural measure	Predictors	Full sample			Motion matched sample		
Functional Maturity							
FM: ToM Network	GA	β = 0.03	*t = *1.23	*p *= 0.222	β = 0.04	*t = *1.23	*p = *0.226
	Age	β = 0.05	*t = *1.85	*p = *0.068	β = 0.03	*t = *0.87	*p = *0.389
	Motion	β = -0.04	*t = *-1.72	*p = *0.089	β = -0.04	*t = *-1.24	*p = *0.222
FM: RTPJ	GA	β = 0.07	*t = *1.28	*p = *0.204	β = 0.12	*t = *1.99	*p = *0.052
	Age	β = 0.11	*t = *2.07	*p = *0.042	β = 0.11	*t = *1.88	*p = *0.066
	Motion	β = -0.02	*t = *-0.43	*p = *0.67	β = 0.03	*t = *0.55	*p = *0.586
Response Magnitude							
RM event 1 (T02): ToM Network	GA	***β = *** **0.20**	***t = *** **3.19**	***p = *** **0.002**	***β = *** **0.21**	***t = *** **2.79**	***p = *** **0.006**
	Age	β = 0.05	*t = *1.14	*p = *0.259	β = 0.06	*t = *1.09	*p = *0.282
	Motion	β = -0.01	*t = *-0.27	*p = *0.791	β = 0.05	*t = *1.00	*p = *0.321
RM event 2 (T01): ToM Network	GA	β = 0.06	*t = *1.82	*p = *0.072	β = 0.08	*t = *1.91	*p = *0.062
	Age	β = 0.09	*t = *2.60	*p = *0.011	β = 0.07	*t = *1.58	*p = *0.120
	Motion	β = 0.01	*t = *0.30	*p = *0.763	β = -0.06	*t = *-1.38	*p = *0.175
RM event 3 (T04): ToM Network	GA	β = 0.02	*t = *0.41	*p = *0.684	β = 0.03	*t = *0.50	*p = *0.623
	Age	β = 0.07	*t = *1.49	*p = *0.141	β = 0.06	*t = *0.95	*p = *0.348
	Motion	β = -0.02	*t = *-0.30	*p = *0.766	β = -0.01	*t = *-0.18	*p = *0.857
RM event 1 (T02): RTPJ	GA	***β = *** **0.22**	***t = *** **3.14**	***p = *** **0.002**	***β = *** **0.23**	***t = *** **2.87**	***p = *** **0.006**
	Age	β = 0.12	*t = *1.76	*p = *0.082	β = 0.12	*t = *1.53	*p = *0.132
	Motion	β = -0.01	*t = *-0.09	*p = *0.926	β = 0.09	*t = *1.16	*p = *0.254
RM event 2 (T01): RTPJ	GA	β = 0.07	*t = *1.16	*p = *0.249	β = 0.08	*t = *1.19	*p = *0.239
	Age	β = 0.15	*t = *2.51	*p = *0.014	β = 0.11	*t = *1.66	*p = *0.104
	Motion	β = 0.02	*t = *0.29	*p = *0.772	β = -0.07	*t = *-1.11	*p = *0.274
RM event 3 (T04): RTPJ	GA	β = 0.04	*t = *0.40	*p = *0.691	β = 0.09	*t = *1.01	*p = *0.317
	Age	β = 0.04	*t = *0.52	*p = *0.604	β = 0.06	*t = *0.68	*p = *0.497
	Motion	β = 0.11	*t = *1.09	*p = *0.282	β = 0.14	*t = *1.60	*p = *0.116
Inter-Region Correlation							
IRC: Within ToM	GA	β = 0.05	*t = *2.11	*p = *0.038	β = 0.04	*t = *1.44	*p = *0.157
	Age	β = 0.00	*t = *0.17	*p = *0.868	β = -0.02	*t = *-0.76	*p = *0.450
	Motion	β = 0.01	*t = *0.69	*p = *0.492	β = 0.00	*t = *0.17	*p = *0.863
IRC: Within Pain (Not preregistered)	GA	β = -0.02	*t = *-1.12	*p = *0.268	β = -0.02	*t = *-1.46	*p = *0.152
	Age	β = -0.02	*t = *-1.70	*p = *0.093	β = -0.03	*t = *-1.74	*p = *0.089
	Motion	β = -0.04	*t = *-2.56	*p = *0.013	*β = *-0.04	*t = *-2.58	*p = *0.013
IRC: Across ToM-Pain	GA	β = 0.01	*t = *0.82	*p = *0.413	β = 0.00	*t = *0.08	*p = *0.939
	Age	β = -0.01	*t = *-0.76	*p = *0.451	β = -0.01	*t = *-0.92	*p = *0.363
	Motion	β = 0.02	*t *= 1.45	*p = *0.150	β = 0.00	*t = *0.25	*p = *0.807
IRC: Within ToM - Across ToM-Pain (Not preregistered)	GA	β = 0.04	*t *= 1.94	*p = *0.056	β = 0.04	*t = *1.70	*p = *0.096
	Age	β = 0.01	*t *= 0.65	*p = *0.519	β = -0.01	*t = *-0.35	*p = *0.728
	Motion	β = 0.00	*t *= -0.08	*p = *0.939	β = 0.00	*t = *0.06	*p = *0.955

Statistical results for the analyses of functional maturity, response magnitude to “Mind” events for the full ToM network and RTPJ, separately, and inter-region correlations, in the full sample (*n* = 30 preterm, *n* = 46 term-born children) and in the motion matched sub-sample (*n* = 27 children per group). Statistics include standardised betas (β), t-values (*t*), and p-values (*p*) for each predictor in the linear mixed model regression. *P*-values < 0.05 are shown in bold for effects of GA.

Children born at a higher GA had larger responses during one “Mind” event (event 1 (T02); see Table 2); however, in the average timecourses (Fig. 3), the response timecourse among preterm-born children to event 1 (T02) appeared to peak earlier than the average timecourse among term-born children—resembling the average response timecourse of adults. GA-by-ROI interaction effects indicated a stronger effect of GA on the response magnitude of RTPJ, relative to DMPFC and MMPFC (Supplementary Table S5). These results were significant in the motion-matched subsample (preterm: *n *= 27, term: *n* = 27) and survived correction for multiple comparisons (α = 0.008, Bonferroni adjusted threshold correcting for six neural measures). Post-hoc, non-preregistered analyses on the full sample confirmed that event 1 (T02) was part of a 5-second event during which response magnitude in ToM brain regions significantly correlated with GA; no other timepoints evoked differential responses by gestational age (Supplement S8).

In a post-hoc analysis with preterm-born children only (*n* = 30), the effect of GA on event 1 (T02) response magnitude was not statistically significant (ToM network: *p* = 0.585; RTPJ: *p* = 0.957).

**Fig. 4.**
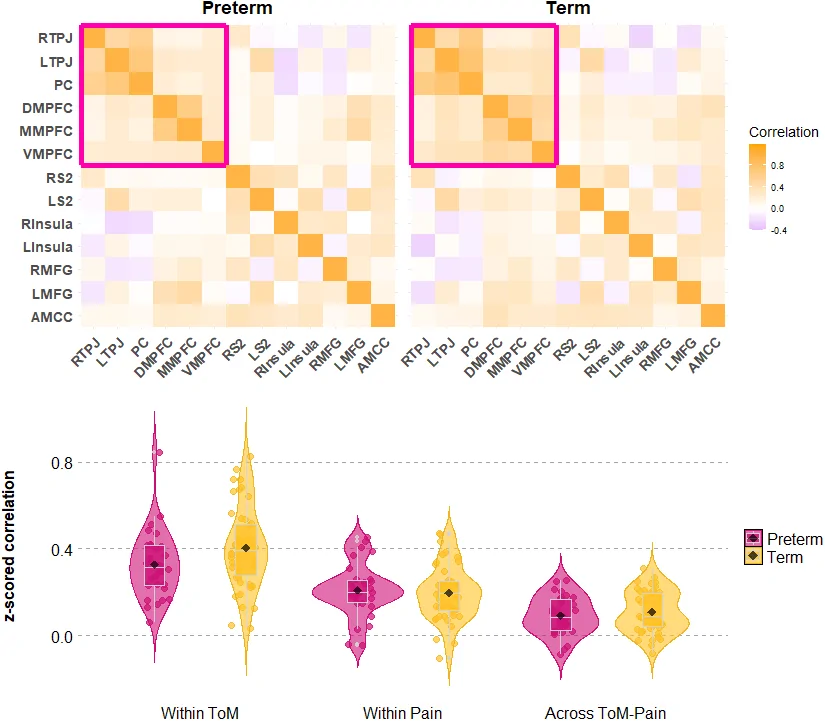
Inter–region correlations. Top row: average z-scored correlation matrices across all ToM and Pain Matrix ROIs (in the same order on x- and y-axes) for the preterm group (*n* = 30, left) and the term group (*n* = 46, right). The pink box highlights within ToM network correlations. Bottom row: box and violin plots show the within-ToM network (left), within-Pain network (middle), and across-ToM-Pain network z-scored correlation values (y-axes) per group (preterm in magenta, term in yellow). The horizontal line in the boxplot shows the median; the lower and upper hinges of the boxes correspond to the first and third quartiles, with the upper whisker extending to the largest value no further than 1.5 * inter-quartile range (IQR) from the hinge and the lower whisker extending from the lower hinge to the smallest value at most 1.5 * IQR of the hinge. Black diamonds indicate the group mean.

In preterm- and term-born children, the ToM network appeared to be functionally distinct from the Pain Matrix (a cortical network involved in processing bodily sensations, including pain): within-ToM network inter-region correlations were significantly higher than across-ToM-Pain network correlations in both groups (preterm: *t*(29) = 8.53, *p* = 1.059e-09; term: *t*(45) = 13.01, *p *< 2.2e-16; not pre-registered). Within-ToM correlations were significantly higher among children born at a later GA (Fig. 4; Table 2), but this effect did not survive correcting for multiple comparisons (α = 0.008, Bonferroni-adjusted threshold correcting for six neural measures) and was not statistically significant in the motion-matched subsample (preterm: *n *= 27, term *n *= 27; *p* = 0.157; Table 2), nor in separate analyses of preterm-born children only (to test for a dose-dependent effect; *n *= 30; *p* = 0.662).

**Fig. 5.**
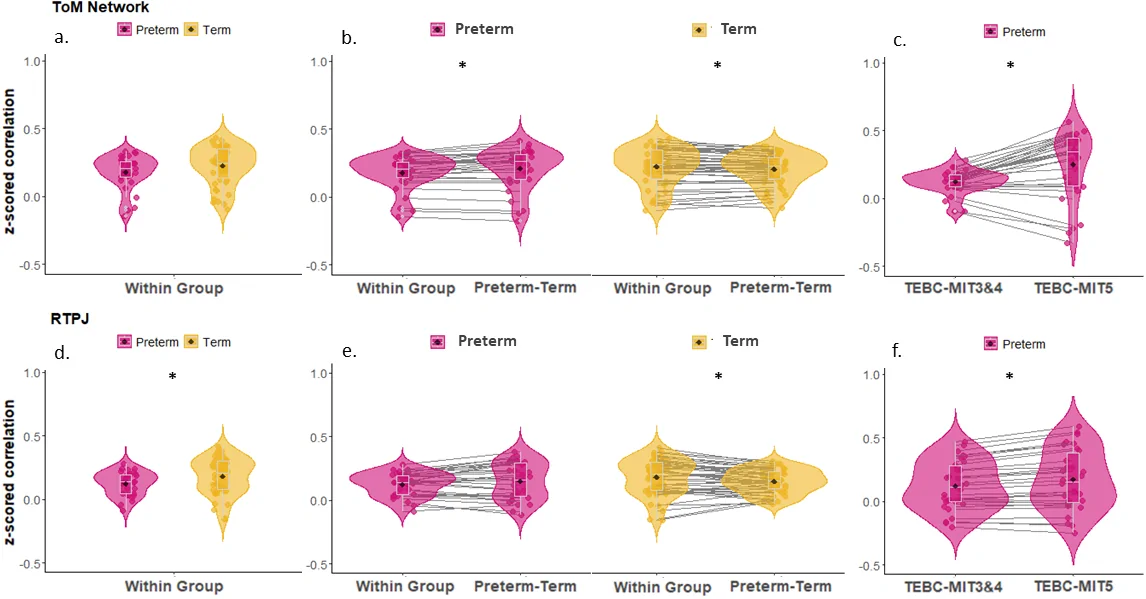
Intersubject correlations. Box and violin plots show the z-scored correlation values (y-axes) for each intersubject comparison (x-axes). The top row shows results for the ToM network, and the bottom row shows results for the RTPJ ROI separately. Panels a and d show the within-group intersubject correlations per group. Panels b and e show the comparison of the within-preterm or within-term group (“Within-Group”) intersubject correlations to the across group intersubject correlations (“Preterm-Term”). Panels c and f show the preterm to term-born 3- to 4-year-olds (*n* = 27, “MIT34”) or 5-year-olds (*n* = 30, “MIT5”) from the publicly available dataset. Preterm-born children are shown in magenta, and term-born children are shown in yellow. Asterisks indicate significant differences (*p *< 0.05). The horizontal line in the boxplot shows the median; the lower and upper hinges of the boxes correspond to the first and third quartiles, with the upper whisker extending to the largest value no further than 1.5 * inter-quartile range (IQR) from the hinge and the lower whisker extending from the lower hinge to the smallest value at most 1.5 * IQR of the hinge. Black diamonds indicate the group mean.

In intersubject correlation analyses on the full sample, ToM responses in both preterm- and term-born children were statistically most similar to those of (other) term-born children (Table 3; Fig. 5). Responses among preterm-born children were also statistically more similar to 5-year-old term-born children than to 3.5–4-year-old term-born children—providing evidence against delayed neural ToM development (Table 3; Fig. 5). In RTPJ (but not the full ToM network), preterm-born children showed more variable/dissimilar response timecourses than term-born children, in that the within-group intersubject correlation was statistically lower among preterm-born children (Table 3; Fig. 5). In the motion-matched sample, this group difference was statistically significant in RTPJ and the full ToM network (Table 3).

**Table 3.** Statistical results for intersubject correlation analyses.

Comparison	ROI(s)	Test	Full sample		Motion matched sample	
Q1: Are responses in children born preterm most like responses in other preterm-born children? Are responses in term-born children most like responses to other term-born children?						
Within PT - Across PT-T	ToM Network	1-tailed	*t(29) *= -3.84	*p* = 1.000	*t(26) =* -4.07	*p =* 1.000
	ToM Network	2-tailed (Not preregistered)	***t(29)*** ** = -3.84**	***p *** **= 0.001**	***t(26) = *** **-4.07**	***p < *** **0.001**
	RTPJ	1-tailed	*t(29) *= -1.63	*p *= 0.943	*t(26) = *-1.54	*p = *0.932
	RTPJ	2-tailed (Not preregistered)	*t(29) *= -1.63	*p *= 0.114	*t(26) = *-1.54	*p = *0.136
Within T - Across PT-T	ToM Network	1-tailed	***t(45) *** **= 3.04**	***p *** **= 0.002**	***t(26) = *** **4.29**	***p < *** **0.001**
	RTPJ	1-tailed	***t(45) *** **= 2.75**	***p *** **= 0.004**	***t(26) = *** **5.27**	***p < *** **0.001**
Q2: Do responses in children born preterm most resemble those of younger or age-matched term-born children?						
PT-3-4yo - PT-5yo	ToM Network	2-tailed	***t(29) *** **= -4.09**	***p < *** **0.001**	***t(26) = *** **-4.34**	***p < *** **0.001**
	RTPJ	2-tailed	***t(29) *** **= -5.24**	***p < *** **0.001**	***t(26) = *** **-5.08**	***p < *** **0.001**
Q3: Are responses in the preterm-born group more variable than in the term-born group?						
Within T - Within PT	ToM Network	1-tailed	*t(67.32) *= 1.48	*p *= 0.071	***t(51.99) = *** **2.22**	***p = *** **0.015**
	RTPJ	1-tailed	***t(73.98) *** **= 2.03**	***p *** **= 0.023**	***t(48.42) = *** **3.52**	***p < *** **0.001**

Statistical results for the intersubject correlation analyses for the full ToM network and RTPJ, separately, carried out in the full sample (*n* = 30 preterm, *n* = 46 term-born children) and the motion matched sub-sample (*n* = 27 children per group). “Comparison” indicates the groups for which means were compared (Within = within group, Across = across groups, T = term group, PT = preterm group, 3–4yo = three-to-four-year-olds from the MIT open dataset; 5yo = five-year-olds from the MIT open dataset), “ROI(s)” indicates which regions results are shown for and “Test” indicates whether a one- or two-tailed t-test was carried out. Statistics include t-values with degrees of freedom in brackets (*t(df)*), and p-values (*p*) for each predictor in the linear mixed model regression. Significant results (*p*-values <0.05) are shown in bold.

We conducted two additional non-preregistered analyses: (1) we tested the hypothesis that preterm-born children would show slower responses in ToM brain regions during movie-viewing. We did not find any evidence to support this hypothesis (Supplement S9). (2) We conducted two one-sided tests (Lakens et al., 2018) and region of practical equivalence (Kruschke, 2018) analyses for each neural metric. Unlike our pre-registered analyses, which were well-suited to test for significant effects of gestational age, these analyses can be used to statistically reject the presence of an effect of GA (or infer “statistical equivalence”). The results of these analyses were consistent with the pre-registered analyses (Supplement S10 and Supplementary Fig. S5). Briefly, these analyses provide statistical evidence for rejecting effects of gestational age larger than a standardised beta value of 0.1 for within-ToM IRC, across-ToM-Pain IRC, ToM network functional maturity, and effects of gestational age larger than 0.2 for RTPJ functional maturity and ToM and RTPJ response magnitude to events 2 (T01) and 3 (T04). Additionally, for all neural metrics except the within-ToM IRC measure, these results suggest that any effects of gestational age would be smaller than effects of test age (i.e., the effect of age among 5–5.86-year-old children)—and therefore, for example, smaller than individual differences observed among children of different ages within a shared classroom.

## 4 Discussion

This study used behavioural and movie-viewing fMRI measures of ToM with 5-year-old children to test whether ToM development is directly impacted by preterm birth. Preterm-born children performed less well than term-born comparators on a linguistic behavioural ToM measure but receptive language, rather than gestational age, was the strongest predictor of performance. Additionally, responses in ToM brain regions to a movie among preterm-born children were qualitatively similar to those observed in term-born comparators—and statistically more similar to same-aged term-born children than to other preterm-born children or younger term-born children. Together, this evidence suggests that being born early does not preclude broadly similar development of brain regions recruited for ToM at age 5 years and that differences in early ToM behaviour in preterm-born children may primarily reflect differences in other cognitive capacities, like language.

At age 5 years, preterm-born children (*n* = 52) scored lower than term-born children (*n* = 58) on the behavioural ToM measure and on a standardised receptive language measure. The effect of GA on overall ToM performance was significant even when statistically controlling for receptive language, which could suggest atypical/delayed ToM development among preterm-born children. However, these results are also consistent with linguistic task demands disproportionately affecting ToM performance among preterm-born children. The effect of GA on ToM performance was particularly strong for free response items, which evoked similar ToM concepts as 2AFC items but additionally required children to produce a linguistic response. Moreover, in a mixed-effects logistic regression, which provides a more sensitive test of effects of response format and item difficulty alongside effects of GA and language, there were main effects of language, response format, and item difficulty on performance, and a significant GA-by-response type interaction such that children born earliest provided fewer correct answers on free response questions; there was no main effect of GA on ToM performance. These results are consistent with prior behavioural research. For example, Witt et al. (2018), did not find evidence for differences in ToM reasoning between preterm- and term-born 5-year-old children who had similar verbal IQ (though note that the sample size was quite small, with 16 preterm-born children and 21 term-born children, meaning the study may have been underpowered to detect differences). Marleau et al. (2021) found lower language and ToM reasoning among preterm-born 5-year-old children. This pattern of results suggests that difficulty meeting the linguistic demands of behavioural ToM tasks may account for lower performance among 5-year-old children born preterm.

Our neural evidence is, on the whole, consistent with this interpretation. To test for a direct effect of GA on ToM development, a subset of children (*n *= 30 preterm, *n *= 46 term) contributed fMRI data acquired while viewing Disney Pixar’s “Partly Cloudy” (Reher & Sohn, 2009)—a movie that has been used in prior research to characterise development in ToM brain regions (Richardson et al., 2018). Children born preterm showed qualitatively similar functional responses in ToM brain regions while viewing “Partly Cloudy.” Whole-brain random effects analyses in preterm- and term-born children, separately, revealed the expected functional profile of activation with clusters in the bilateral temporoparietal junction, precuneus, and medial prefrontal cortex in both groups of children. Further, across the full sample, no significant clusters showed greater activity to the ToM (Mind>Body) contrast as a function of GA. This same pattern of results was present in samples tightly matched on data quality and sample size (*n *= 27 per group) and is consistent with prior research with older children (Mossad et al., 2021). However, whole-brain analyses rely on commonalities in the spatial layout of functional responses across individuals (Saxe, Brett, et al., 2006) and require stringent corrections for multiple comparisons (Eklund et al., 2016), which can make them less sensitive than ROI analyses. ROI analyses are particularly useful when there is strong prior evidence to motivate testing hypotheses in a small set of regions. Given prior evidence for the reliable recruitment of the ToM network during ToM reasoning (relative to control tasks) in adults and children (Carrington & Bailey, 2009; Gweon et al., 2012; Richardson et al., 2018; Saxe & Kanwisher, 2003) and prior paediatric fMRI research characterising development in ToM brain region responses during the same movie-viewing experiment used here (Richardson et al., 2018), we used ROI analyses to test for effects of GA specifically in ToM brain regions.

Our ROI analyses did not detect a significant effect of gestational age for several neural ToM measures: there was not a statistically significant effect of GA on how “adult-like” response timecourses in the ToM network were; nor on the ToM network response magnitude to almost the entire movie. In inter-region correlation analyses, there was not a statistically significant effect of GA on how functionally distinct ToM brain regions were from a second cortical network (the “Pain Matrix”, recruited to reason about others’ bodies; Bruneau et al., 2012). These analyses provided some evidence for weaker functional correlations within the ToM network, but this result did not survive conservative correction for multiple comparisons across our neural ToM measures and was not significant in the motion-matched subsample (but this null result could be a consequence of matched motion or the smaller sample size). Post-hoc equivalence tests suggested that any effects of gestational age on these metrics would be small, and, for most measures, smaller than the effect of age among 5-year-old children.

In line with this evidence, intersubject correlation analyses showed that ToM brain region responses of preterm-born children were most similar to those of 5-year-old children born at term, rather than to other preterm-born children or to 3-to-4-year-old children born at term. However, preterm-born children also had more variable (i.e., less stimulus driven) ToM responses, relative to term-born children, even in the motion-matched subsample. Increased variability in responses among preterm-born children could reflect differences in brain structure underlying ToM regions, like white matter tract integrity (Deferm et al., 2024; Dimitrova et al., 2021; Nosarti et al., 2014)—which could lead to idiosyncrasies in the timing of the ToM neural response to the movie. This variability is also consistent with the substantial heterogeneity in development across individuals born early, across domains (Johnson & Marlow, 2017). This result provides some evidence for different neural ToM development among 5-year-old preterm-born children; however, despite the increased variability in responses among preterm-born children, their individual neural ToM responses were still most similar to those of their term-born peers.

One additional neural measure suggested neural ToM differences in preterm-born children: the response magnitude in ToM brain regions to one movie scene (event 1 (T02)) was higher in children born later. However, visual inspection of the response timecourses showed that the average response among preterm-born children and adults appeared to peak slightly earlier than that of term-born children (Fig. 3). It is difficult to know if preterm-born children’s responses to this scene are more adult-like than those of term-born children—which would argue against the interpretation that the different response reflects developmental delay among children born preterm—or if responses among preterm-born children are less sustained than those of their term-born peers. The 5 consecutive seconds that showed differences by GA (Supplement S8), and which include event 1 (T02), correspond to a part of the movie that shows consecutive close-ups of multiple characters with dynamic emotional expressions (i.e., Gus (the cloud) expresses sadness while looking from his animal bundle over to Peck (the stork), who expresses adoration at the cute babies made by another cloud; Peck realises Gus is watching him, at which point he appears embarrassed. Gus then offers his animal bundle to Peck, hopefully). Speculatively, the opportunity for quick, successive mental state inferences for multiple characters could make this scene particularly sensitive to differences between children born preterm and at term—but additional research is necessary to replicate and understand this result.

The broad similarities in ToM brain region function between 5-year-old preterm- and term-born children may be surprising given observed differences in real-world social behaviours in children and adults born preterm (Mendonça et al., 2019; Ritchie et al., 2018). One potential explanation is that these differences primarily reflect differences in other cognitive capacities that affect performance on ToM behavioural tasks and real-world social behaviours, like language (Dean et al., 2021; Ritchie et al., 2018; van Noort-van der Spek et al., 2012). While we do not find evidence for effects of preterm-birth on attention or executive functions in our sample, the evidence presented here for reduced receptive language among children born preterm is consistent with prior behavioural research (van Noort-van der Spek et al., 2012). A growing body of neuroimaging research finds associations between atypical brain structure and language delays in preterm-born children and adolescents (for review see Kvanta et al., 2024; Stipdonk et al., 2018; Valavani et al., 2022); there is also evidence for decreased functional connectivity among language regions that correlates with language outcomes in young children born preterm (Choi et al., 2018; though see also Barnes‐Davis et al., 2018). Movie-viewing fMRI experiments may be particularly useful for characterising differences in language development in young children born preterm, in future research (Olson et al., 2026). Additionally, research investigating the broader impacts of language interventions may be particularly important for supporting children born preterm. Language facilitates engagement in rich social interactions (de Rosnay & Hughes, 2006) and directly drives ToM development (Richardson, Koster-Hale, et al., 2020; Ruffman et al., 2002), which continues beyond age 5 years (Peterson & Wellman, 2019). Even if early ToM development is preserved among 5-year-old children born preterm, as our evidence suggests, prolonged language differences could have cascading effects on subsequent ToM development and social development more broadly.

A second, related (and non-mutually exclusive) explanation is that many real-world social behaviours require more complex ToM reasoning than that evoked by “Partly Cloudy”—which does not have overt linguistic processing demands and was designed to be understood by young children. Consistent with this explanation, preterm-born children performed similarly to term-born children on the behavioural task assessing movie-specific ToM reasoning. The neural and behavioural ToM measures used here also measure differences in third-person ToM inferences rather than first-person social interactions. It may be that children born preterm struggle to coordinate the multiple cognitive processes engaged when participating in first-person social interactions—perhaps due to distributed differences in white matter microstructure early in life (Deferm et al., 2024; Kelly et al., 2016). Additional research using experiments that evoke more sophisticated ToM inferences and measures of first-person social interactions (Misaki et al., 2021; Redcay & Schilbach, 2019) is needed.

Our approach used fMRI during movie-viewing, with whole-brain random effects analysis and ROI-based analyses in an a priori defined ToM network. While our analysis approach was hypothesis driven and builds directly on prior research of neural ToM development in early childhood (Richardson, 2019, Richardson et al., 2018), it is just one approach for studying neural ToM development in children born preterm. Future research could aim to acquire and analyse more fMRI data per participant to support data-driven definition of functional networks—to more sensitively test whether functional network organisation differs as a function of GA (e.g., Carrasco-Solís et al., 2026). Additionally, acquiring multimodal data, including MEG, may provide a fuller picture of early neural ToM development. Prior research with older children (7+ years of age) provides some evidence for neural differences during ToM reasoning as a function of GA, including reduced functional connectivity across several regions in very preterm-born children (Mossad et al., 2017, 2021). If real-time coordination of multiple cognitive processes is a core part of social differences among children born preterm, then measures with high temporal resolution, like MEG, may be particularly useful in future research. This evidence for differences in neural ToM responses among older children is also consistent with the idea that broadly similar ToM development among young children born preterm does not preclude larger differences in ToM later in development, perhaps as a consequence of early language differences.

## 5 Conclusion

To our knowledge, this study provides the earliest characterisation of neural ToM responses among children born preterm, with a focus on 5-year-old children. Overall, the evidence presented here suggests that differences in preterm-born infants’ earliest social experiences and brain structure (Eyre et al., 2021; Gozdas et al., 2018; Thompson et al., 2020) do not preclude broadly similar functional development in ToM brain regions at age 5 years. Functional plasticity early in life may enable many preterm-born children to “catch up” on early ToM reasoning. Interventions to support preterm-born children are therefore likely to require attention to other cognitive capacities that facilitate social behaviour, including language.

## Supplementary Material

Supplementary Material

## Data Availability

Requests for anonymised data for reproducing the statistical analyses of this study and raw, de-identified (f)MRI data can be made by completing a Data Access Request form at https://reproductive-health.ed.ac.uk/theirworld-edinburgh-birth-cohort-tebc/for-researchers/data-access-and-collaboration. The fMRI analysis pipeline (https://github.com/hrichardsonlab/fmri-analysis) and statistical analysis code (https://osf.io/xtyu8/overview) are publicly available.

## References

[IMAG.a.1347-b1] Barnes‐Davis, M. E., Merhar, S. L., Holland, S. K., & Kadis, D. S. (2018). Extremely preterm children exhibit increased interhemispheric connectivity for language: Findings from fMRI ‐constrained MEG analysis. Developmental Science, 21(6), e12669. 10.1111/desc.12669 29659125 PMC6193851

[IMAG.a.1347-b2] Behzadi, Y., Restom, K., Liau, J., & Liu, T. T. (2007). A component based noise correction method (CompCor) for BOLD and perfusion based fMRI. NeuroImage, 37(1), 90–101. 10.1016/j.neuroimage.2007.04.042 17560126 PMC2214855

[IMAG.a.1347-b3] Blank, I., Kanwisher, N., & Fedorenko, E. (2014). A functional dissociation between language and multiple-demand systems revealed in patterns of BOLD signal fluctuations. Journal of Neurophysiology, 112(5), 1105–1118. 10.1152/jn.00884.2013 24872535 PMC4122731

[IMAG.a.1347-b4] Boardman, J. P., Andrew, R., Bastin, M. E., Battersby, C., Batty, G. D., Cábez, M. B., Cox, S. R., Hall, J., Ingledow, L., Marioni, R. E., Modi, N., Murphy, L., Quigley, A. J., Reynolds, R. M., Richardson, H., Stock, S. J., Thrippleton, M. J., Tsanas, A., & Whalley, H. C. (2024). Preterm birth as a determinant of neurodevelopment and cognition in children (PRENCOG): Protocol for an exposure-based cohort study in the UK. BMJ Open, 14(9), e085365. 10.1136/bmjopen-2024-085365 PMC11409314 39284691

[IMAG.a.1347-b5] Boardman, J. P., Hall, J., Thrippleton, M. J., Reynolds, R. M., Bogaert, D., Davidson, D. J., Schwarze, J., Drake, A. J., Chandran, S., Bastin, M. E., & Fletcher-Watson, S. (2020). Impact of preterm birth on brain development and long-term outcome: Protocol for a cohort study in Scotland. BMJ Open, 10(3), e035854. 10.1136/bmjopen-2019-035854 PMC7059503 32139495

[IMAG.a.1347-b6] Brogan, E., Cragg, L., Gilmore, C., Marlow, N., Simms, V., & Johnson, S. (2014). Inattention in very preterm children: Implications for screening and detection. Archives of Disease in Childhood, 99(9), 834–839. 10.1136/archdischild-2013-305532 24842798

[IMAG.a.1347-b7] Bruneau, E. G., Jacoby, N., & Saxe, R. (2015). Empathic control through coordinated interaction of amygdala, theory of mind and extended pain matrix brain regions. NeuroImage, 114, 105–119. 10.1016/j.neuroimage.2015.04.034 25913703

[IMAG.a.1347-b8] Bruneau, E. G., Pluta, A., & Saxe, R. (2012). Distinct roles of the ‘Shared Pain’ and ‘Theory of Mind’ networks in processing others’ emotional suffering. Neuropsychologia, 50(2), 219–231. 10.1016/j.neuropsychologia.2011.11.008 22154962

[IMAG.a.1347-b9] Cantlon, J. F., & Li, R. (2013). Neural activity during natural viewing of sesame street statistically predicts test scores in early childhood. PLoS Biology, 11(1), e1001462. 10.1371/journal.pbio.1001462 23300385 PMC3536813

[IMAG.a.1347-b10] Carp, J. (2013). Optimizing the order of operations for movement scrubbing: Comment on Power et al. NeuroImage, 76, 436–438. 10.1016/j.neuroimage.2011.12.061 22227884

[IMAG.a.1347-b11] Carrasco-Solís, M. E., Amaoui, S., Laynez-Rubio, C., Nieto-Ruiz, A., Ruiz-López, A., Fernández-Marín, E., Campos-Martínez, A., Molina-Oya, M., & Uberos-Fernández, J. (2026). Resting-state networks in school-aged very preterm children: Links with cognition and theory of mind. Social Cognitive and Affective Neuroscience, nsag010. 10.1093/scan/nsag010 41702370

[IMAG.a.1347-b12] Carrington, S. J., & Bailey, A. J. (2009). Are there theory of mind regions in the brain? A review of the neuroimaging literature. Human Brain Mapping, 30(8), 2313–2335. 10.1002/hbm.20671 19034900 PMC6871093

[IMAG.a.1347-b201] Choi, E. J., Vandewouw, M. M., Young, J. M., & Taylor, M. J. (2018). Language network function in young children born very preterm. Frontiers in Human Neuroscience, 12, 512. 10.3389/fnhum.2018.00512 30618688 PMC6306484

[IMAG.a.1347-b13] de Rosnay, M., & Hughes, C. (2006). Conversation and theory of mind: Do children talk their way to socio-cognitive understanding? British Journal of Developmental Psychology, 24(1), 7–37. 10.1348/026151005X82901

[IMAG.a.1347-b14] Dean, B., Ginnell, L., Boardman, J. P., & Fletcher-Watson, S. (2021). Social cognition following preterm birth: A systematic review. Neuroscience & Biobehavioral Reviews, 124, 151–167. 10.1016/j.neubiorev.2021.01.006 33524414

[IMAG.a.1347-b15] Deferm, W., Tang, T., Moerkerke, M., Daniels, N., Steyaert, J., Alaerts, K., Ortibus, E., Naulaers, G., & Boets, B. (2024). Subtle microstructural alterations in white matter tracts involved in socio-emotional processing after very preterm birth. NeuroImage: Clinical, 41, 103580. 10.1016/j.nicl.2024.103580 38401459 PMC10944182

[IMAG.a.1347-b202] Dimitrova, R., Pietsch, M., Ciarrusta, J., Fitzgibbon, S. P., Williams, L. Z. J., Christiaens, D., Cordero-Grande, L., Batalle, D., Makropoulos, A., Schuh, A., Price, A. N., Hutter, J., Teixeira, R. P., Hughes, E., Chew, A., Falconer, S., Carney, O., Egloff, A., Tournier, J.-D., … O’Muircheartaigh,J. (2021). Preterm birth alters the development of cortical microstructure and morphology at term-equivalent age. NeuroImage, 243, 118488. 10.1016/j.neuroimage.2021.118488 34419595 PMC8526870

[IMAG.a.1347-b16] Eklund, A., Nichols, T. E., & Knutsson, H. (2016). Cluster failure: Why fMRI inferences for spatial extent have inflated false-positive rates. Proceedings of the National Academy of Sciences of the United States of America, 113(28), 7900–7905. 10.1073/pnas.1602413113 27357684 PMC4948312

[IMAG.a.1347-b17] Ereky-Stevens, K. (2008). Associations between mothers’ sensitivity to their infants’ internal states and children’s later understanding of mind and emotion. Infant and Child Development, 17(5), 527–543. 10.1002/icd.572

[IMAG.a.1347-b18] Esteban, O., Ciric, R., Finc, K., Blair, R. W., Markiewicz, C. J., Moodie, C. A., Kent, J. D., Goncalves, M., DuPre, E., Gomez, D. E. P., Ye, Z., Salo, T., Valabregue, R., Amlien, I. K., Liem, F., Jacoby, N., Stojić, H., Cieslak, M., Urchs, S., … Gorgolewski, K. J. (2020). Analysis of task-based functional MRI data preprocessed with fMRIPrep. Nature Protocols, 15(7), 2186–2202. 10.1038/s41596-020-0327-3 32514178 PMC7404612

[IMAG.a.1347-b19] Esteban, O., Markiewicz, C., Blair, R. W., Moodie, C., Isik, A. I., Erramuzpe Aliaga, A., Kent, J., Goncalves, M., DuPre, E., Snyder, M., Oya, H., Ghosh, S., Wright, J., Durnez, J., Poldrack, R., & Gorgolewski, K. J. (2019). fMRIPrep: A robust preprocessing pipeline for functional MRI. Nature Methods, 16, 111–116. 10.1038/s41592-018-0235-4 30532080 PMC6319393

[IMAG.a.1347-b20] Eyre, M., Fitzgibbon, S. P., Ciarrusta, J., Cordero-Grande, L., Price, A. N., Poppe, T., Schuh, A., Hughes, E., O’Keeffe, C., Brandon, J., Cromb, D., Vecchiato, K., Andersson, J., Duff, E. P., Counsell, S. J., Smith, S. M., Rueckert, D., Hajnal, J. V., Arichi, T., … Edwards, A. D. (2021). The Developing Human Connectome Project: Typical and disrupted perinatal functional connectivity. Brain, 144(7), 2199–2213. 10.1093/brain/awab118 33734321 PMC8370420

[IMAG.a.1347-b21] Fedorenko, E., Behr, M. K., & Kanwisher, N. (2011). Functional specificity for high-level linguistic processing in the human brain. Proceedings of the National Academy of Sciences of the United States of America, 108(39), 16428–16433. 10.1073/pnas.1112937108 21885736 PMC3182706

[IMAG.a.1347-b22] Fenoglio, A., Georgieff, M. K., & Elison, J. T. (2017). Social brain circuitry and social cognition in infants born preterm. Journal of Neurodevelopmental Disorders, 9(1), 27. 10.1186/s11689-017-9206-9 28728548 PMC5516343

[IMAG.a.1347-b23] Firestein, M. R., Myers, M. M., Feder, K. J., Ludwig, R. J., & Welch, M. G. (2022). Effects of family nurture intervention in the NICU on theory of mind abilities in children born very preterm: A randomized controlled trial. Children, 9(2), Article 2. 10.3390/children9020284 PMC8870221 35205004

[IMAG.a.1347-b24] Gorgolewski, K. J., Alfaro-Almagro, F., Auer, T., Bellec, P., Capotă, M., Chakravarty, M. M., Churchill, N. W., Cohen, A. L., Craddock, R. C., Devenyi, G. A., Eklund, A., Esteban, O., Flandin, G., Ghosh, S. S., Guntupalli, J. S., Jenkinson, M., Keshavan, A., Kiar, G., Liem, F., … Poldrack, R. A. (2017). BIDS apps: Improving ease of use, accessibility, and reproducibility of neuroimaging data analysis methods. PLoS Computational Biology, 13(3), e1005209. 10.1371/journal.pcbi.1005209 28278228 PMC5363996

[IMAG.a.1347-b25] Gorgolewski, K. J., Esteban, O., Markiewicz, C. J., Ziegler, E., Ellis, D. G., Notter, M. P., Jarecka, D., Johnson, H., Burns, C., Manhães-Savio, A., Hamalainen, C., Yvernault, B., Salo, T., Jordan, K., Goncalves, M., Waskom, M., Clark, D., Wong, J., Loney, F., … Ghosh, S. (2018). Nipype. Software. 10.5281/zenodo.596855

[IMAG.a.1347-b26] Gozdas, E., Parikh, N. A., Merhar, S. L., Tkach, J. A., He, L., & Holland, S. K. (2018). Altered functional network connectivity in preterm infants: Antecedents of cognitive and motor impairments? Brain Structure and Function, 223(8), 3665–3680. 10.1007/s00429-018-1707-0 29992470 PMC6892636

[IMAG.a.1347-b27] Guarini, A., Marini, A., Savini, S., Alessandroni, R., Faldella, G., & Sansavini, A. (2016). Linguistic features in children born very preterm at preschool age. Developmental Medicine & Child Neurology, 58(9), 949–956. 10.1111/dmcn.13118 27061384

[IMAG.a.1347-b28] Gweon, H., Dodell-Feder, D., Bedny, M., & Saxe, R. (2012). Theory of mind performance in children correlates with functional specialization of a brain region for thinking about thoughts: Behavioral and neural development in theory of mind. Child Development, 83(6), 1853–1868. 10.1111/j.1467-8624.2012.01829.x 22849953

[IMAG.a.1347-b29] Hallquist, M. N., Hwang, K., & Luna, B. (2013). The nuisance of nuisance regression: Spectral misspecification in a common approach to resting-state fMRI preprocessing reintroduces noise and obscures functional connectivity. NeuroImage, 82, 208–225. 10.1016/j.neuroimage.2013.05.116 23747457 PMC3759585

[IMAG.a.1347-b30] Hasson, U., Nir, Y., Levy, I., Fuhrmann, G., & Malach, R. (2004). Intersubject synchronization of cortical activity during natural vision. Science, 303(5664), 1634–1640. 10.1126/science.1089506 15016991

[IMAG.a.1347-b31] Hughes, C., & Ensor, R. (2007). Executive function and theory of mind: Predictive relations from ages 2 to 4. Developmental Psychology, 43(6), 1447–1459. 10.1037/0012-1649.43.6.1447 18020823

[IMAG.a.1347-b32] Inder, T. E., Volpe, J. J., & Anderson, P. J. (2023). Defining the neurologic consequences of preterm birth. New England Journal of Medicine, 389(5), 441–453. 10.1056/NEJMra2303347 37530825

[IMAG.a.1347-b203] Jacoby, N., Bruneau, E., Koster-Hale, J., & Saxe, R. (2016). Localizing Pain Matrix and Theory of Mind networks with both verbal and non-verbal stimuli. NeuroImage, 126, 39–48. 10.1016/j.neuroimage.2015.11.025 26589334 PMC4733571

[IMAG.a.1347-b33] Johnson, S., & Marlow, N. (2017). Early and long-term outcome of infants born extremely preterm. Archives of Disease in Childhood, 102(1), 97–102. 10.1136/archdischild-2015-309581 27512082

[IMAG.a.1347-b34] Jones, K. M., Champion, P. R., & Woodward, L. J. (2013). Social competence of preschool children born very preterm. Early Human Development, 89(10), 795–802. 10.1016/j.earlhumdev.2013.06.008 23870752 PMC4271316

[IMAG.a.1347-b35] Kelly, C. E., Cheong, J. L. Y., Gabra Fam, L., Leemans, A., Seal, M. L., Doyle, L. W., Anderson, P. J., Spittle, A. J., & Thompson, D. K. (2016). Moderate and late preterm infants exhibit widespread brain white matter microstructure alterations at term-equivalent age relative to term-born controls. Brain Imaging and Behavior, 10(1), 41–49. 10.1007/s11682-015-9361-0 25739350

[IMAG.a.1347-b36] Knudsen, B., & Liszkowski, U. (2012). 18-Month-olds predict specific action mistakes through attribution of false belief, not ignorance, and intervene accordingly. Infancy, 17(6), 672–691. 10.1111/j.1532-7078.2011.00105.x 32693489

[IMAG.a.1347-b37] Kruschke, J. K. (2018). Rejecting or accepting parameter values in Bayesian estimation. Advances in Methods and Practices in Psychological Science, 1(2), 270–280. 10.1177/2515245918771304

[IMAG.a.1347-b38] Kvanta, H., Bolk, J., Broström, L., Nosko, D., Fernández De Gamarra-Oca, L., Padilla, N., & Ådén, U. (2024). Language performance and brain volumes, asymmetry, and cortical thickness in children born extremely preterm. Pediatric Research, 95(4), 1070–1079. 10.1038/s41390-023-02871-0 37923870 PMC10920199

[IMAG.a.1347-b39] Lakens, D., Scheel, A. M., & Isager, P. M. (2018). Equivalence testing for psychological research: A tutorial. Advances in Methods and Practices in Psychological Science, 1(2), 259–269. 10.1177/2515245918770963

[IMAG.a.1347-b40] Marleau, I., Vona, M., Gagner, C., Luu, T. M., & Beauchamp, M. H. (2021). Social cognition, adaptive functioning, and behavior problems in preschoolers born extremely preterm. Child Neuropsychology, 27(1), 96–108. 10.1080/09297049.2020.1797656 32716689

[IMAG.a.1347-b41] Mars, R. B., Sallet, J., Schuffelgen, U., Jbabdi, S., Toni, I., & Rushworth, M. F. S. (2012). Connectivity-based subdivisions of the human right ‘temporoparietal junction area’: Evidence for different areas participating in different cortical networks. Cerebral Cortex, 22(8), 1894–1903. 10.1093/cercor/bhr268 21955921

[IMAG.a.1347-b42] Mendonça, M., Bilgin, A., & Wolke, D. (2019). Association of preterm birth and low birth weight with romantic partnership, sexual intercourse, and parenthood in adulthood: A systematic review and meta-analysis. JAMA Network Open, 2(7), e196961. 10.1001/jamanetworkopen.2019.6961 31298716 PMC6628597

[IMAG.a.1347-b43] Milligan, K., Astington, J. W., & Dack, L. A. (2007). Language and theory of mind: Meta-analysis of the relation between language ability and false-belief understanding. Child Development, 78(2), 622–646. 10.1111/j.1467-8624.2007.01018.x 17381794

[IMAG.a.1347-b44] Misaki, M., Kerr, K. L., Ratliff, E. L., Cosgrove, K. T., Simmons, W. K., Morris, A. S., & Bodurka, J. (2021). Beyond synchrony: The capacity of fMRI hyperscanning for the study of human social interaction. Social Cognitive and Affective Neuroscience, 16(1–2), 84–92. 10.1093/scan/nsaa143 33104783 PMC7812622

[IMAG.a.1347-b45] Mossad, S. I., Smith, M. L., Pang, E. W., & Taylor, M. J. (2017). Neural correlates of “Theory of Mind” in very preterm born children: Theory of mind in very preterm born children. Human Brain Mapping, 38(11), 5577–5589. 10.1002/hbm.23750 28766907 PMC6866839

[IMAG.a.1347-b46] Mossad, S. I., Vandewouw, M. M., Smith, M. L., & Taylor, M. J. (2021). The preterm social brain: Altered functional networks for Theory of Mind in very preterm children. Brain Communications, 3(1), fcaa237. 10.1093/braincomms/fcaa237 33615217 PMC7882208

[IMAG.a.1347-b47] Mullen, E. M. (1995). Mullen scales of early learning (pp. 58–64). MN: AGS. https://www.pearsonclinical.co.uk/en-gb/Store/Professional-Assessments/Developmental-Early-Childhood/Mullen-Scales-of-Early-Learning/p/P100009037?srsltid=AfmBOooOoHF8rgAAvrw0JLsEbVZGPG240v5yI4LySccF6r0JJR7yoZa6

[IMAG.a.1347-b48] Nastase, S. A., Gazzola, V., Hasson, U., & Keysers, C. (2019). Measuring shared responses across subjects using intersubject correlation. Social Cognitive and Affective Neuroscience, 14(6), 667–685. 10.1093/scan/nsz037 31099394 PMC6688448

[IMAG.a.1347-b49] Nosarti, C., Nam, K. W., Walshe, M., Murray, R. M., Cuddy, M., Rifkin, L., & Allin, M. P. G. (2014). Preterm birth and structural brain alterations in early adulthood. NeuroImage: Clinical, 6, 180–191. 10.1016/j.nicl.2014.08.005 25379430 PMC4215396

[IMAG.a.1347-b50] Ohuma, E. O., Moller, A.-B., Bradley, E., Chakwera, S., Hussain-Alkhateeb, L., Lewin, A., Okwaraji, Y. B., Mahanani, W. R., Johansson, E. W., Lavin, T., Fernandez, D. E., Domínguez, G. G., Costa, A. de, Cresswell, J. A., Krasevec, J., Lawn, J. E., Blencowe, H., Requejo, J., & Moran, A. C. (2023). National, regional, and global estimates of preterm birth in 2020, with trends from 2010: A systematic analysis. The Lancet, 402(10409), 1261–1271. 10.1016/S0140-6736(23)00878-4 37805217

[IMAG.a.1347-b51] Olson, H. A., Chen, E. M., Osumah, C. J., Du, H., Fedorenko, E., & Saxe, R. (2026). The emergence of the language system in the toddler brain. Neuroscience. 10.64898/2026.02.26.707550

[IMAG.a.1347-b52] O’Meagher, S., Kemp, N., Norris, K., Anderson, P., & Skilbeck, C. (2017). Risk factors for executive function difficulties in preschool and early school-age preterm children. Acta Paediatrica, 106(9), 1468–1473. 10.1111/apa.13915 28502114

[IMAG.a.1347-b53] Pace, C. C., Spittle, A. J., Molesworth, C. M.-L., Lee, K. J., Northam, E. A., Cheong, J. L. Y., Davis, P. G., Doyle, L. W., Treyvaud, K., & Anderson, P. J. (2016). Evolution of depression and anxiety symptoms in parents of very preterm infants during the newborn period. JAMA Pediatrics, 170(9), 863–870. 10.1001/jamapediatrics.2016.0810 27428766

[IMAG.a.1347-b54] Patel, R. M., Rysavy, M. A., Bell, E. F., & Tyson, J. E. (2017). Survival of infants born at periviable gestational ages. Clinics in Perinatology, 44(2), 287–303. 10.1016/j.clp.2017.01.009 28477661 PMC5424630

[IMAG.a.1347-b55] Paunov, A. M., Blank, I. A., Jouravlev, O., Mineroff, Z., Gallée, J., & Fedorenko, E. (2022). Differential tracking of linguistic vs. mental state content in naturalistic stimuli by language and theory of mind (ToM) brain networks. Neurobiology of Language, 3(3), 413–440. 10.1162/nol_a_00071 37216061 PMC10158571

[IMAG.a.1347-b56] Pavarini, G., de Hollanda Souza, D., & Hawk, C. K. (2013). Parental practices and theory of mind development. Journal of Child and Family Studies, 22(6), 844–853. 10.1007/s10826-012-9643-8

[IMAG.a.1347-b57] Peterson, C. C., & Wellman, H. M. (2019). Longitudinal theory of mind (ToM) development from preschool to adolescence with and without ToM delay. Child Development, 90(6), 1917–1934. 10.1111/cdev.13064 29660808

[IMAG.a.1347-b58] Power, J. D., Barnes, K. A., Snyder, A. Z., Schlaggar, B. L., & Petersen, S. E. (2012). Spurious but systematic correlations in functional connectivity MRI networks arise from subject motion. NeuroImage, 59(3), 2142–2154. 10.1016/j.neuroimage.2011.10.018 22019881 PMC3254728

[IMAG.a.1347-b59] Rakoczy, H. (2022). Foundations of theory of mind and its development in early childhood. Nature Reviews Psychology, 1(4), 223–235. 10.1038/s44159-022-00037-z

[IMAG.a.1347-b60] Redcay, E., & Schilbach, L. (2019). Using second-person neuroscience to elucidate the mechanisms of social interaction. Nature Reviews. Neuroscience, 20(8), 495–505. 10.1038/s41583-019-0179-4 31138910 PMC6997943

[IMAG.a.1347-b61] Reher, K., & Sohn, P. (Directors). (2009). Partly Cloudy [Film]. Pixar Animation Studios and Walt Disney Pictures. https://www.pixar.com/partly-cloudy

[IMAG.a.1347-b62] Richardson, H. (2019). Development of brain networks for social functions: Confirmatory analyses in a large open source dataset. Developmental Cognitive Neuroscience, 37, 100598. 10.1016/j.dcn.2018.11.002 30522854 PMC6969289

[IMAG.a.1347-b63] Richardson, H., Gweon, H., Dodell-Feder, D., Malloy, C., Pelton, H., Keil, B., Kanwisher, N., & Saxe, R. (2020). Response patterns in the developing social brain are organized by social and emotion features and disrupted in children diagnosed with autism spectrum disorder. Cortex, 125, 12–29. 10.1016/j.cortex.2019.11.021 31958654

[IMAG.a.1347-b64] Richardson, H., Koster-Hale, J., Caselli, N., Magid, R., Benedict, R., Olson, H., Pyers, J., & Saxe, R. (2020). Reduced neural selectivity for mental states in deaf children with delayed exposure to sign language. Nature Communications, 11(1), 3246. 10.1038/s41467-020-17004-y PMC7319957 32591503

[IMAG.a.1347-b65] Richardson, H., Lisandrelli, G., Riobueno-Naylor, A., & Saxe, R. (2018). Development of the social brain from age three to twelve years. Nature Communications, 9(1), 1027. 10.1038/s41467-018-03399-2 PMC5847587 29531321

[IMAG.a.1347-b66] Richardson, H., & Saxe, R. (2020). Development of predictive responses in theory of mind brain regions. Developmental Science, 23(1), e12863. 10.1111/desc.12863 31125472

[IMAG.a.1347-b67] Ritchie, K., Bora, S., & Woodward, L. J. (2018). Peer relationship outcomes of school-age children born very preterm. The Journal of Pediatrics, 201, 238–244. 10.1016/j.jpeds.2018.05.034 29958672

[IMAG.a.1347-b68] Roldán-Tapia, M. D., Moreno-Ríos, S., & Cánovas-López, R. (2017). Thinking about social and nonsocial alternative possibilities in premature preschoolers. Journal of Clinical and Experimental Neuropsychology, 39(8), 725–737. 10.1080/13803395.2016.1257703 27892813

[IMAG.a.1347-b69] Ruffman, T., Slade, L., & Crowe, E. (2002). The relation between children’s and mothers? Mental state language and theory-of-mind understanding. Child Development, 73(3), 734–751. 10.1111/1467-8624.00435 12038548

[IMAG.a.1347-b70] Sabbagh, M. A., Bowman, L. C., Evraire, L. E., & Ito, J. M. B. (2009). Neurodevelopmental correlates of theory of mind in preschool children. Child Development, 80(4), 1147–1162. 10.1111/j.1467-8624.2009.01322.x 19630899

[IMAG.a.1347-b71] Sandoval, C. C., Gaspardo, C. M., & Linhares, M. B. M. (2022). The impact of preterm birth on the executive functioning of preschool children: A systematic review. Applied Neuropsychology: Child, 11(4), 873–890. 10.1080/21622965.2021.1915145 33984255

[IMAG.a.1347-b72] Saxe, R., Brett, M., & Kanwisher, N. (2006). Divide and conquer: A defense of functional localizers. NeuroImage, 30(4), 1088–1096. 10.1016/j.neuroimage.2005.12.062 16635578

[IMAG.a.1347-b73] Saxe, R., & Kanwisher, N. (2003). People thinking about thinking people. The role of the temporo-parietal junction in “theory of mind”. NeuroImage, 19(4), 1835–1842. 10.1016/S1053-8119(03)00230-1 12948738

[IMAG.a.1347-b74] Saxe, R., Schulz, L. E., & Jiang, Y. V. (2006). Reading minds versus following rules: Dissociating theory of mind and executive control in the brain. Social Neuroscience, 1(3–4), 284–298. 10.1080/17470910601000446 18633794

[IMAG.a.1347-b75] Scholz, J., Triantafyllou, C., Whitfield-Gabrieli, S., Brown, E. N., & Saxe, R. (2009). Distinct regions of right temporo-parietal junction are selective for theory of mind and exogenous attention. PLoS One, 4(3), e4869. 10.1371/journal.pone.0004869 19290043 PMC2653721

[IMAG.a.1347-b76] Slomian, J., Honvo, G., Emonts, P., Reginster, J.-Y., & Bruyère, O. (2019). Consequences of maternal postpartum depression: A systematic review of maternal and infant outcomes. Women’s Health, 15, 1745506519844044. 10.1177/1745506519844044 PMC6492376 31035856

[IMAG.a.1347-b77] Sotomayor-Enriquez, K., Gweon, H., Saxe, R., & Richardson, H. (2024). Open dataset of theory of mind reasoning in early to middle childhood. Data in Brief, 52, 109905. 10.1016/j.dib.2023.109905 38146306 PMC10749232

[IMAG.a.1347-b78] Spunt, R. P., Kemmerer, D., & Adolphs, R. (2016). The neural basis of conceptualizing the same action at different levels of abstraction. Social Cognitive and Affective Neuroscience, 11(7), 1141–1151. 10.1093/scan/nsv084 26117505 PMC4927039

[IMAG.a.1347-b79] Stipdonk, L. W., Franken, M.-C. J. P., & Dudink, J. (2018). Language outcome related to brain structures in school-aged preterm children: A systematic review. PLoS One, 13(6), e0196607. 10.1371/journal.pone.0196607 29864120 PMC5986152

[IMAG.a.1347-b80] Sullivan, G., Quigley, A. J., Choi, S., Teed, R., Cabez, M. B., Vaher, K., Corrigan, A., Stoye, D. Q., Thrippleton, M. J., & Bastin, M. (2024). Brain 3T magnetic resonance imaging in neonates: Features and incidental findings from a research cohort enriched for preterm birth. Archives of Disease in Childhood-Fetal and Neonatal Edition. https://fn.bmj.com/content/early/2024/07/02/archdischild-2024-326960.abstract 10.1136/archdischild-2024-326960 PMC11672019 38960453

[IMAG.a.1347-b81] Thompson, D. K., Matthews, L. G., Alexander, B., Lee, K. J., Kelly, C. E., Adamson, C. L., Hunt, R. W., Cheong, J. L. Y., Spencer-Smith, M., Neil, J. J., Seal, M. L., Inder, T. E., Doyle, L. W., & Anderson, P. J. (2020). Tracking regional brain growth up to age 13 in children born term and very preterm. Nature Communications, 11, 696. 10.1038/s41467-020-14334-9 PMC7000691 32019924

[IMAG.a.1347-b82] Treyvaud, K., Lee, K. J., Doyle, L. W., & Anderson, P. J. (2014). Very preterm birth influences parental mental health and family outcomes seven years after birth. The Journal of Pediatrics, 164(3), 515–521. 10.1016/j.jpeds.2013.11.001 24359937 PMC3950307

[IMAG.a.1347-b83] Valavani, E., Blesa, M., Galdi, P., Sullivan, G., Dean, B., Cruickshank, H., Sitko-Rudnicka, M., Bastin, M. E., Chin, R. F. M., MacIntyre, D. J., Fletcher-Watson, S., Boardman, J. P., & Tsanas, A. (2022). Language function following preterm birth: Prediction using machine learning. Pediatric Research, 92(2), Article 2. 10.1038/s41390-021-01779-x PMC8503721 34635792

[IMAG.a.1347-b84] van de Weijer-Bergsma, E., Wijnroks, L., & Jongmans, M. J. (2008). Attention development in infants and preschool children born preterm: A review. Infant Behavior and Development, 31(3), 333–351. 10.1016/j.infbeh.2007.12.003 18294695

[IMAG.a.1347-b85] van Noort-van der Spek, I. L., Franken, M.-C. J. P., & Weisglas-Kuperus, N. (2012). Language functions in preterm-born children: A systematic review and meta-analysis. Pediatrics, 129(4), 745–754. 10.1542/peds.2011-1728 22430458

[IMAG.a.1347-b86] Vandormael, C., Schoenhals, L., Hüppi, P. S., Filippa, M., & Borradori Tolsa, C. (2019). Language in preterm born children: Atypical development and effects of early interventions on neuroplasticity. Neural Plasticity, 2019(1), 6873270. 10.1155/2019/6873270 30930944 PMC6410465

[IMAG.a.1347-b87] Volpe, J. J. (2009). Brain injury in premature infants: A complex amalgam of destructive and developmental disturbances. The Lancet Neurology, 8(1), 110–124. 10.1016/S1474-4422(08)70294-1 19081519 PMC2707149

[IMAG.a.1347-b88] Waddington, C., van Veenendaal, N. R., O’Brien, K., & Patel, N. (2021). Family integrated care: Supporting parents as primary caregivers in the neonatal intensive care unit. Pediatric Investigation, 5(2), 148–154. 10.1002/ped4.12277 34179713 PMC8212757

[IMAG.a.1347-b89] Wellman, H. M. (2018). Theory of mind: The state of the art. European Journal of Developmental Psychology, 15(6), 728–755. 10.1080/17405629.2018.1435413

[IMAG.a.1347-b90] Wellman, H. M., Cross, D., & Watson, J. (2001). Meta-analysis of theory-of-mind development: The truth about false belief. Child Development, 72(3), 655–684. 10.1111/1467-8624.00304 11405571

[IMAG.a.1347-b91] Wellman, H. M., & Liu, D. (2004). Scaling of theory-of-mind tasks. Child Development, 75(2), 523–541. 10.1111/j.1467-8624.2004.00691.x 15056204

[IMAG.a.1347-b92] Wilson, S. M., Molnar-Szakacs, I., & Iacoboni, M. (2008). Beyond superior temporal cortex: Intersubject correlations in narrative speech comprehension. Cerebral Cortex, 18(1), 230–242. 10.1093/cercor/bhm049 17504783

[IMAG.a.1347-b93] Witt, S., Weitkämper, A., Neumann, H., Lücke, T., & Zmyj, N. (2018). Delayed theory of mind development in children born preterm: A longitudinal study. Early Human Development, 127, 85–89. 10.1016/j.earlhumdev.2018.10.005 30342224

[IMAG.a.1347-b94] Wolfe, K. R., Vannatta, K., Nelin, M. A., & Yeates, K. O. (2015). Executive functions, social information processing, and social adjustment in young children born with very low birth weight. Child Neuropsychology, 21(1), 41–54. 10.1080/09297049.2013.866217 24344821

