# Supplementary material for: Neural and behavioural correlates of theory of mind reasoning in five-year-old children born preterm
Source: Imaging Neurosci (Camb). 2026 Aug 26;4:IMAG.a.1347. doi: 10.1162/IMAG.a.1347 (PMC13519987; doi:10.1162/IMAG.a.1347)
Supplement: Supplementary file 1 [file IMAG.a.1347_supp.pdf]

Neural evidence of preserved Theory of Mind reasoning in five-year-old children born preterm: Supplementary Material

Supplement S1 2
Partly Cloudy Comprehension Measure 2
Figure S1 3
"Partly Cloudy" behavioural task performance 3
Supplement S2 3
Covariates 3
Supplement S3 5
Preparation and support for paediatric MRI 5
Supplement S4 5
fMRIPrep 24.0.0 "boiler plate" description 5
Figure S2 9
Table S1 10
Figure S3 12
Table S2 12
Figure S4 14
Supplement S5 15
Deviations from Preregistration 15
Supplement S6 15
False belief composite score analysis 15
Figure S5 16
Table S3 17
Supplement S7 18
Behavioural results in subset of children who completed fMRI (n = 76) 18
Table S4 19
Table S5 20
Supplement S8 21
Response Magnitude analyses of every timepoint across "Partly Cloudy" 21
Supplement S9 22
Testing for slower ToM neural responses among children born preterm 22
Supplement S10 23
Equivalence testing 23
Figure S5 27

Supplementary Material References..33

Supplement S1

Partly Cloudy Comprehension Measure

The “Partly Cloudy” behavioural task was developed to assess general movie comprehension as well as movie-specific ToM reasoning (https://osf.io/w9et8/overview?view_only=c8a2e1f1f191410a9f 70a757448d94f0). Because this was a novel task, we tested for a correlation between performance on the movie-specific ToM questions in this task and overall performance on the ToM booklet task. Performance on the ToM booklet task positively correlated with performance on the movie-specific ToM questions (β=0.07, t=3.33, p=0.001), controlling for test age (β=-0.01, t=-0.57, p=0.573; see Figure S1). When additionally controlling for receptive language (as both tasks have language demands), this correlation was not significant (β=0.03, t=1.74, p=0.087; effect of test age: β=0.02, t=0.91, p=0.364; effect of receptive language: β=0.09, t=4.52, p=2.56e-05).

Figure S1

“Partly Cloudy” behavioural task performance

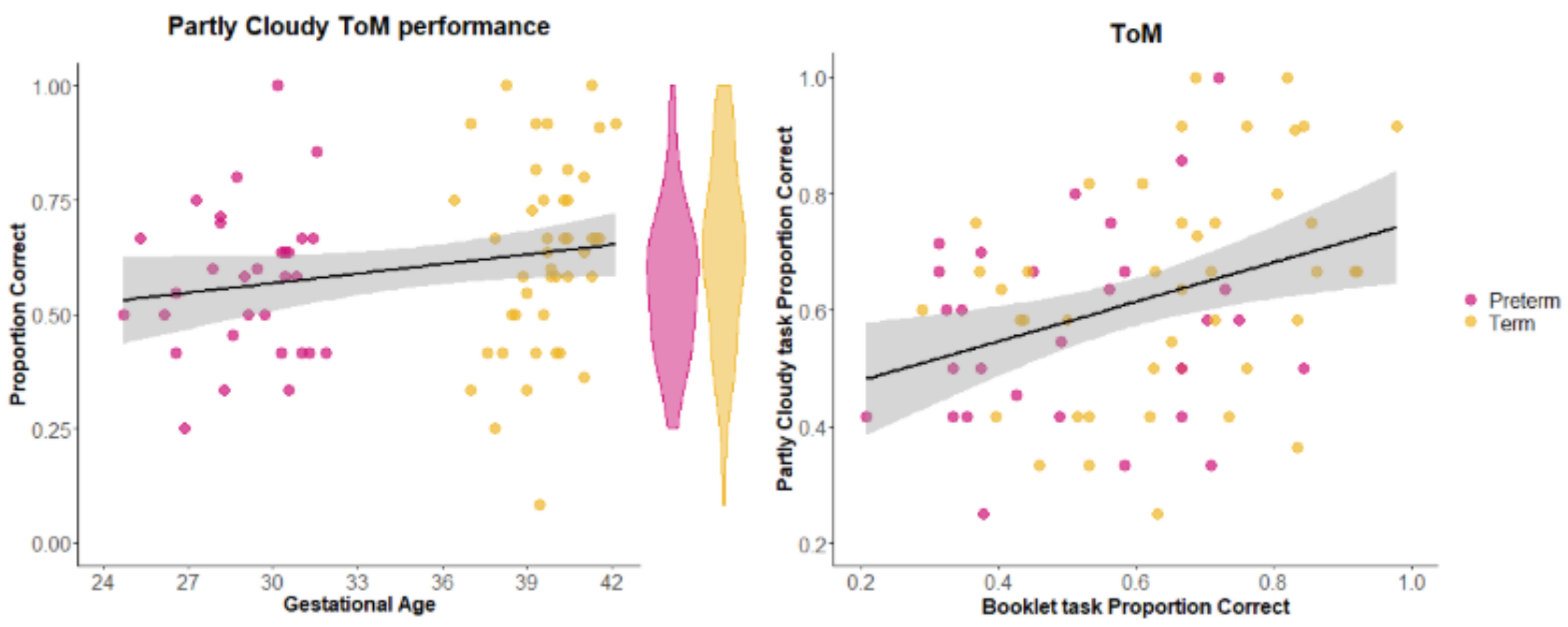

Figure S1. “Partly Cloudy” behavioural task performance. Scatterplots showing proportion correct on the 12 Theory of Mind (ToM) items of the “Partly Cloudy” task administered outside of the scanner (y-axes) by gestational age (GA; left plot, x-axis) and proportion correct on the 46-item ToM booklet task (right plot, x-axis). Violin plots to the right of each scatterplot illustrate the distribution of the measures by group: preterm-born children (*n*=30) are shown in magenta and term-born children (*n*=44) are shown in yellow.

Supplement S2

Covariates

TEBC participants complete a comprehensive behavioural battery at the five-year timepoint, enabling us to examine covariates that tend to correlate with ToM/GA. To measure language development, we used the receptive language subscale from the Mullen Scales of early learning (Mullen, 1995). To measure executive functions, and specifically response inhibition,

we calculated the average accuracy difference between prepotent and inhibition trials of the Early Child Inhibitory Touchscreen Task (ECITT; Holmboe et al., 2021). Finally, to measure attention, we used the "inattention" subscale ($n=9$ items) of the DuPaul ADHD Questionnaire (DuPaul et al., 2016), as completed by parents/ guardians.

Children born at a higher GA had higher receptive language scores on the Mullens, controlling for test age ($M(SE)$ in preterm-born children: 34.0(3.32); in term-born children: 48(3.53); effect of GA: $\beta=7.27$, $t=3.06$, $p=0.003$). There was no effect of GA on response inhibition (effect of GA: $\beta=0.02$, $t=1.04$, $p=0.302$; test age: $\beta=-0.009$, $t=-0.48$, $p=0.633$) or inattention (effect of GA: $\beta=-0.1$, $t=-0.19$, $p=0.847$; test age: $\beta=1.52$, $t=2.97$, $p=0.004$).

In (non-preregistered) analyses additionally controlling for executive functions and attention, the effect of GA on ToM performance (reported in the main text) was significant (effect of GA: $\beta=0.04$, $t=2.38$, $p=0.019$; receptive language: $\beta=0.09$, $t=5.47$, $p=3.61e-07$; inattention: $\beta=-0.003$, $t=-0.16$, $p=0.874$; inhibition: $\beta=0.01$, $t=0.84$, $p=0.404$; test age: $\beta=0.03$, $t=1.82$, $p=0.072$).

As children with more siblings pass false belief tasks earlier (Perner et al., 1994) and twins were more prevalent among children born preterm in the TEBC, we also compiled children's number of siblings (aged 1-12 years), as reported by parents/guardians. Preterm-born children had more siblings ($M(SD)$ in preterm-born children: 1.31(0.89); in term-born children: 0.98(0.56); effect of GA: $\beta=-0.19$, $t=-2.34$, $p=0.028$; effect of test age: $\beta=0.05$, $t=0.59$, $p=0.556$). However, this effect was driven by one set of preterm-born twins with four child-aged siblings. No other child in the sample had more than three siblings. In an analysis excluding these twins, there was no correlation between GA and number of siblings ($M(SD)$ in preterm-born children: 1.16(0.65); in term-born children: 0.98(0.56); effect of GA: $\beta=-0.12$, $t=-1.68$, $p=0.096$; test age: $\beta=0.01$, $t=0.13$, $p=0.897$) - as reported in the main text.

Supplement S3

Preparation and support for paediatric MRI

Children were provided with a booklet about the MRI scan and completed a mock scan prior to the MRI appointment. During the mock scan, the experimenters used the booklet to teach children about what getting an MRI scan would be like and to introduce them to the MRI facilities. Children would then practice lying still in a custom-built mock scanner while watching videos with scanner sounds playing in the background. If children moved during the mock scan, an experimenter tapped them on the leg to remind them to stay still. Immediately prior to the "real" scan, the booklet was reviewed and children practiced staying still and listened to recordings of the MRI sounds. During the scan, an experimenter at the entrance of the MRI bore (near the child's feet, the "scanner buddy") monitored the child throughout for comfort, alertness, and motion; a second experimenter monitored the child from the control room and spoke with them in between sequences (every 5-10 minutes). If/ when the child moved during the scan, the scanner buddy lightly tapped the child's leg, as a reminder to stay still. For a summary of considerations and preparatory procedures in paediatric MRI research see Dunstan et al. (2026).

Supplement S4

fMRIPrep 24.0.0 "boiler plate" description

The T1-weighted (T1w) image was corrected for intensity non-uniformity (INU) with N4BiasFieldCorrection (Tustison et al., 2010, distributed with ANTs 2.5.0 (Avants et al. 2008,

RRID:SCR_004757), and used as T1w-reference throughout the workflow. The T1w-reference was then skull-stripped with a Nipype implementation of the antsBrainExtraction.sh workflow (from ANTs), using OASIS30ANTs as target template. Brain tissue segmentation of cerebrospinal fluid (CSF), white-matter (WM) and gray-matter (GM) was performed on the brain-extracted T1w using fast (FSL, RRID:SCR_002823, Zhang, Brady, and Smith 2001). Brain surfaces were reconstructed using recon-all (FreeSurfer 7.3.2, RRID:SCR_001847, Dale, Fischl, and Sereno 1999), and the brain mask estimated previously was refined with a custom variation of the method to reconcile ANTs-derived and FreeSurfer-derived segmentations of the cortical gray-matter of Mindboggle (RRID:SCR_002438, Klein et al. 2017). A T2-weighted image was used to improve pial surface refinement. Brain surfaces were reconstructed using recon-all (FreeSurfer 7.3.2, RRID:SCR_001847, Dale, Fischl, and Sereno 1999), and the brain mask estimated previously was refined with a custom variation of the method to reconcile ANTs-derived and FreeSurfer-derived segmentations of the cortical gray-matter of Mindboggle (RRID:SCR_002438, Klein et al. 2017). Volume-based spatial normalization to one standard space (MNI152NLin2009cAsym) was performed through nonlinear registration with antsRegistration (ANTs 2.5.0), using brain-extracted versions of both T1w reference and the T1w template.

The following preprocessing was performed to the BOLD run for each subject. First, a reference volume and its skull-stripped version was generated using a custom methodology of fMRIPrep. Head-motion parameters with respect to the BOLD reference (transformation matrices, and six corresponding rotation and translation parameters) were estimated before any spatiotemporal filtering using mcflirt (FSL 6.0.5.1:57b01774, Jenkinson et al. 2002). A B0 nonuniformity map (or fieldmap) was estimated from the phasedifference map(s) measured using a dual-echoGRE (gradient-recalled echo) acquisition. The corresponding phase-map(s)

were phase-unwrapped with prelude (FSL). The estimated fieldmap was then aligned with rigid-registration to the target EPI (echo-planar imaging) reference run. The field map was mapped on to the reference EPI using the transform. The BOLD reference was then co-registered to the T1w reference using bbregister (FreeSurfer) which implements boundary-based registration (Greve and Fischl 2009). Co-registration was configured with six degrees of freedom. As is standard for fMRIPrep, several confounding time-series were calculated based on the preprocessed BOLD: framewise displacement (FD), DVARS and three region-wise global signals (from CSF, WM, and whole-brain masks). FD was computed using two formulations following Power (absolute sum of relative motions, Power et al. (2014)) and Jenkinson (relative root mean square displacement between affines, Jenkinson et al. (2002)), using their implementations in Nipype (following the definitions by Power et al. 2014). Additionally, a set of physiological regressors were extracted to allow foranatomy-constrained component-based noise correction (aCompCor, Behzadi et al. 2007). Principal components are estimated after high-pass filtering the preprocessed BOLD time-series (using a discrete cosine filter with 128s cut-off). For aCompCor, three probabilistic masks (CSF, WM and combined CSF+WM) are generated in anatomical space. A mask of pixels that likely contain a volume fraction of GM is subtracted from the aCompCor masks. This mask is obtained by dilating a GM mask extracted from the FreeSurfer's aseg segmentation, and it ensures components are not extracted from voxels containing a minimal fraction of GM. Finally, these masks are resampled into BOLD space and binarized by thresholding at 0.99 (as in the original Behzadi et al., 2007b implementation). Components are also calculated separately within the WM and CSF masks. The head-motion estimates calculated in the correction step were also placed within the corresponding confounds file. Frames that exceeded a threshold of 1.0 mm FD or 1.5 standardized DVARS were annotated as motion

outliers. Additional nuisance timeseries are calculated by means of principal components analysis of the signal found within a thin band (crown) of voxels around the edge of the brain, as proposed by (Patriat, Reynolds, and Birn 2017). The BOLD time-series were resampled into standard space, generating a preprocessed BOLD run in MNI152NLin2009cAsym space. First, a reference volume and its skull-stripped version will be generated using a custom methodology of fMRIPrep. All resamplings can be performed with a single interpolation step by composing all the pertinent transformations (i.e. head-motion transform matrices, susceptibility distortion correction when available, and co-registrations to anatomical and output spaces). Gridded (volumetric) resamplings were performed using nitransforms, configured with cubic B-spline interpolation.

Many internal operations of fMRIPrep use Nilearn 0.10.4 (Abraham et al. 2014, RRID:SCR_001362), mostly within the functional processing workflow. For more details of the pipeline, see the section corresponding to workflows in fMRIPrep's documentation. Note that this description is a lightly edited version of the automatically-generated output of fMRIprep; this text is released for use under the CC0 license.

Figure S2

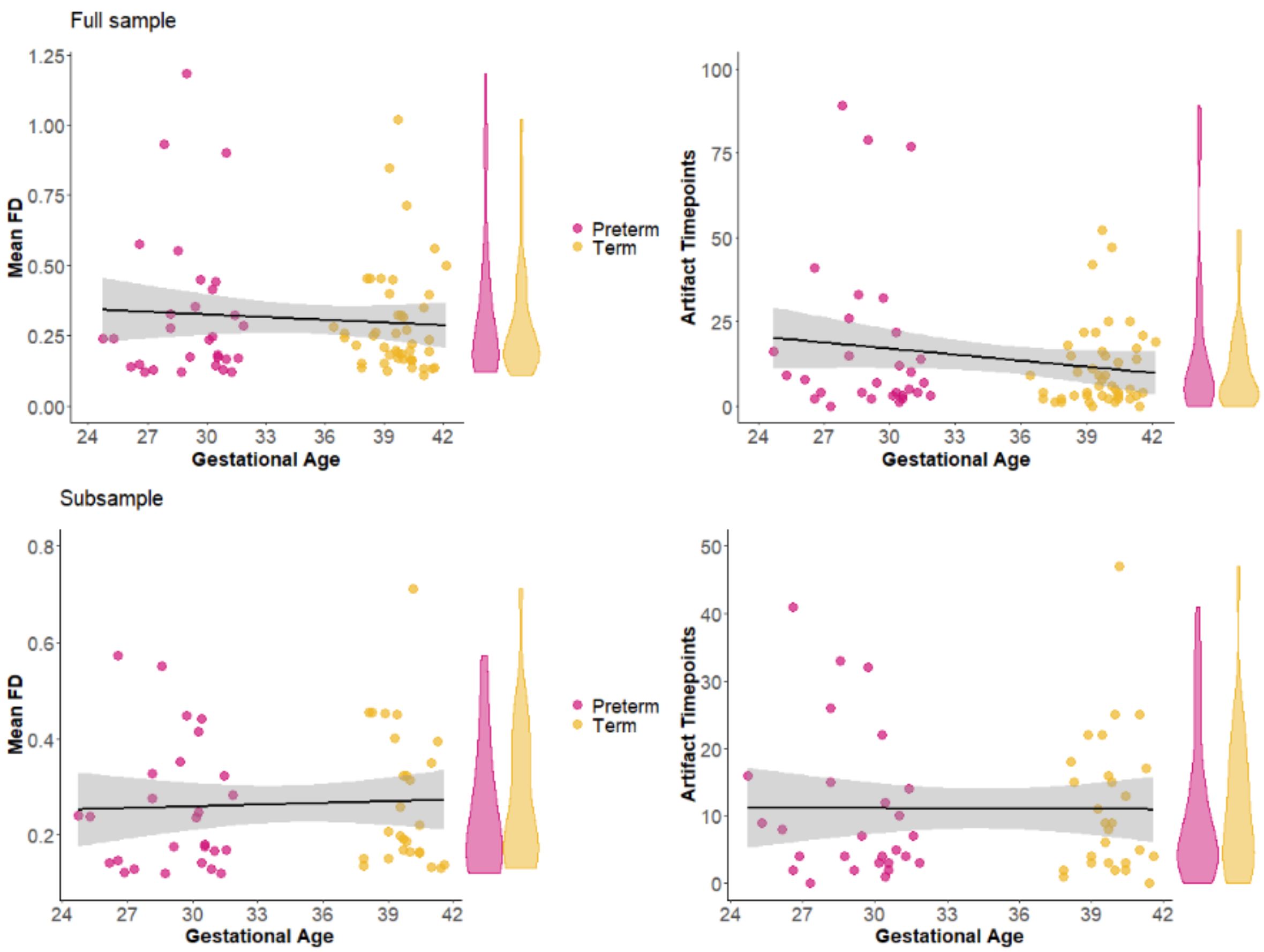

Figure S2. FMRI data quality and gestational age. Scatterplots show the amount of motion (y-axes, mean framewise displacement (FD) on the left, number of artifact timepoints on the right) per participant by GA (x-axes). Violin plots show the distribution of values per group. Children born preterm are shown in magenta, children born at term are shown in yellow. The top panel shows the full sample (*n*=30 children born preterm, *n*=46 children born at term); the bottom panel shows the motion-matched subsample (*n*=27 children per group).

Table S1

Analysis	Level	Description
First-level modelling	Whole-brain	For each participant, BOLD was modelled as a function of event type (Mind, Body) using a general linear model. Data were modelled using boxcar regressors convolved with a canonical hemodynamic response function (HRF). Nuisance regressors for artifact timepoints and five aCompCor regressors were included in the model. Data were high-pass filtered (0.01Hz).<br>*Note: Outputs were only carried forward into second-level whole brain random effects analysis, not used in statistical analysis.*
Whole-brain Random Effects Analyses	Whole-brain	Standard RFX analysis, conducted across the whole brain.<br>Conducted in (1) preterm children, (2) term-born children, and (3) across all children, with gestational age as a covariate of interest.<br>All analyses examined the Mind > Body contrast, which used 7 mind and 9 body "events" defined in prior research (Richardson et al., 2018 and https://openneuro.org/datasets/ds000228).
ROI Analyses		

Functional maturity*	Timecourse, per ROI	"Functional maturity" is calculated as the correlation between each child's timecourse and an average adult timecourse.
Response magnitude to mind events*	Timecourse, per ROI	Extracted response magnitude to three events (timepoints) defined in prior research (Richardson et al., 2018).<br>*Note: Exploratory analyses were conducted across the full timecourse, to ensure we did not miss effects of gestational age where present.*
Inter-region correlations	Timecourse, per ROI	Inter-region correlations were calculated using the full timecourse extracted from ToM and Pain Matrix ROIs. We then calculated the average inter-region correlation within the ToM network and across the ToM network and Pain Matrix, per participant.
Inter-subject correlations*	Timecourse, per ROI	Inter-subject correlations were calculated using the full timecourse, per ToM ROI. We then calculated the average within- vs. across- group correlations per participant.

Table S1. Brief description of analyses and metrics. Note, that all metrics are described in the Methods section. * indicates metrics were calculated per ToM ROI. As described in section 2.4.3 Regions of interest (ROI) analyses, we then conduct statistics (1) on RTPJ specifically and (2) across the full ToM network. For the latter, we include metrics from all ROIs and test for an effect/ interactions with ROI.

Figure S3

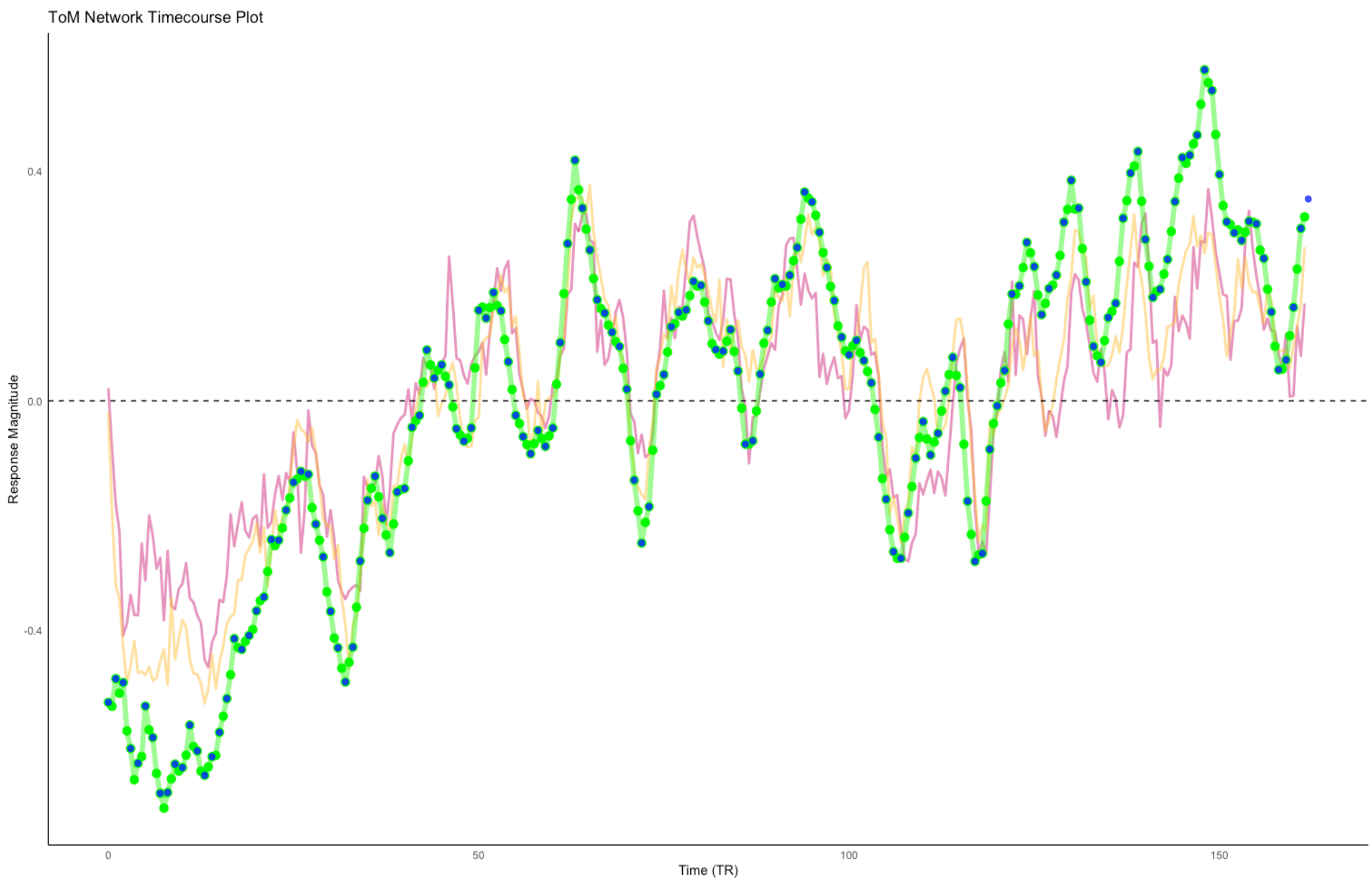

Figure S3. Interpolated and original timecourses. Timecourses show the average response magnitude (y-axis) across time (in seconds, x-axis) in the ToM network. For the MIT sample (5-year-olds), acquired datapoints (at TR=2) are shown in blue and interpolated datapoints (to ‘upsample’ to TR=1) are shown in green. TEBC 5-year-olds (preterm in magenta, n = 30; term in yellow, n = 46; TR=1) are shown for context.

Table S2

Event label	Timepoints (TR=1)	Peak timepoint Richardson et al., 2018 (TR=2)	Description

Event 1 (T02)	192-193	96	Peck longingly stares at one of the other clouds making lovely puppies. Gus notices this and his facial expression changes from happy to more worried/insecure. Peck realises that he has been caught and looks ashamed.
Event 2 (T01)	262-263	131	Peck the stork flies away from Gus the cloud after seeing the baby shark Gus has made. He lands on another cloud that was previously shown making puppies. Peck and the other cloud laugh together.
Event 3 (T04)	300-301	150	Peck reveals protective football gear and puts it on, making Gus realize that Peck wasn't abandoning him, but that he asked the other cloud for the gear to be able to continue working with Gus.

Table S2. "Mind" event details. Event labels, timepoints (in TR), and descriptions. Event timepoints and T0# labels were taken from Richardson et al. (2018) and adjusted to accommodate the TR difference between fMRI sequences. The T0# event labels reflect ordering in terms of their response magnitude values in adults (as originally observed in Richardson et al., 2018), rather than by time.

187

Figure S4

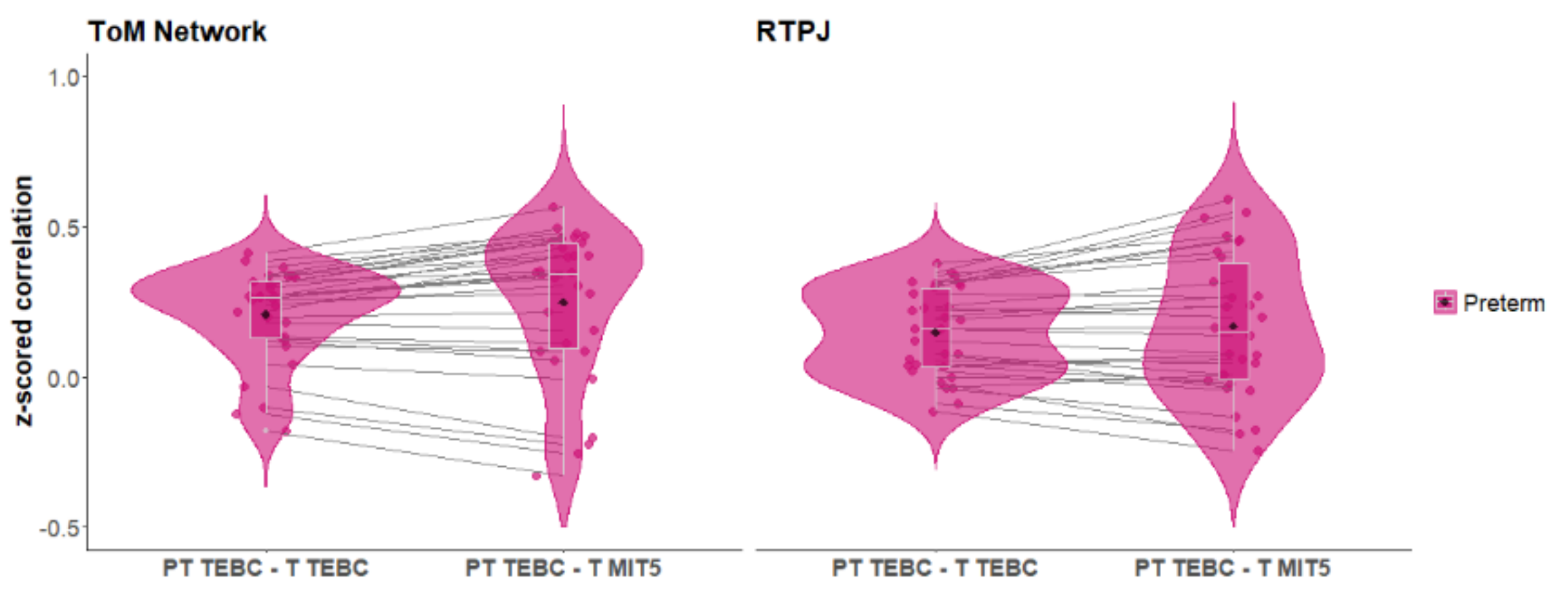

Figure S4. Intersubject correlation analysis site effects. Box and violin plots show the z-scored correlation values (y-axes) for intersubject comparisons (x-axes) of the across TEBC preterm- (*n*=30) to term-born (*n*=46) five-year-olds ("PT TEBC – T TEBC") and the across TEBC preterm- to term-born (*n*=31) five-year-olds from the publicly available dataset ("PT TEBC – T MIT5"). Results for the ToM network are on the left, for the RTPJ on the right. The horizontal line in the boxplot shows the median, the lower and upper hinges of the boxes correspond to the first and third quartiles, with the upper whisker extending to the largest value no further than 1.5 * inter-quartile range (IQR) from the hinge and the lower whisker extending from the lower hinge to the smallest value at most 1.5 * IQR of the hinge. Black diamonds indicate the group mean. Statistical tests showed lower intersubject correlations between preterm to TEBC term-born children relative to term-born children acquired at MIT in the ToM network ($t(29)=-2.26$, $p=0.031$, two-tailed); this effect was not significant in RTPJ ($t(29)=-1.10$, $p=0.280$, two-tailed).

Supplement S5

Deviations from Preregistration

Behavioural (https://osf.io/97tu6) and fMRI (https://osf.io/wjhdb) analyses were preregistered to the Open Science Framework; deviations were minimal. First, we accidentally omitted the GA-by-ROI interaction term in linear mixed effect models predicting ToM network functional maturity and response magnitude in the preregistered analysis plan. We included this interaction term in analyses. As preregistered, non-significant interaction terms were omitted from final regressions. Second, in intersubject correlation analyses testing whether responses among preterm-born children were more variable than term-born children, we articulated our hypothesis as intended but specified the wrong direction of the effect in the statistical test notation (i.e., by indicating “greater”, rather than “lesser” in the R syntax). We therefore tested the preregistered hypothesis using the correct syntax.

Supplement S6

False belief composite score analysis

We repeated analyses specifically with a false belief composite score (14 items) and found the same pattern of results: children born at a later GA performed better on false belief items when controlling for age only (effect of GA: β=0.09 t=3.29, p=0.001; test age: β=-0.01, t=-0.43, p=0.67), and when additionally controlling for receptive language (effect of GA: β=0.05, t=2.04, p=0.044; receptive language: β=0.11, t=4.08, p=8.66e-05; test age: β=0.01, t=0.47, p=0.643).

Figure S5

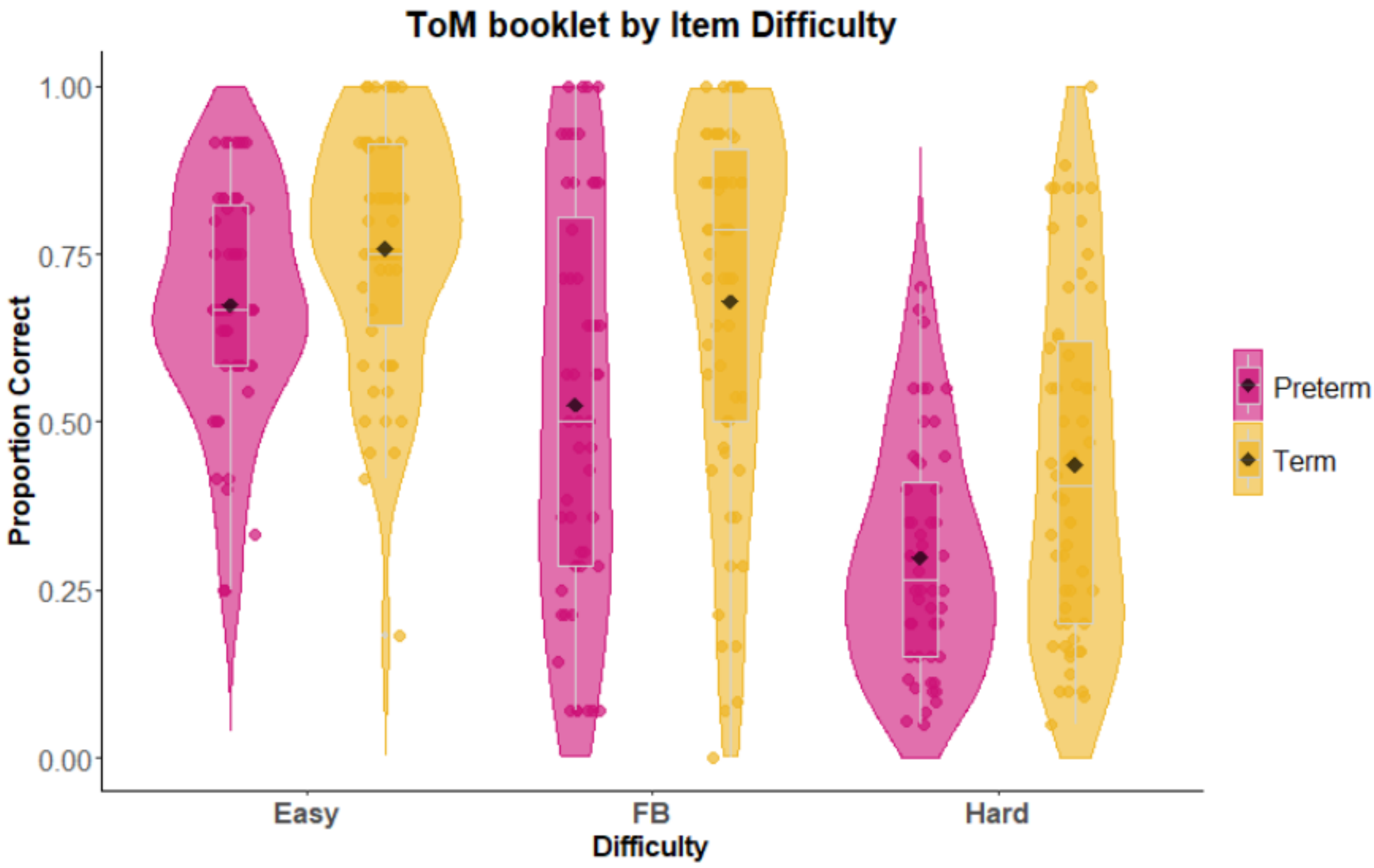

Figure S5: ToM booklet by Item Difficulty. Violin plot showing proportion correct on the ToM booklet test questions (y-axis) by item difficulty (left: easy items, middle: false belief items, right: hard items; x-axis) and illustrate the distribution of the measures by group: preterm-born children (*n*=52) are shown in magenta and term-born children (*n*=58) are shown in yellow. Black diamonds indicate group means. The horizontal line in the boxplot shows the median, the lower and upper hinges of the boxes correspond to the first and third quartiles, with the upper whisker extending to the largest value no further than 1.5 * inter-quartile range (IQR) from the hinge and the lower whisker extending from the lower hinge to the smallest value at most 1.5 * IQR of the hinge. There was no GA-by-item difficulty interaction effect, so the interaction term was removed from the final model. There were main effects of GA (β=0.04, t=2.78, p=0.006), receptive language (β=0.09, t=5.40, p=4.27e-07) and difficulty level (indicating false belief and hard items were more difficult, overall, than easy

items; false belief: *β*=-0.11, *t*=-4.53, *p*=9.75e-06; hard: *β*=-0.35, *t*=-14.38, *p*< 2e-16) on ToM performance; the main effect of test age was not significant (*β*=0.02, *t*=1.32, *p*=0.190).

Table S3

Behavioural Measure	Predictors	Full Sample		
ToM Performance	GA	*β*=0.12	*z*=1.413	*p*=0.158
	Response Type (Free Response)	***β*=-0.83**	***z*=-5.85**	***p*=5.01e-09**
	Difficulty (Hard)	***β*=-1.72**	***z*=-15.46**	***p*<2e-16**
	Difficulty (FB)	***β*=-0.83**	***z*=-6,18**	***p*=6.30e-10**
	Age	*β*=0.13	*z*=1.53	*p*=0.126
	Receptive Language	***β*=0.01**	***z*=5.64**	***p*=1.72e-08**
	GA*Response Type (Free Response)	***β*=0.27**	***z*=3.97**	***p*=7.25e-0.5**
	Response Type (Free Response)* Difficulty (Hard)	*β*=0.20	*z*=1.18	*p*=0.239
	Response Type (Free Response)* Difficulty (FB)	***β*=0.60**	***z*=3.27**	***p*=0.001**

Table S3: Full statistical results for mixed effects logistic regression simultaneously testing for effects of GA, response type (2AFC vs free response), and ToM concept difficulty (easy vs false belief vs hard) on behavioural ToM performance. Statistical results for the analysis of ToM performance in the full sample (*n*=52 preterm-, *n*=58 term-born children). The table shows standardised betas (*β*), z-values (*z*), and p-values (*p*) for each predictor in the model. P-values below *p*=0.05 shown in bold. 2AFC was set as the reference Response Type, Easy as

reference concept difficulty level. Inclusion of the interaction terms improved the model fit, as evidenced by a lower AIC value.

Supplement S7

Behavioural results in subset of children who completed fMRI (n = 76)

In the subsample of children who completed fMRI and behavioural testing (preterm: n=28, term: n=46), GA had a positive effect on behavioural ToM booklet task performance, controlling for test age (effect of GA: β=0.07, t=3.55, p=0.001; test age: β=0.009, t=0.4, p=0.688; M(SE) proportion correct in preterm-born children: 0.51(0.03), term-born children: 0.64(0.03)). The effect of GA on ToM performance remained significant when controlling for receptive language (effect of GA: β=0.04, t=2.29, p=0.03; receptive language: β=0.1, t=4.9, p=6.17e-06; test age: β =0.03, t=1.7, p=0.09).

Additional analysis of the positive effect of GA on ToM performance among only the preterm-born children showed that, in this subsample of children who completed fMRI and behavioural tasks, this effect was not "dose-dependent" - i.e., within the preterm-born group (n=28), those born at higher GA did not perform better on the ToM task, controlling for test age and receptive language (β=0.05, t=1.81, p=0.084).

In a mixed-effects logistic regression testing for effects of GA alongside response type, difficulty, age, and receptive language (and interaction effects), there was a significant GA-by-response type interaction effect (the positive effect of GA was stronger for free response items) and significant main effects of response type (indicating free response items were

more difficult than 2AFC items), difficulty, and receptive language; the effect of GA was not significant (see Table S4 for full statistical results).

Table S4

Behavioural Measure	Predictors	Full Sample		
ToM Performance	GA	β=0.17	z=1.55	p=0.121
	Response Type (Free Response)	**β=-0.68**	**z=-3.88**	**P<0.001**
	Difficulty (Hard)	**β=-1.63**	**z=-11.97**	**p<2e-16**
	Difficulty (FB)	**β=-0.61**	**z=--4.18**	**p=2.92e-05**
	Age	β=0.19	z=1.87	p=0.062
	Receptive Language	**β=0.02**	**z=4.93**	**p=8.30e-07**
	GA*Response Type (Free Response)	**β=0.18**	**z=2.14**	**p=0.033**
	Response Type (Free Response)* Difficulty (Hard)	β=0.22	z=1.05	p=0.239
	Response Type (Free Response)* Difficulty (FB)	**β=0.56**	**z=2.47**	**p=0.013**

Table S4: Full statistical results for mixed effects logistic regression simultaneously testing for effects of GA, response type (2AFC vs free response), and ToM concept difficulty (easy vs false belief vs hard) on behavioural ToM performance. Statistical results for the analysis of ToM performance in children who completed the fMRI (n=28 preterm-, n=46 term-born children). The table shows standardised betas (β), z-values (z), and p-values (p) for each predictor in the model. P-values below p=0.05 shown in bold. 2AFC was set as the reference

Response Type, Easy as reference concept difficulty level. Inclusion of the interaction terms improved the model fit, as evidenced by a lower AIC value.

Table S5

Neural Measure	Predictors	Full Sample			Motion matched Sample		
RM event 1 (T02): ToM Network	GA	**β= 0.20**	**t= 3.19**	**p= 0.002**	**β= 0.20**	**t= 3.19**	**p= 0.002**
	Age	β= 0.05	t= 1.14	p= 0.259	β= 0.05	t= 1.14	p= 0.259
	ROI: DMPFC	**β= -0.22**	**t= -2.96**	**p= 0.003**	**β= -0.22**	**t= -2.96**	**p= 0.003**
	ROI: LTPJ	β= 0.02	t= 0.23	p= 0.822	β= 0.02	t= 0.23	p= 0.822
	ROI: MMPFC	**β= -0.21**	**t= -2.89**	**p= 0.004**	**β= -0.21**	**t= -2.89**	**p= 0.004**
	ROI: PC	β= 0.14	t= 1.86	p= 0.064	β= 0.14	t= 1.86	p= 0.064
	ROI: VMPFC	**β= -0.25**	**t= -3.37**	**p= 0.001**	**β= -0.25**	**t= -3.37**	**p= 0.001**
	Motion	β= -0.01	t= -0.27	p= 0.791	β= -0.01	t= -0.27	p= 0.791
	GA * ROI: DMPFC	**β= -0.19**	**t= -2.64**	**p= 0.009**	**β= -0.19**	**t= -2.64**	**p= 0.009**
	GA * ROI: LTPJ	β= -0.11	t= -1.45	p= 0.149	β= -0.11	t= -1.45	p= 0.149
	GA * ROI: MMPFC	**β= -0.20**	**t= -2.68**	**p= 0.008**	**β= -0.20**	**t= -2.68**	**p= 0.008**

	GA * ROI: PC	*β*= -0.06	*t*= -0.86	*p*= 0.393	*β*= -0.06	*t*= -0.86	*p*= 0.393
	GA * ROI: VMPFC	*β*= -0.10	*t*= -1.41	*p*= 0.160	*β*= -0.10	*t*= -1.41	*p*= 0.160

Table S: Full statistical results for Response Magnitude Analysis: Event 1 (T02) in the ToM Network. Statistical results for the analysis of the ToM network response magnitude to event 1 (T02) for the full sample (*n*=30 preterm, *n*=46 term-born children) and the motion matched sub-sample (*n*=27 children each born preterm and term). Showing standardised betas (*β*), t-values (*t*), and p-values (*p*) for each predictor in the linear mixed model regression. P-values below *p*=0.05 shown in bold. RTPJ was set as the reference ROI, so significant interaction terms reflect a larger effect of GA on response magnitude in RTPJ relative to DMPFC and MMPFC. Significant main effects of DMPFC, MMPFC, and VMPFC reflect smaller responses in those ROIs, relative to RTPJ, to event 1 (T02).

Supplement S8

Response Magnitude analyses of every timepoint across "Partly Cloudy"

To assess whether differences in response magnitude to mind event 1 (T02) reflected broader differences between preterm- and term-born children, and to ensure differences in responses at other timepoints during the movie didn't go undetected, we repeated the response magnitude analysis for every timepoint of the movie/response timecourse. In line with prior research and our main analyses, "Events" were defined as four consecutive timepoints (i.e., 4 seconds of the movie) - to protect against false positives and account for multiple comparisons. This analysis revealed one event, from timepoint 190-195, during which

response magnitude positively correlated with GA; this event includes event 1 (T02) (timepoints 192-193).

Supplement S9

Testing for slower ToM neural responses among children born preterm

Given diffuse white matter differences among children born preterm (Deferm et al., 2024), one hypothesis is that ToM brain regions in children born preterm would be slower to respond to the movie, relative to term-born peers. In non-preregistered intersubject correlation analyses, we tested this hypothesis. If preterm-born children's neural responses to the movie were delayed (i.e., slower) relative to their term-born peers, then intersubject correlation values should be higher when correlating timecourses of term and preterm-born children with a small time-shift, such that later timepoints among preterm-born children correspond to earlier timepoints among term-born children.

We therefore conducted two additional intersubject correlation analyses: First, we correlated every term-born child's timecourse excluding the *last* two seconds to every preterm-born child's timecourse excluding the *first* two seconds ("delayed response"; i.e., seconds 0-321 in term-born to seconds 2-323 in preterm-born children). Second, we correlated every term-born child's timecourse excluding the last two seconds to every preterm-born child's timecourse excluding the last two seconds ("no delay"; i.e., seconds 0-321 in all children). We then conducted a one-tailed t-test on z-scored correlation values to test whether intersubject correlations were higher in the "delayed response" scheme. We conducted intersubject

correlation analyses and t-tests for the ToM network average response and RTPJ response timecourses, separately.

Intersubject correlations were higher in the no delay scheme (*M*(*SE*) ToM network – across preterm-term correlations with no delay: 0.21(0.03), across preterm-term correlations delayed response: 0.20(0.03), 2-tailed t-test: $t(29)=3.38$, $p=0.002$; RTPJ - across preterm-term no delay: 0.15(0.03), across preterm-term delayed response: 0.14(0.03), 2-tailed t-test: $t(29)=2.79$, $p=0.009$). These results suggest that ToM brain regions were not slower to respond to the movie among children born preterm.

Supplement S10

Equivalence testing

Methods

While our pre-registered statistical analyses are suited to test for the presence of significant effects of gestational age, in cases where there was no significant effect of gestational age, these analyses cannot statistically reject the presence of an effect of gestational age. To statistically reject the presence of an effect of gestational age on our neural ToM metrics, we took two approaches.

First, we used iterative two one-sided test (TOST) equivalence tests (Lakens et al., 2018) using a range of equivalence bounds (i.e., bounds that reflect how small an effect would have to be to be of no interest / indicate equivalence). Specifically, for most neural metrics we tested for standardised beta (β) effect sizes of gestational age within the range of -0.2 to 0.2, in 0.01 increments. For the response magnitude to event 1 (T02), where our pre-registered analyses

indicated a significant effect of gestational age, and testing for effect sizes of age on all neural metrics we extended this range to -0.35 to 0.35. The models for each neural metric used in these analyses were the same as in our preregistered analyses; that is, generalised linear fixed and mixed effects regression models testing for effects of GA (continuous variable, modelled across the full GA range) on the neural measures, controlling for test age and motion. For ROI specific measures (functional maturity, response magnitude), we used mixed effects linear regressions with ROI as a fixed effect and subject as random effect, where GA-by-ROI interaction terms were included only when significant (ToM network event 1 (T02) model, only). All predictors were standardised; the dependent variable was not. This facilitates interpreting results: we can then say that for every standard deviation change in gestational age (i.e., ~5.5 weeks), the neural metric changes by its standardised beta value. For these analyses, we report the narrowest equivalence bounds (expressed as standardised beta values) at which we can statistically reject the presence of an effect of gestational age ($p<0.05$). In Figure S5, we visualise a density plot which shows the likely size of the effect of gestational age (dashed vertical line) alongside the tested equivalence bounds (plotted along the x-axis, the full tested range is -0.02 to 0,2, with -0.15 to 0.15 represented by a different colour every 0.01; the narrowest equivalence bounds at which we can statistically reject the presence of an effect of gestational age or age are highlighted in vertical hot pink bars).

Second, we used a Bayesian region of practical equivalence (ROPE) test (Kruschke, 2018) and report the beta values inside of which at least 95% of the posterior distribution falls. For these analyses, the fixed effects prior was set to a normal distribution with a mean of 0 and standard deviation of 1. Sensitivity analyses were conducted to ensure an appropriate prior was used as there was no perfect precedent from prior research. In these analyses, we used a range of priors for the effect of gestational age (from 0.25-1, and compared to a flat prior) and found

that results were robust to prior choice. Subject was included as a random effect for models with multiple regions of interest (testing effects within the full ToM network); the default flat prior was used for this random effect.

For both approaches, we report and visualise results for effects of gestational age (range: 24-42.1 weeks; Figure S5, left column) alongside results for effects of test age (range: 5.02-5.86; Figure S5, right column) for each neural ToM metric; gestational age and test age were weakly negatively correlated in the fMRI sample ($r(74) = -0.14$). Reporting results for age allows us to contextualise the results for gestational age and determine if any metrics indicate effects similar to or larger than the effect of age among five-year-olds, within a 10-month range. This is a practically useful comparison: it enables us to consider whether effects of gestational age are larger than those observed across individual five-year-old children within a shared classroom, for example.

Results

Full results and visualisations, per neural metric, are provided in Figure S5; the figure legend also provides a detailed example for how to interpret the figure. Briefly, for all neural metrics where there was no statistical effect of gestational age in our pre-registered analyses (within-ToM- and across-ToM-Pain inter region correlations, functional maturation index, and response magnitudes to event 2 (T01) and event 3 (T04) in the full ToM network and RTPJ), using TOSTs, we could statistically reject the presence of an effect of gestational age (i.e., infer statistical equivalence) at beta values >0.2. Evidence was particularly strong for the across ToM-Pain inter-region correlation metric, the ToM network functional maturation metric, and the within-ToM network inter-region correlation metric, where there was evidence for statistical equivalence at standardised beta values <0.1. For all neural metrics except the

within-ToM network inter-region correlation, the results suggest that any effects of gestational age (Figure S5, left column) would be smaller than effects of test age (Figure S5, right column); that is, the effects of one standard deviation change in gestational age (approximately 5 weeks; one-third of the range of values) would be smaller than those of one standard deviation change in test age (approximately 1.5 months).

Consistent with the results of our pre-registered analyses, effects of gestational age on the response magnitude in the full ToM network and in RTPJ specifically to event 1 (T02) were larger than +/- 0.2, that is larger than the equivalence bounds observed for all other metrics, and larger than the effects of age on the response magnitude to event 1 (T02) in the full ToM network and in RTPJ, respectively.

The results of the ROPE analyses offer further evidence consistent with the TOSTs. Specifically, for each neural metric (apart from ToM network and RTPJ responses to event 1 (T02)), at least 95% of the density plot fell within the significant equivalence bounds derived from the TOSTs. See Figure S5 for ROPE bounds and density plot percentages.

Figure S5

GA (range: 24-42.1 weeks)	Age (range: 5.02-5.86 years)

Across ToM-Pain IRC

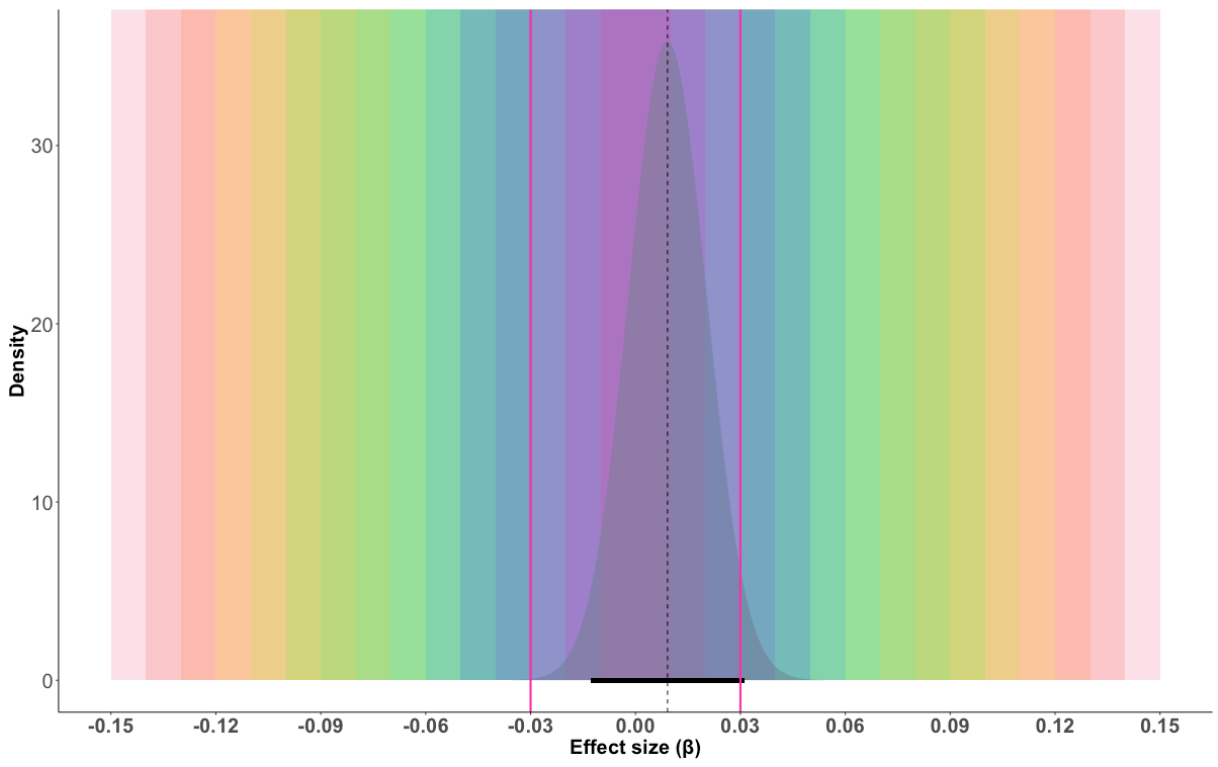

TOST: β = +/- 0.03, p(equivalence) = 0.031

ROPE: β = +/- 0.03, 100%

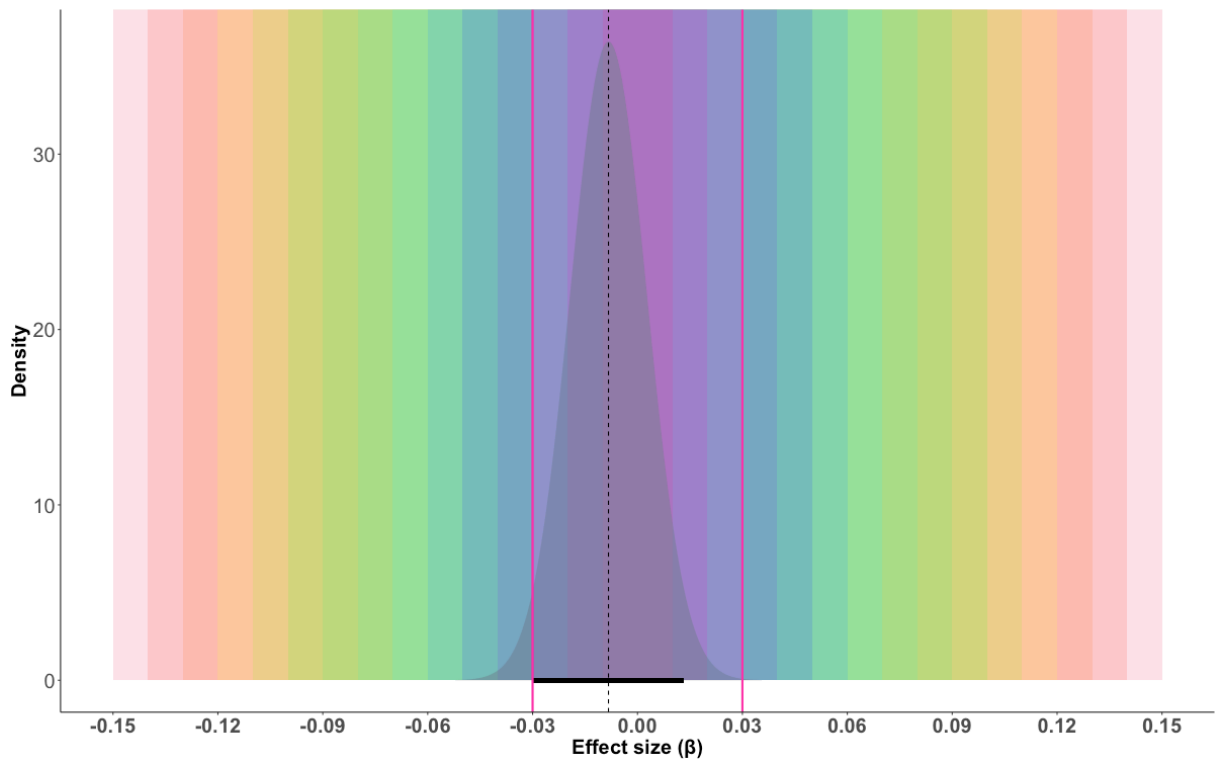

TOST: β = +/- 0.03, p(equivalence) = 0.024

ROPE: β = +/- 0.03, 100%

ToM Network FuncMat

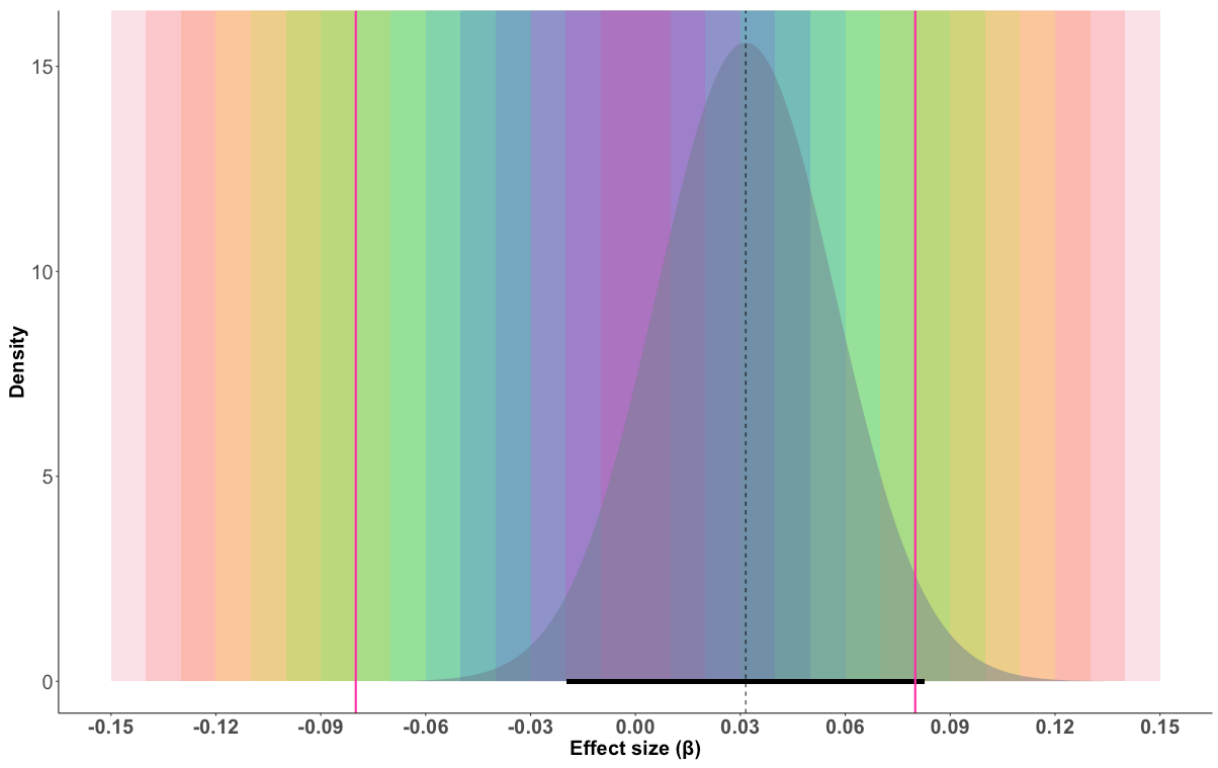

TOST: β = +/- 0.08, p(equivalence) = 0.031

ROPE: β = +/-0.07, 95%

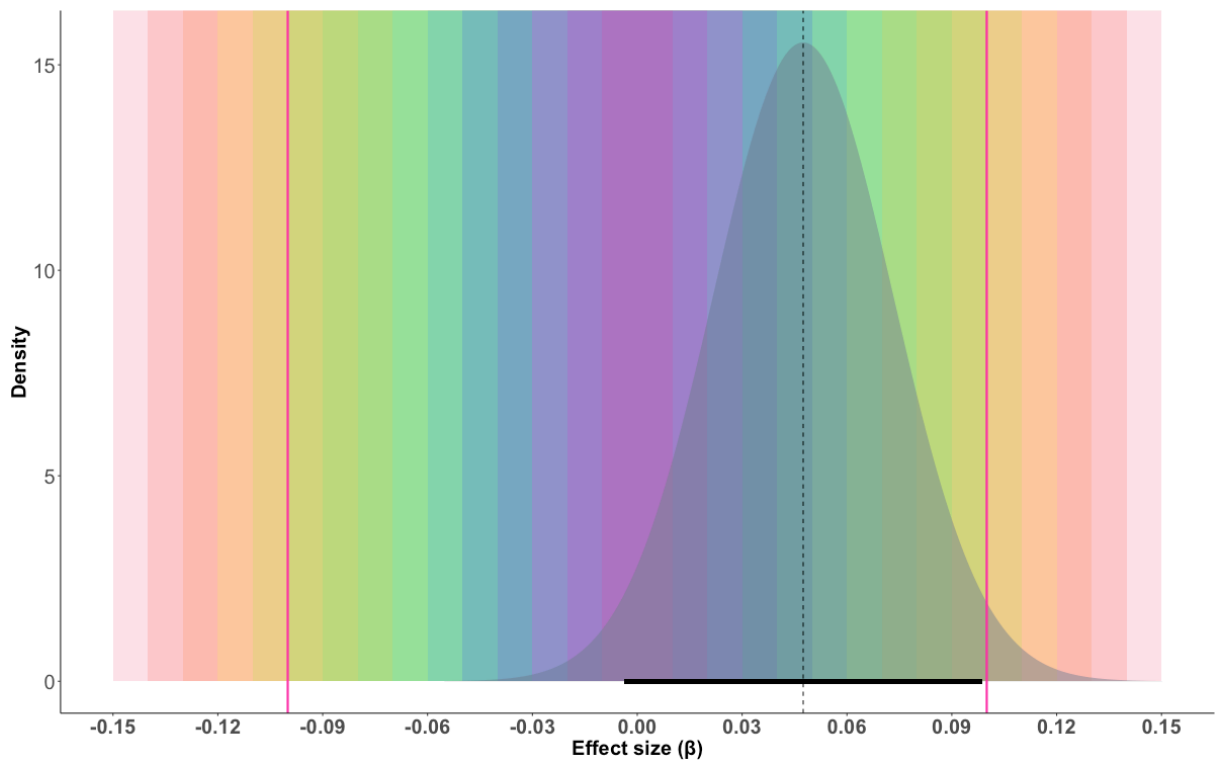

TOST: β = +/- 0.1, p(equivalence) = 0.022

ROPE: β = +/- 0.09, 97%

Within-ToM IRC

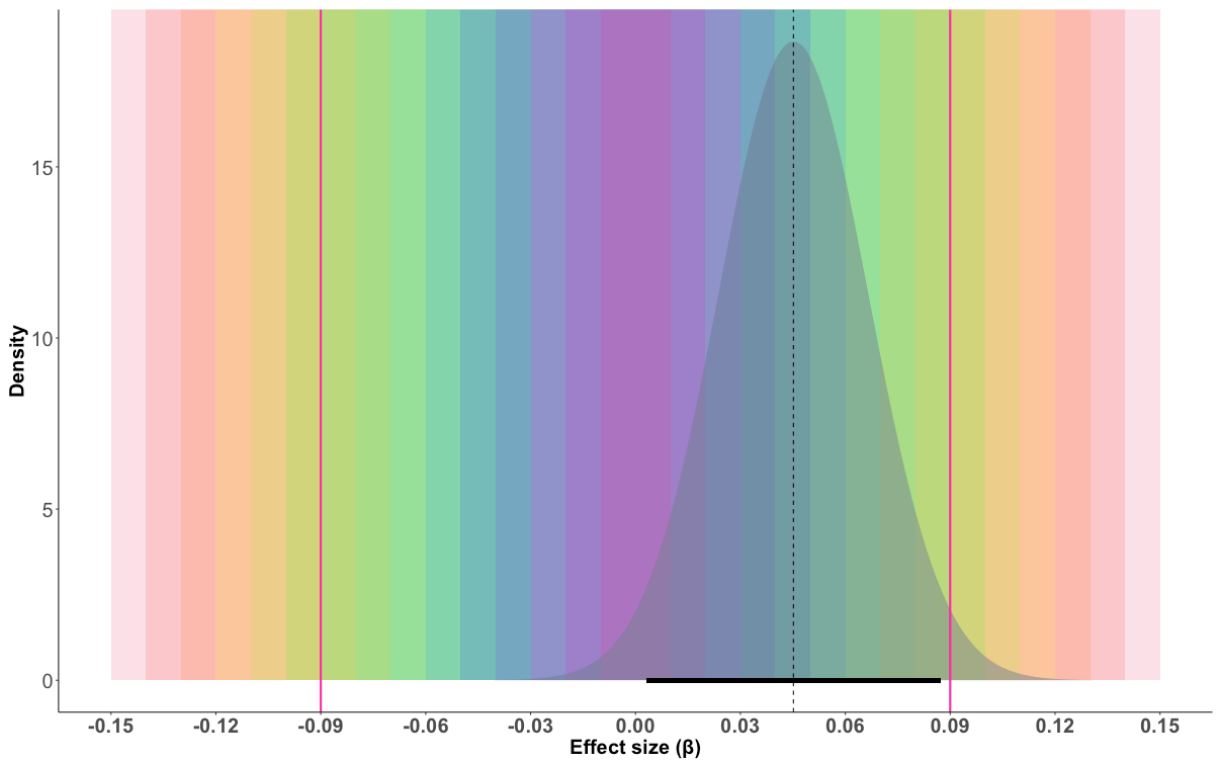

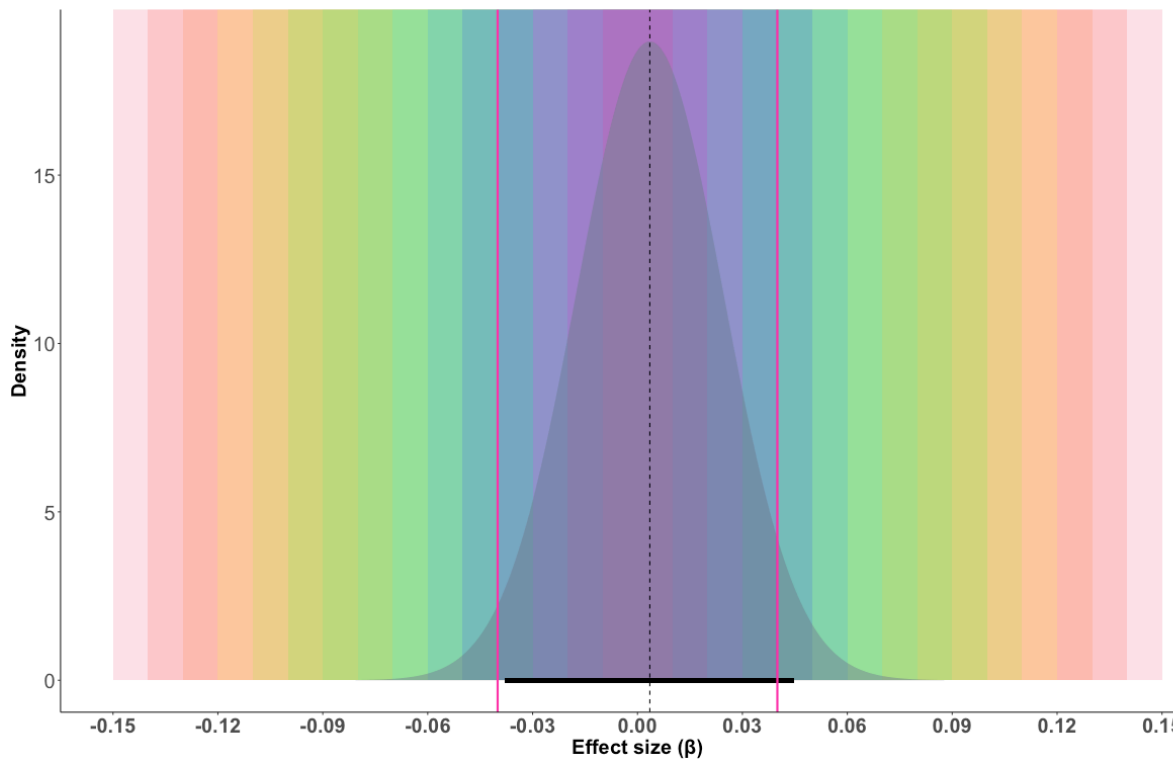

TOST: β = +/- 0.09, p(equivalence) = 0.018

ROPE: β = +/- 0.08, 98%

TOST: β = +/- 0.04, p(equivalence) = 0.041

ROPE: β = +/- 0.04, 100%

ToM Network Event 3(T04)

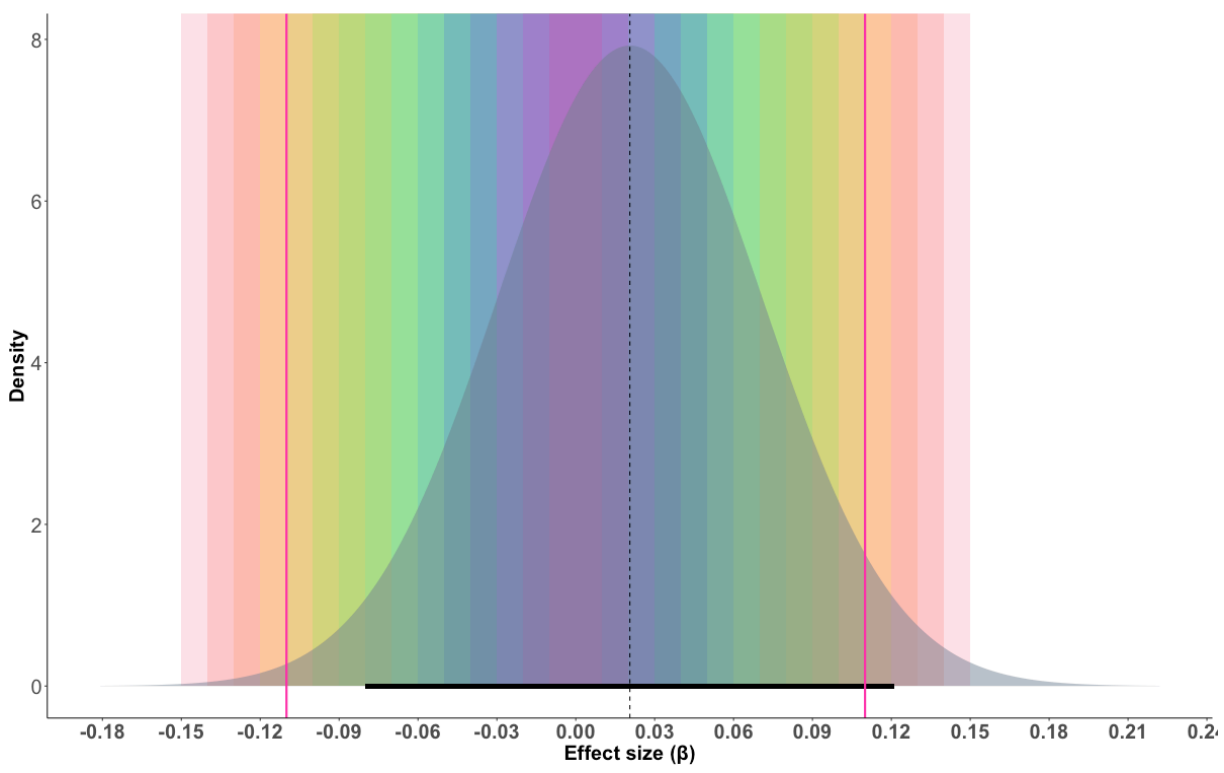

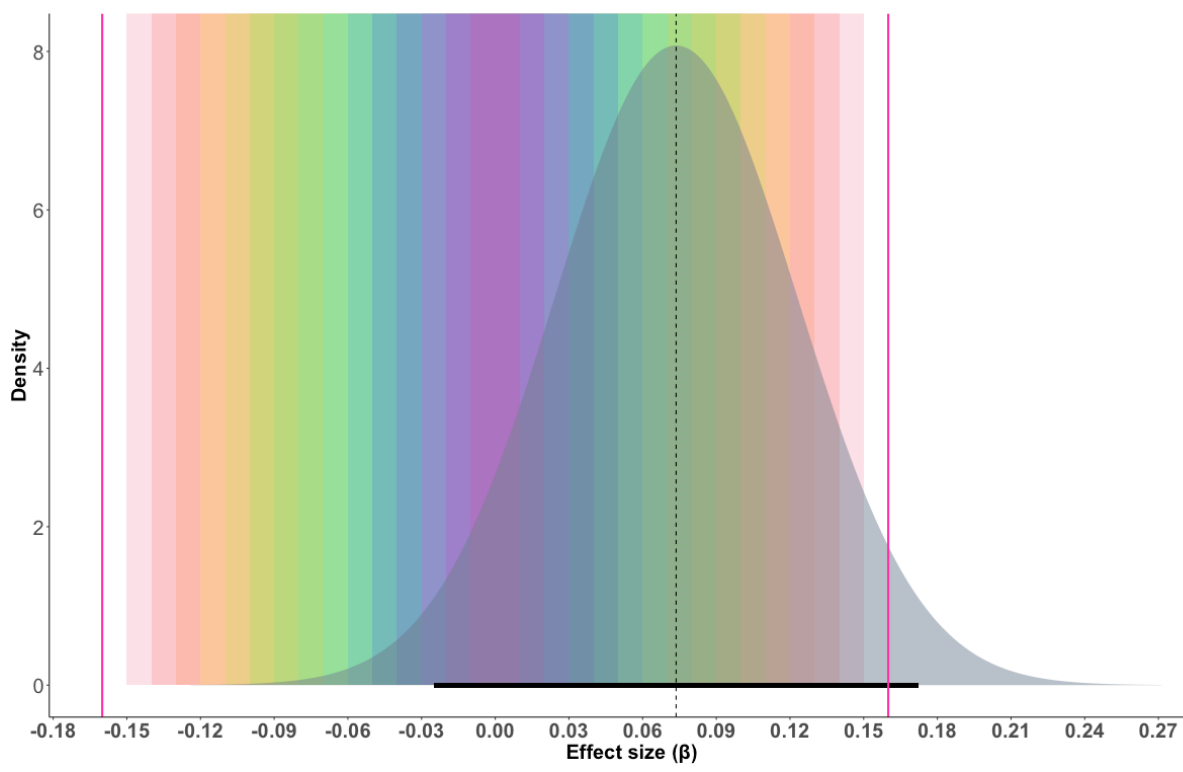

TOST: β = +/-0.11, p(equivalence) = 0.040

ROPE: β = +/- 0.1, 97%

TOST: β = +/- 0.16, p(equivalence) = 0.042

ROPE: β = +/- 0.15, 97%

ToM Network Event 2 (T01)

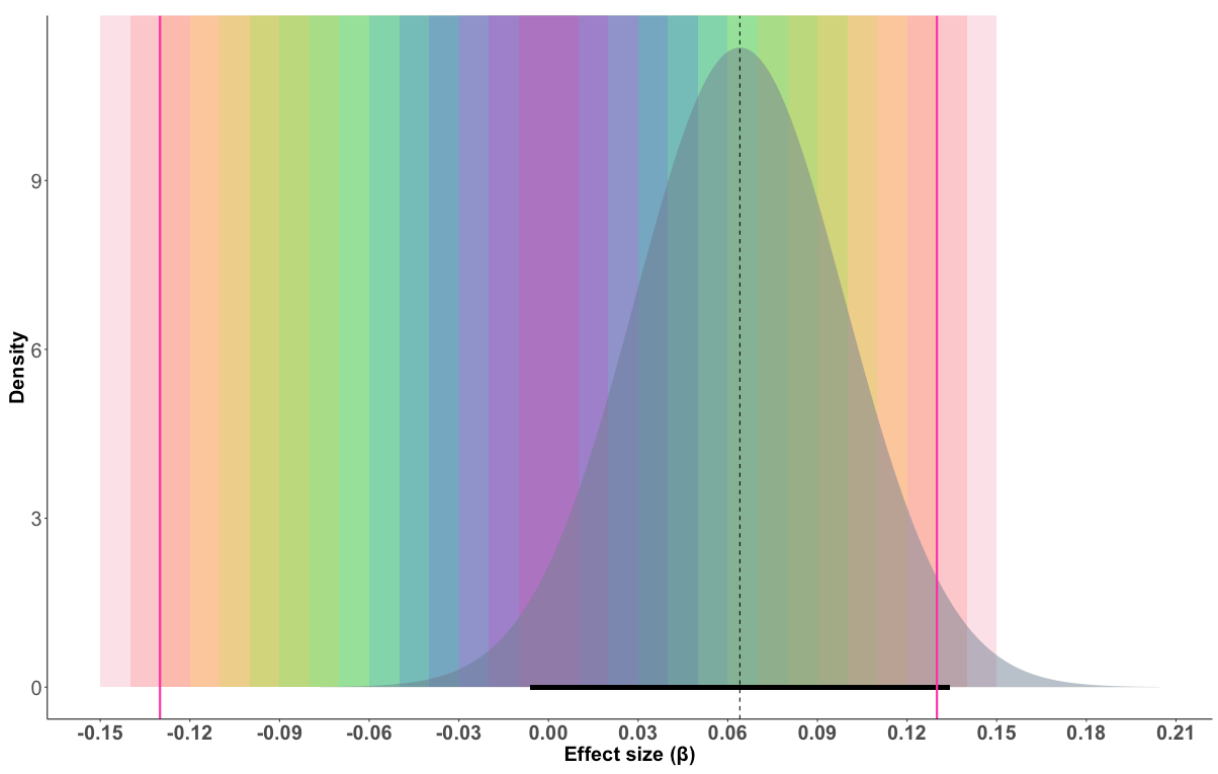

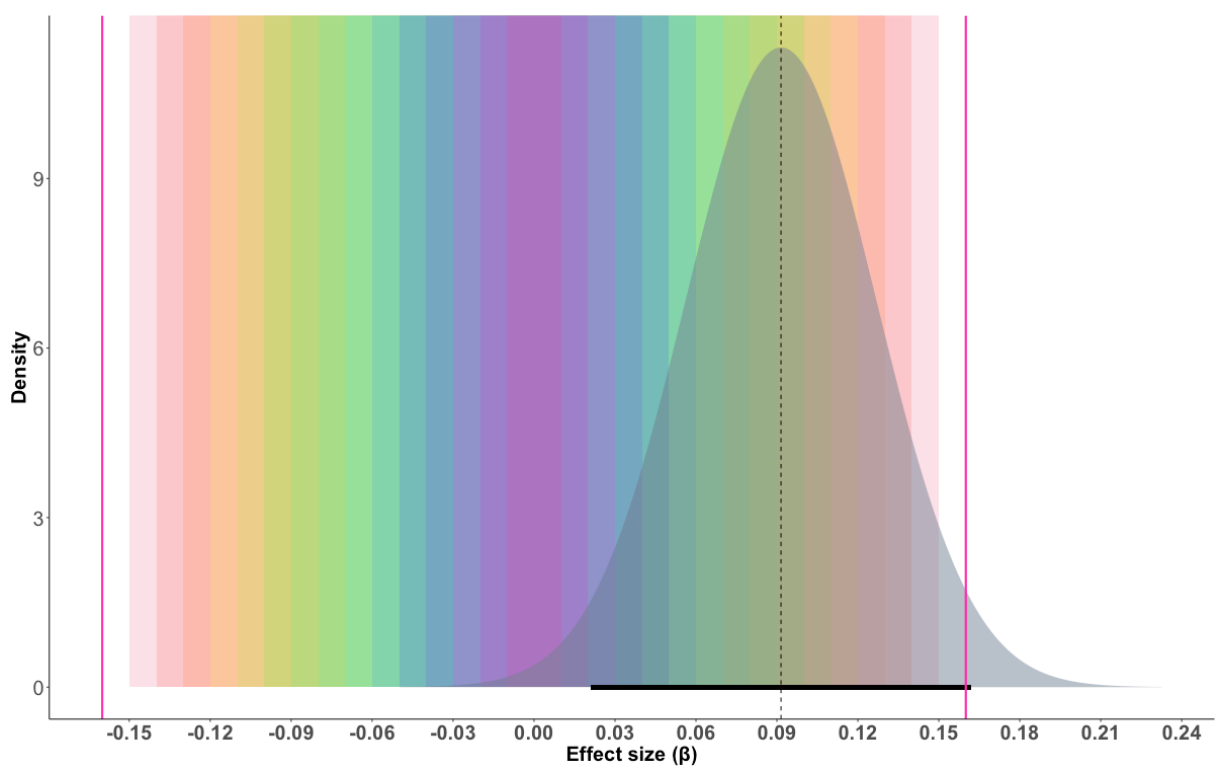

TOST: β = +/- 0.13, p(equivalence) = 0.032

ROPE: β = +/- 0.12, 97%

TOST: β = +/- 0.16, p(equivalence) = 0.028

ROPE: β = +/- 0.15, 97%

RTPJ FuncMat

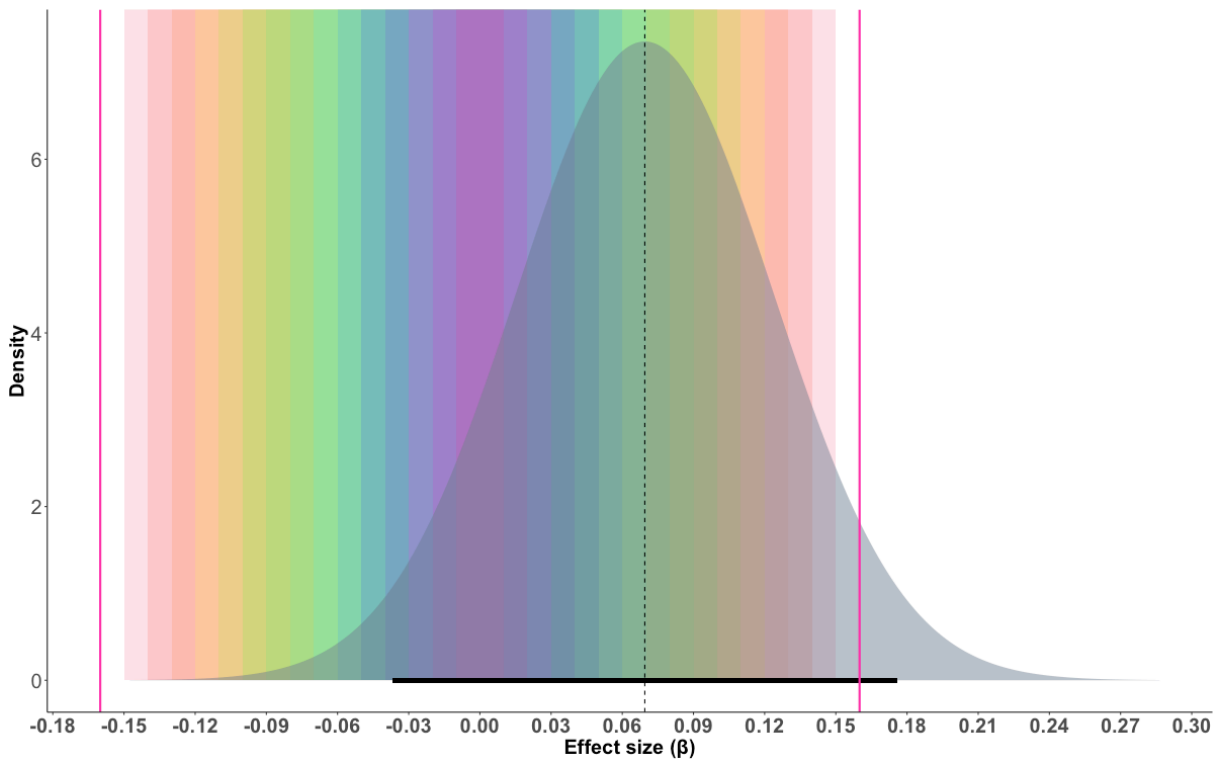

TOST: β = +/- 0.16, p(equivalence) = 0.048

ROPE: β = +/- 0.15, 95%

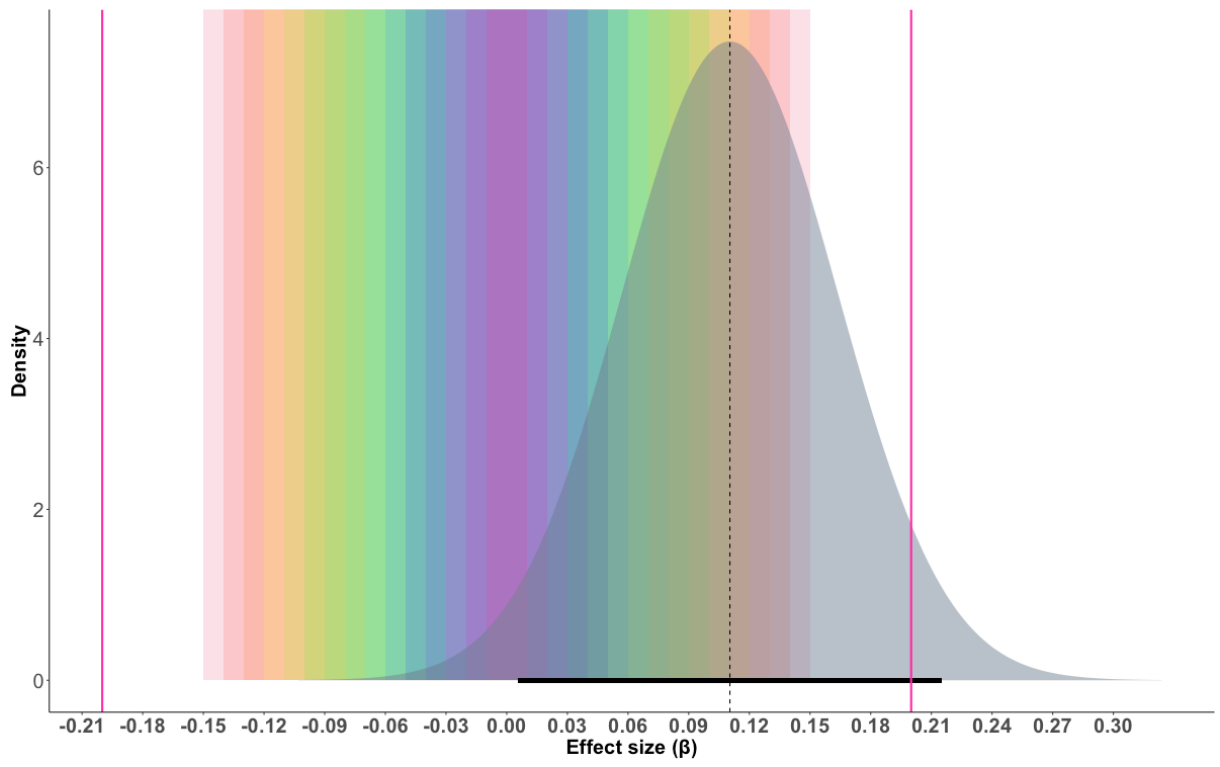

TOST: β = +/- 0.2, p(equivalence) = 0.046

ROPE: β = +/- 0.19, 95%

RTPJ Event 2 (T01)

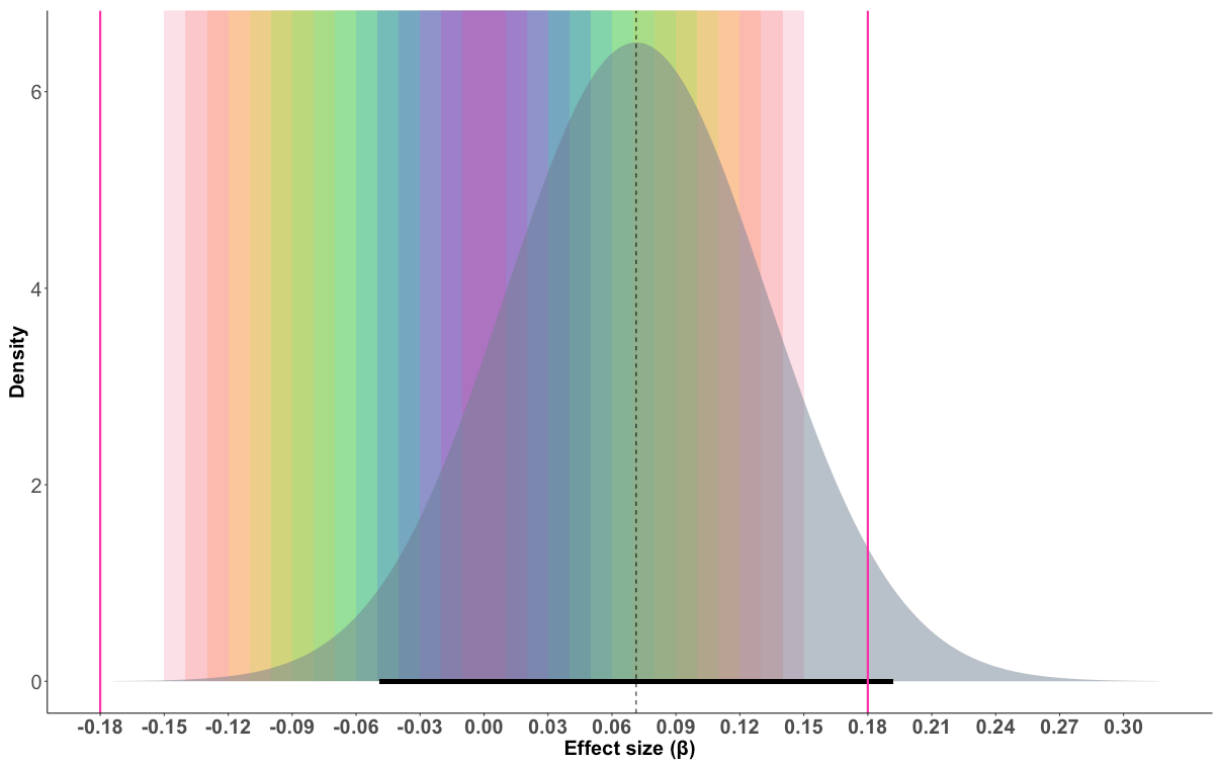

TOST: β = +/- 0.18, p(equivalence) = 0.038

ROPE: β = +/- 0.17, 97%

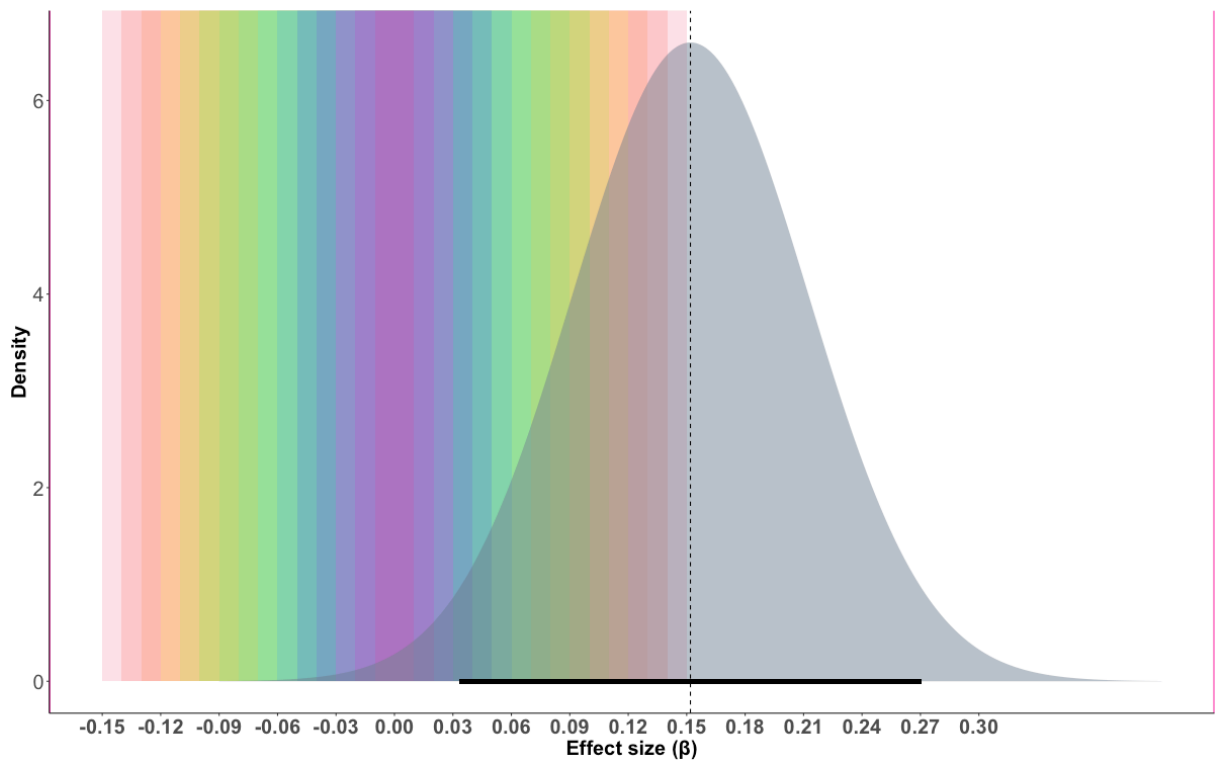

TOST: β = +/- 0.26, 0.037

ROPE: β = +/- 0.25, 97%

RTPJ Event 2 (T04)

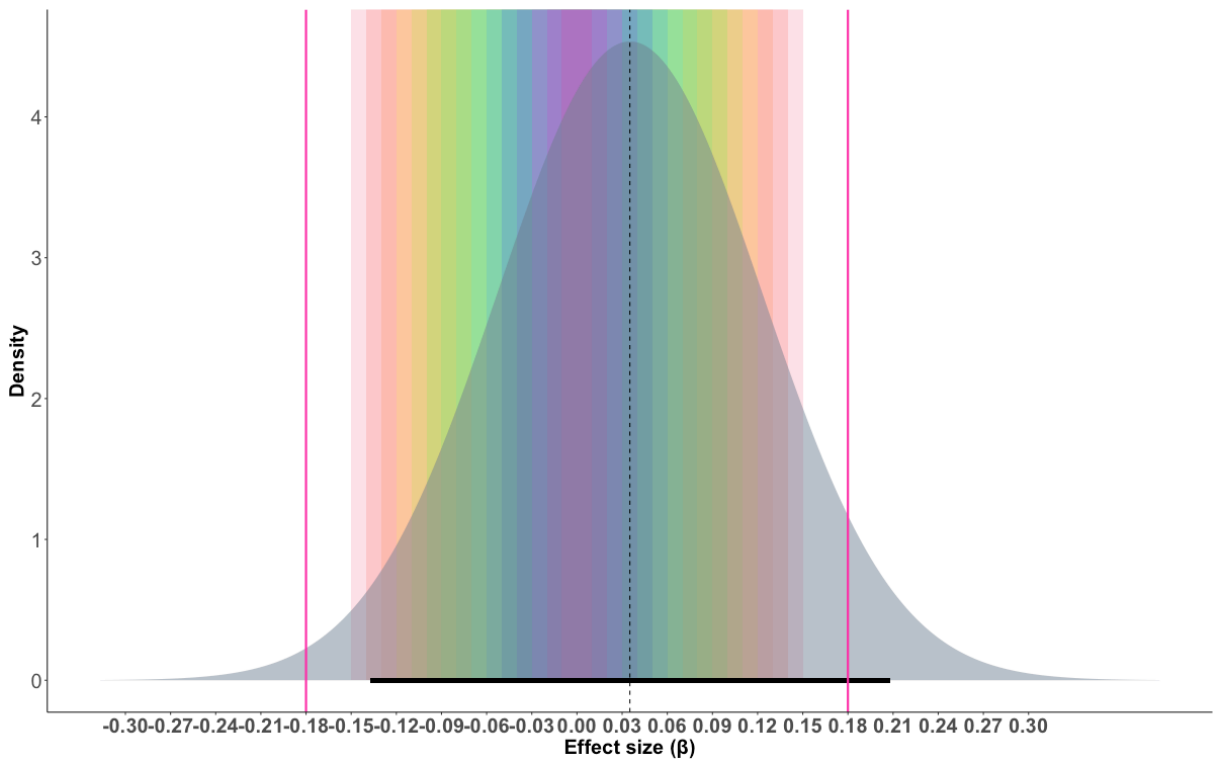

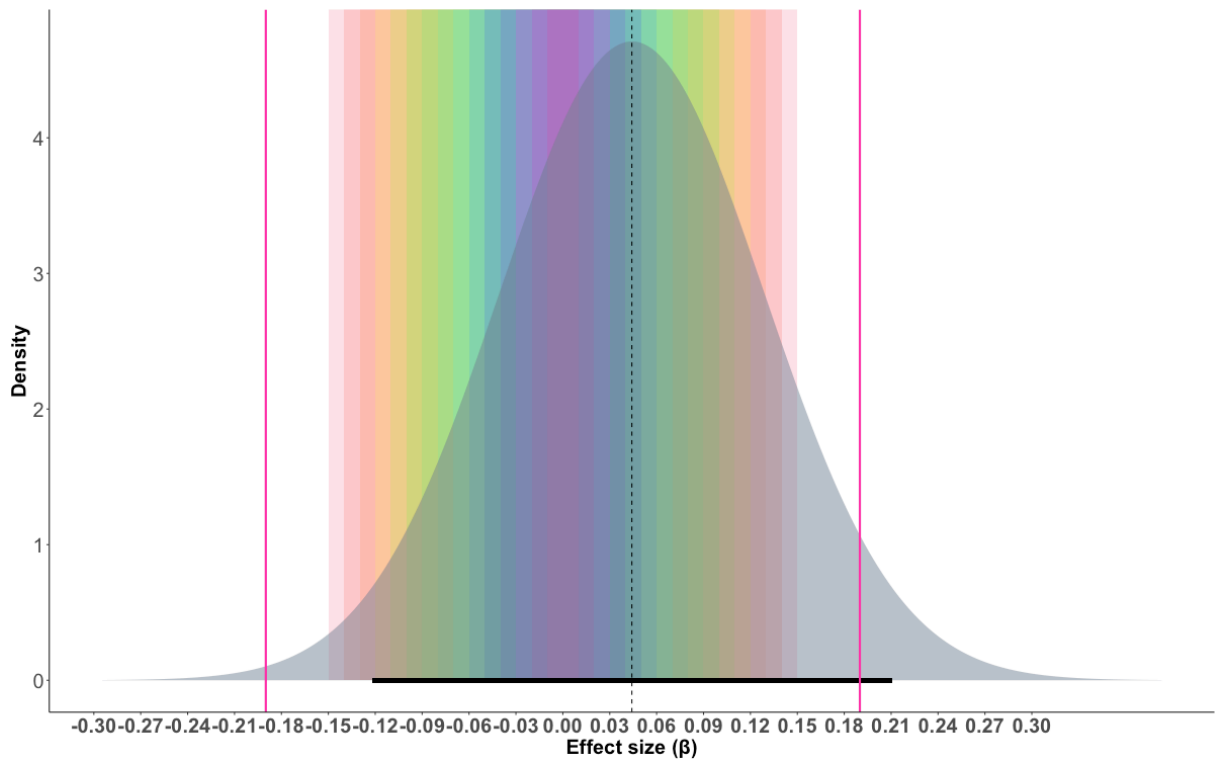

TOST: β = +/- 0.18, p(equivalence) = 0.050

ROPE: β = +/- 0.17, 96%

TOST: β = +/- 0.19, p(equivalence) = 0.043

ROPE: β = +/- 0.17, 96%

ToM Network Event 1 (T02)

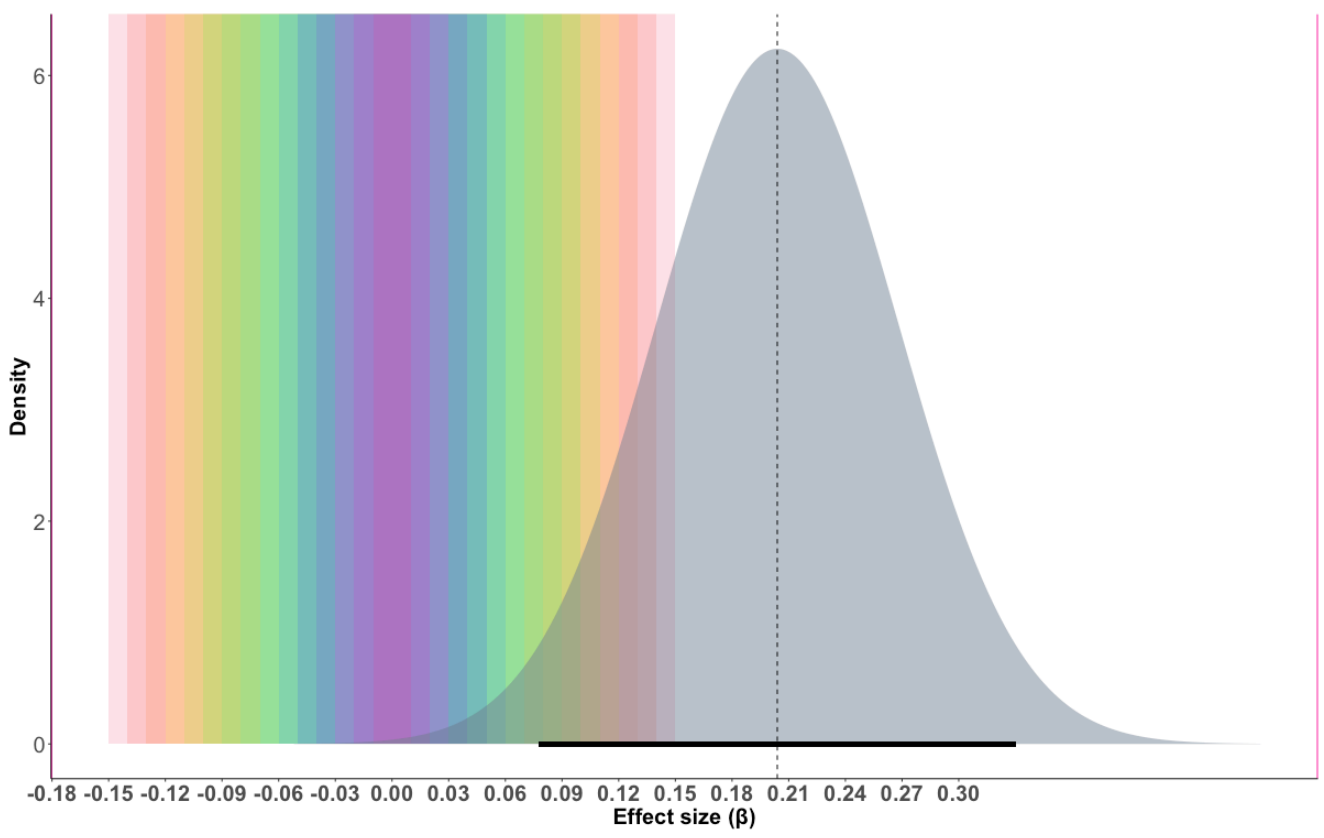

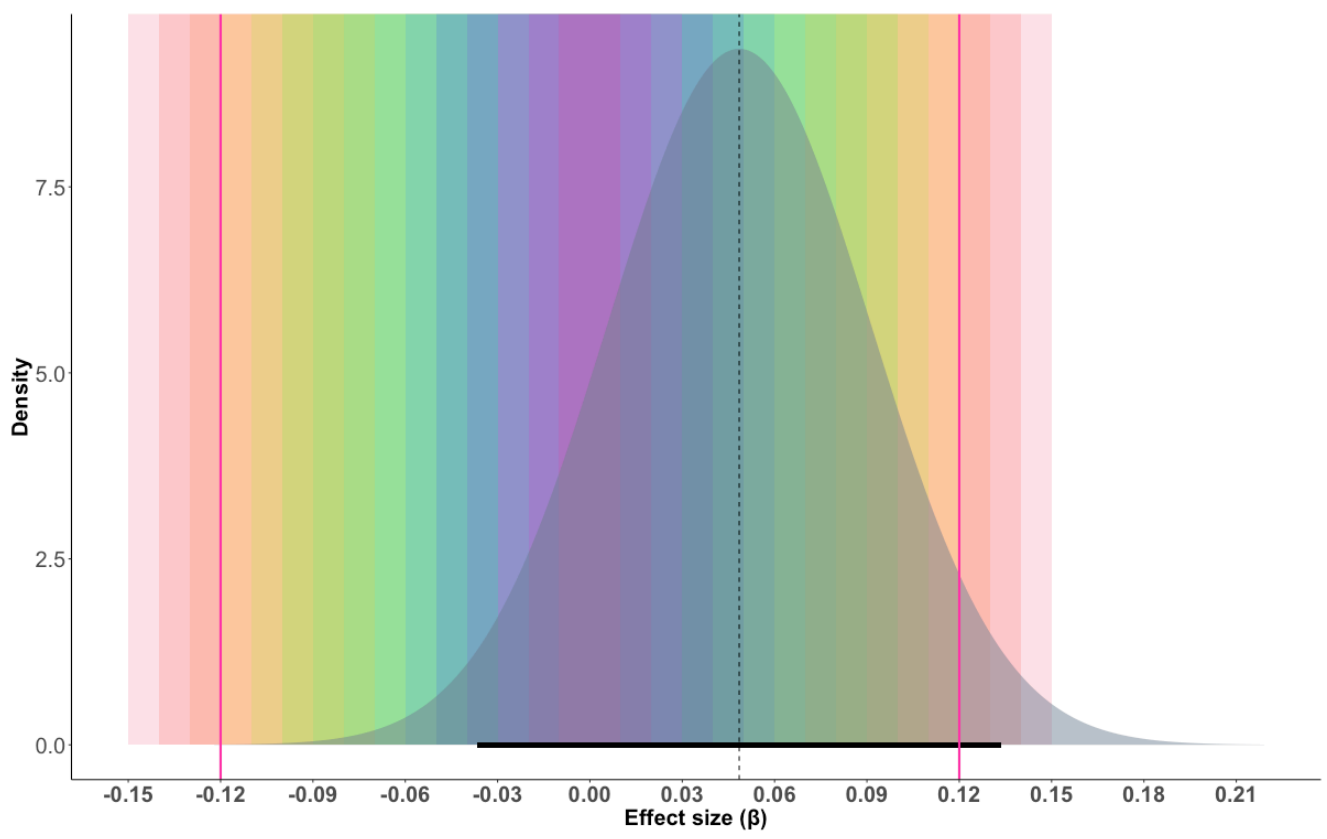

TOST: β = +/- 0.31, p(equivalence) = 0.49

ROPE: β = +/- 0.3, 96%

TOST: β = +/- 0.12, p(equivalence) = 0.049

ROPE: β = +/- 0.12, 97%

RTPJ Event 1(T02)

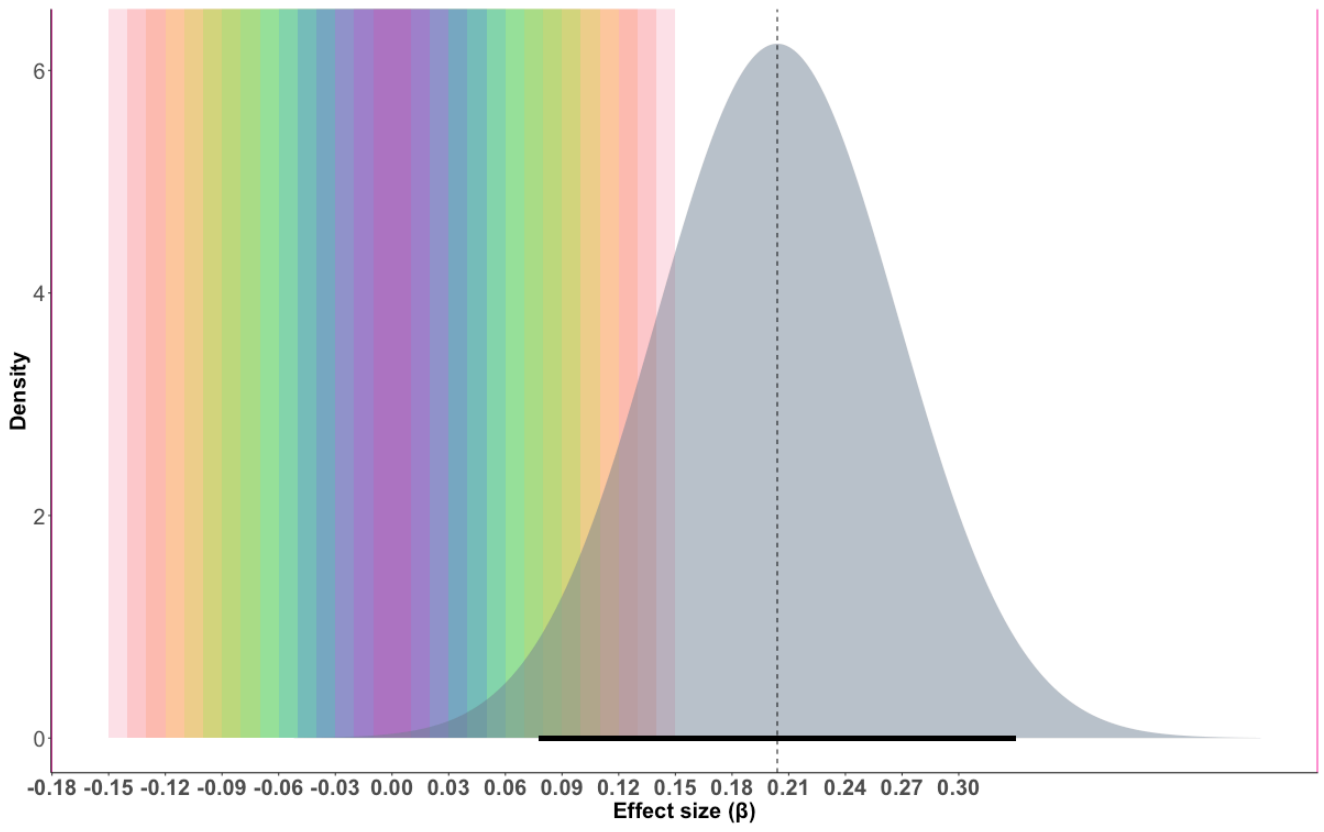

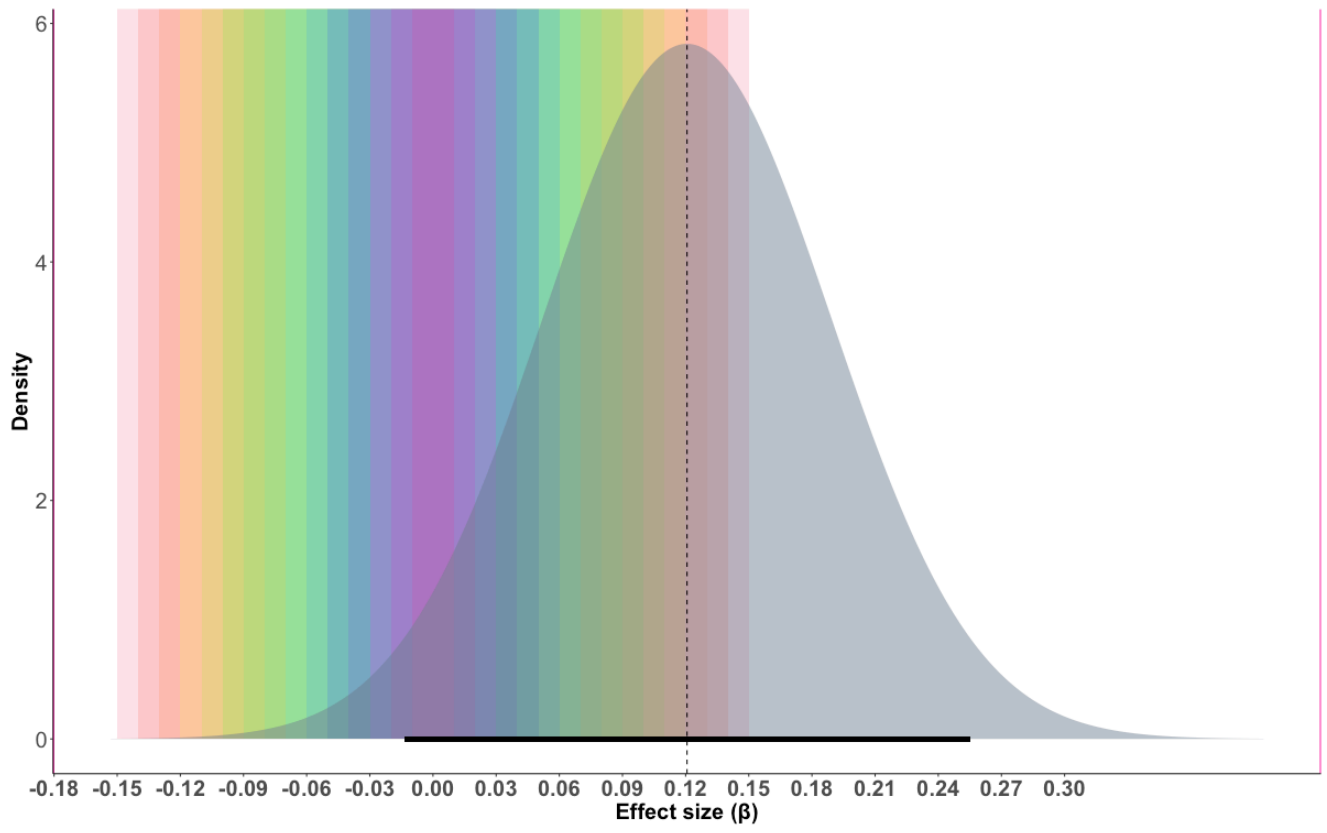

TOST: β = +/- 0.34, p(equivalence) = 0.045

ROPE: β = +/- 0.33, 97%

TOST: β = +/- 0.24, p(equivalence) = 0.041

ROPE: β = +/- 0.23, 97%

Figure S5: Two one-sided tests (TOST) and Region of Practical Equivalence (ROPE) results. Plots show the normal approximation of the sampling distribution per neural metric with the range of equivalence bound values on the x-axis, as tested with TOST. For gestational age effects (left column), the full equivalence bound range tested was -0.2 to 0.2 for neural metrics where pre-registered analyses failed to find statistical evidence for an effect of gestational age and -0.35-0.35 for response magnitude to event 1 (T02) metrics, which varied as a function of gestational age. For effects of age (right column), the full equivalence bound range tested was -0.35 to 0.35. In all plots, equivalence bounds between -0.15 to 0.15 are represented by a different colour every 0.01. The peak of the density plot and vertical dotted line show the most likely effect size (estimated coefficient) for gestational age (left) or age (right) and the distribution spread shows the uncertainty. The vertical hot pink lines indicate the narrowest equivalence bounds for which we could statistically reject the presence of an effect of gestational age (left) or age (right). Neural metrics are organised in order of the strength of the evidence in favour of rejecting the presence of an effect of gestational age (with strongest evidence at the top). The ROPE bounds reported are the narrowest bounds at which at least 95% of the posterior

distribution (as approximated by ROPE) falls; the percentage indicates the exact percentage of the posterior distribution that falls within the bounds specified. ROPE density plots were nearly identical to the TOST plots and so are not shown. Taking the top row as an example: For across-ToM-pain IRC, almost all of the posterior distribution (density plot) falls within TOST and ROPE equivalence bounds of +/- β =0.03 - meaning that we can statistically reject the presence of an effect of gestational age larger than β =0.03, and if there is an effect of gestational age, it is extremely small ($<\beta$ =0.03). For ROPE analyses, 100% of the posterior distribution falls within these bounds. For this measure, there is also evidence in favour of rejecting the presence of an effect of age (right column).

Supplementary Material References

Deferm, W., Tang, T., Moerkerke, M., Daniels, N., Steyaert, J., Alaerts, K., Ortibus, E., Naulaers, G., & Boets, B. (2024). Subtle microstructural alterations in white matter tracts involved in socio-emotional processing after very preterm birth. *NeuroImage: Clinical*, *41*, 103580. https://doi.org/10.1016/j.nicl.2024.103580

Dunstan, J., Ratliff, E. L., & Riggins, T. (2026). Scanning Dos and Don'ts: Using Magnetic Resonance Imaging in Awake Children Aged 3 to 5 years to Assess Brain Structure and Function. *Journal of Visualized Experiments : JoVE*, (229), 10.3791/70023. https://doi.org/10.3791/70023

DuPaul, G. J., Power, T. J., Anastopoulos, A. D., & Reid, R. (2016). *ADHD Rating Scale 5 for Children and Adolescents: Checklists, Norms, and Clinical Interpretation*. Guilford Publications.

Holmboe, K., Larkman, C., Klerk, C. de, Simpson, A., Bell, M. A., Patton, L., Christodoulou, C., & Dvergsdal, H. (2021). The early childhood inhibitory touchscreen task: A new measure of response inhibition in toddlerhood and across the lifespan. *PLOS ONE*, *16*(12), e0260695. https://doi.org/10.1371/journal.pone.0260695

Kruschke, J. K. (2018). Rejecting or Accepting Parameter Values in Bayesian Estimation. *Advances in Methods and Practices in Psychological Science*, *1*(2), 270–280. https://doi.org/10.1177/2515245918771304

Lakens, D., Scheel, A. M., & Isager, P. M. (2018). Equivalence Testing for Psychological Research: A Tutorial. *Advances in Methods and Practices in Psychological Science*, *1*(2), 259–269. https://doi.org/10.1177/2515245918770963

Mullen, E. M. (1995). *Mullen scales of early learning* (pp. 58–64). MN: AGS.

Perner, J., Ruffman, T., & Leekam, S. R. (1994). Theory of Mind Is Contagious: You Catch It from Your Sibs. *Child Development*, *65*(4), 1228–1238. https://doi.org/10.1111/j.1467-8624.1994.tb00814.x

Richardson, H., Lisandrelli, G., Riobueno-Naylor, A., & Saxe, R. (2018). Development of the social brain from age three to twelve years. *Nature Communications*, *9*(1), 1027. https://doi.org/10.1038/s41467-018-03399-2
